# TGF-β signaling in the tumor metabolic microenvironment and targeted therapies

**DOI:** 10.1186/s13045-022-01349-6

**Published:** 2022-09-17

**Authors:** Xueke Shi, Jin Yang, Shuzhi Deng, Hongdan Xu, Deyang Wu, Qingxiang Zeng, Shimeng Wang, Tao Hu, Fanglong Wu, Hongmei Zhou

**Affiliations:** State Key Laboratory of Oral Diseases, National Center of Stomatology, National Clinical Research Center for Oral Diseases, Frontier Innovation Center for Dental Medicine Plus, West China Hospital of Stomatology, Sichuan University, Chengdu, 610041 Sichuan China

**Keywords:** TGF-β signaling, Tumor metabolic microenvironment, Cancer cell, Stromal cell, Host metabolism

## Abstract

Transforming growth factor-β (TGF-β) signaling has a paradoxical role in cancer progression, and it acts as a tumor suppressor in the early stages but a tumor promoter in the late stages of cancer. Once cancer cells are generated, TGF-β signaling is responsible for the orchestration of the immunosuppressive tumor microenvironment (TME) and supports cancer growth, invasion, metastasis, recurrence, and therapy resistance. These progressive behaviors are driven by an “engine” of the metabolic reprogramming in cancer. Recent studies have revealed that TGF-β signaling regulates cancer metabolic reprogramming and is a metabolic driver in the tumor metabolic microenvironment (TMME). Intriguingly, TGF-β ligands act as an “endocrine” cytokine and influence host metabolism. Therefore, having insight into the role of TGF-β signaling in the TMME is instrumental for acknowledging its wide range of effects and designing new cancer treatment strategies. Herein, we try to illustrate the concise definition of TMME based on the published literature. Then, we review the metabolic reprogramming in the TMME and elaborate on the contribution of TGF-β to metabolic rewiring at the cellular (intracellular), tissular (intercellular), and organismal (cancer-host) levels. Furthermore, we propose three potential applications of targeting TGF-β-dependent mechanism reprogramming, paving the way for TGF-β-related antitumor therapy from the perspective of metabolism.

## Background

**Fig. 1:**
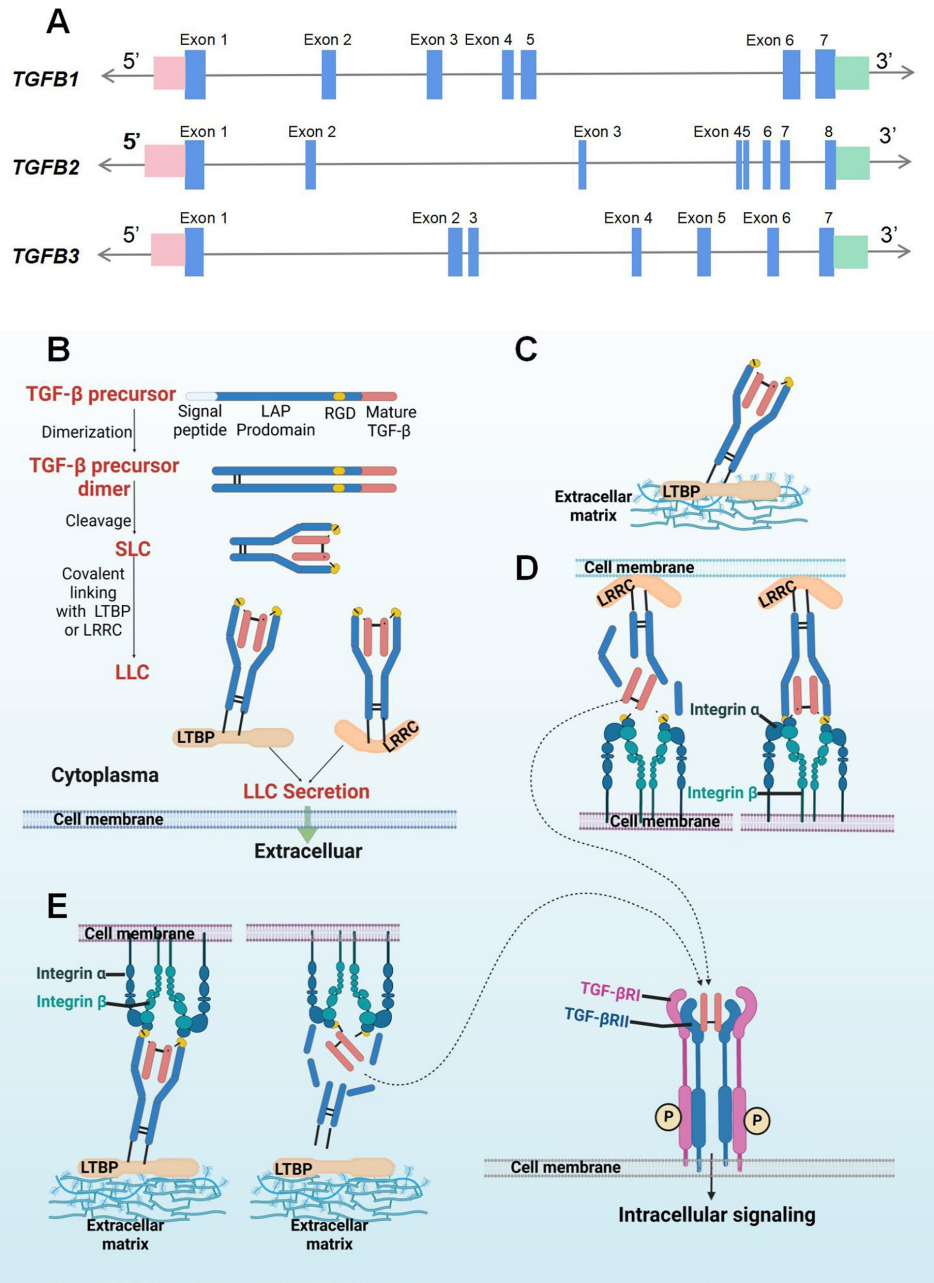
Schematic representation of the gene *TGFB*(A) and protein TGF-β. **A** Gene structure of *TGFB1, TGFB2, and TGFB3*: The blue boxes represent the exons; the 5’- and 3’-untranslated region are marked in pink and green boxes, respectively. **B** Latent TGF-β synthesis and secretion: TGF-β precursor protein consists of a signal peptide, a LAP prodomain, and a mature TGF-β monomer sequence. With the removal of signal peptide, the precursor proteins are dimerized. After proteolytic cleavage, the mature TGF-β dimer remains associated with LAP prodomains and the SLC is formed. Then, SLC links with LTBP or LRRC and thus LLC is generated. The LLC is then secreted into extracellular matrix. **C**, **D** Once released from cells, the TGF-β dimer that is kept inactive by its binding with LTBP, which targets latent TGF-β into the ECM, or with an LRRC molecule that fixes latent TGF-β at the surface of cells. **D**, **E** integrin β, in association with integrin α, can bind with the RGD sequence in the latent TGF-β complex. Then, the increased tension at the interface leads to degradation of the LAP, and the physiological activation of latent TGF-β complexes result in the release of TGF-β ligands. These active TGF-β ligands bind to the TGFβRI/TGFβRII receptor complex at the cell surface, and the intracellular TGF-β signaling is initiated. LAP: latency-associated polypeptide or LAP. SLC: small latent complex; LTBP: latent TGF-β-binding protein; LLC: large latent complex; LRRC: leucine-rich repeat containing; RGD: arginine–glycine–aspartic acid motif. The short solid lines represent covalent bonds, while the short dashed lines are non-covalent bonds

**Fig. 2:**
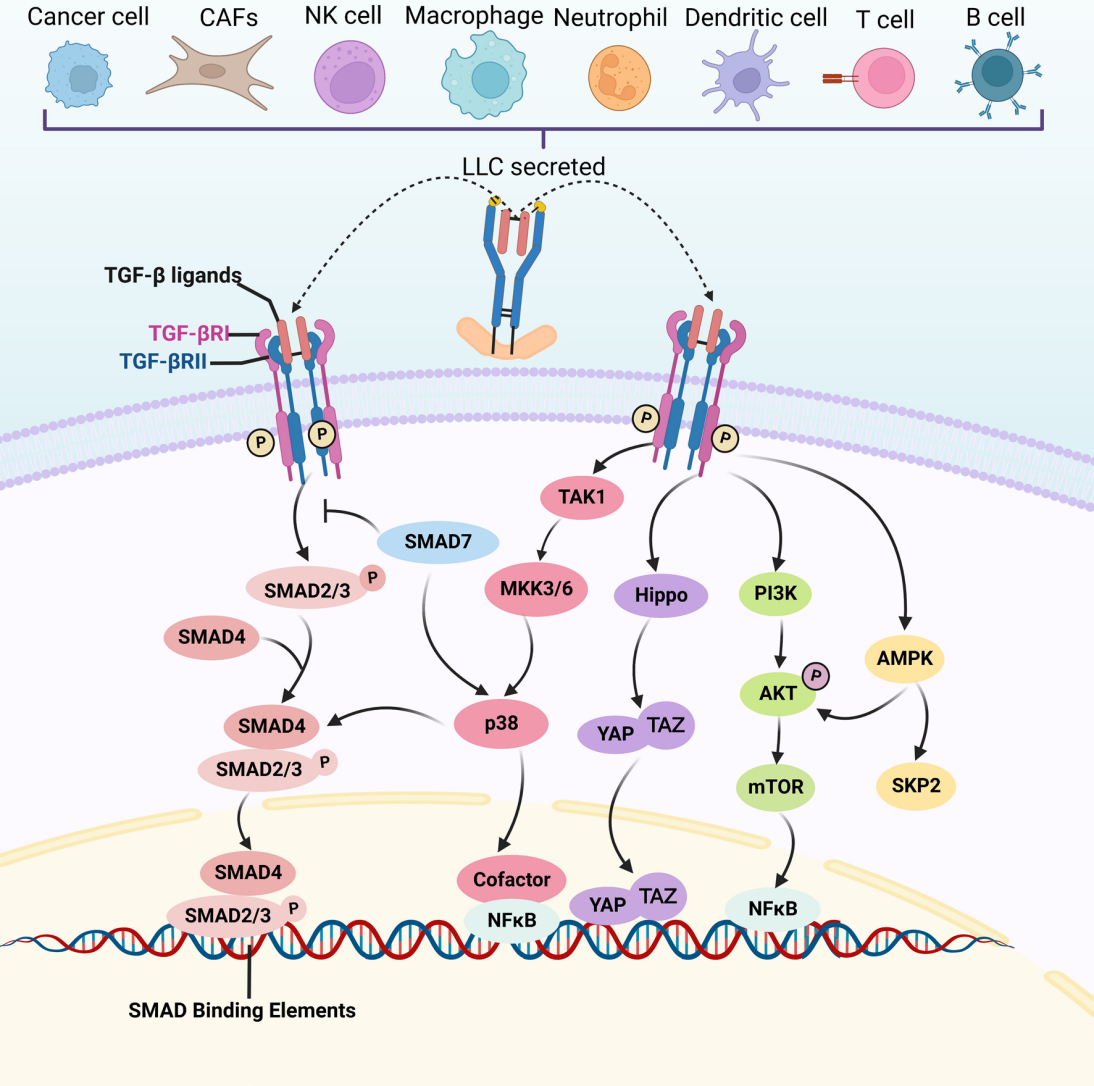
Canonical and non-canonical TGF-β signaling pathway. LLC are produced by various cell types, and TGF-β ligands can be activated and release from the LLC. TGF-β signaling initiates when the TGF-β ligands (TGF-β1,2,3) bind to TGF-βRII.  Then, TGF-βRI is phosphorylated and activates various intracellular signaling cascades. These intracellular pathways of canonical SMAD2/3 pathway and non-canonical TGF-β pathways including MAP kinases (p38), Hippo, PI3K/AKT, and AMPK signaling can subsequently regulate metabolic activities. LLC: large latent complex

Transforming growth factor‐β (TGF-β) signaling is a critical pathway in embryogenesis, tissue homeostasis, and cancer progression [1, 2]. TGF-β ligands consist of TGF-β1, 2, and 3, which are regarded as structurally conserved and comprise a secretion signal peptide, a prodomain, and a mature TGF-β domain [3] (Fig. 1). TGF-β ligands are secreted by almost all cell types, including epithelial cells, fibroblasts, and immune cells [4, 5], and they are inactive and stored in the tumor microenvironment (TME) [6] (Fig. 1). Activated TGF-β ligands initiate downstream signaling components in autocrine- and paracrine-dependent manners [7]. For canonical TGF-β signaling transduction, activated TGF-β ligands bind to the tetrameric receptor complex composed of TGF-β type I and II receptor. TGF-βRII promotes the phosphorylation of TGF-βRI, propagating signals via the phosphorylation of SMAD2/SMAD3 to trigger a cascade response. Phosphorylated SMAD2/SMAD3 proteins complexed with SMAD4 then translocate into the nucleus, where the complex binds to a specific DNA region, namely SMAD-binding elements, to regulate gene transcription. For non-canonical TGF-β signaling pathways, TGF-β ligands can activate non-SMAD signaling pathways, including mitogen-activated protein kinase (MAPK), Hippo, phosphoinositide 3-kinase (PI3K)/AKT, and AMP-activated protein kinase (AMPK) signaling (Fig. 2).

TGF-β signaling can inhibit cell growth in benign cells but promote cancerous biological behaviors in cancer cells; this phenomenon is known as the TGF-β paradox [8]. Paradoxical TGF-β functions are cell- and context-dependent with a specific molecular mechanism [9, 10]. In benign cells, TGF-β can induce cell cycle arrest, differentiation, cell apoptosis, and autophagy [11]. Besides, the production of paracrine factors in stromal fibroblasts and inflammatory cell can be inhibited via TGF-β secreted by benign epithelial cells [11]. However, once benign cells transform into cancer cells, TGF-β signaling contributes to cancer progression by regulating immune escape [12], fibrosis [10], and epithelial–mesenchymal transition (EMT) [13]. Metabolic reprogramming is a hallmark of cancer [14], and increasing evidence has verified that cancer is a metabolic disease involving abnormal metabolic alterations [15], and these alterations served as an “engine” of cancer progression. Recently, the metabolic effect of TGF-β has attracted increased attention, and researchers have realized that TGF-β is a tumor and host metabolic reprogramming cytokine [16, 17]. In this review, we focus on recent insights into metabolic reprogramming in cancer cells and tumor-infiltrated stromal cells, including cancer-associated fibroblasts (CAFs), innate and adaptive immune cells, endothelial cells (ECs), adipocytes, and erythrocytes; then, we address how the TGF-β signaling pathway is involved in the tumor metabolic microenvironment (TMME) at the cellular and tissular (intercellular) level. Subsequently, we elaborate on how TGF-β signaling alters host metabolism of cancer patients at the organismal level. We further discuss the potential of targeting TGF-β-related metabolic reprogramming to fail the “engine” and increase cancer treatment efficiency from the perspective of metabolism.

## Characteristics of the tumor metabolic microenvironment

Reprogrammed metabolic activities within tumors yield a unique microenvironment. Wang et al. and García-Cañaveras et al. proposed the designation of the “TMME” to describe this unique microenvironment [18, 19]. However, they did not provide a concise definition. Synonyms proposed in other studies, such as “metabolic alterations in the tumor microenvironment,” “metabolic landscape of the tumor microenvironment,” and “metabolic profile of tumor,” are also common [20–22]. Here, we review the literature on the TMME and these synonyms to elaborate on the concept of the TMME.

Cellular and non-cellular components construct the TMME. Instead of working alone, cancer cells interact with stromal cells, extracellular matrix, soluble factors, and signaling molecules to benefit their own progression. All of these cellular and non-cellular components and their interactions form the TME [23]. Stromal cells form constitutive cellular components of the TME and include CAFs, infiltrated immune cells, ECs, and adipocytes. These stromal components in the TME are recruited from the normal surrounding tissues and can be “educated” to a cancer-associated phenotype that is non-malignant but facilitates cancer cell progression. An increasing number of studies have revealed that metabolic alterations change the cell morphology and function [24–27]. Therefore, metabolic alterations, commonly called metabolic reprogramming, deserve to be studied to illustrate how they function in the tumor, with the goal of targeting cancer progression from the perspective of metabolism.

Metabolism refers to all the biochemical reactions that occur in the human body and can be classified according to different criteria. Metabolism controls various biological processes across cellular, tissular, and organismal levels to sustain human life [28]. At the cellular level, tumor metabolic reprogramming occurs in cancer and stromal cells, and the network comprises metabolic genes, metabolic enzymes, metabolic intermediates, and signaling molecules. At the tissular level, metabolic interactions between cell types occur and are called metabolic coupling, such as epithelial–stromal metabolism coupling [29]. At the organismal level, tumors, as malignant organs [30], can secret various factors that influence host metabolism. Furthermore, based on the type of nutrients, metabolism can also be classified into glucose, lipid, and amino acid metabolism. Metabolism consumes glucose, lipids, and amino acids to produce energy via complex biological oxidation, and the metabolic intermediates of these types of metabolism provide building blocks of nucleotides that are essential for cancer growth. In addition, the metabolism of these complex macromolecules can fall into two types of chemical reactions known as anabolism or catabolism [31]. The anabolic pathways refer to the synthesis of complex macromolecules to meet the demand of the extracellular and intracellular biomass, while catabolic pathways aim at the degradation of complex macromolecules to produce energy. After the uptake of nutrients from blood, intracellular metabolic kinases initiate signaling cascades and direct the entry of these nutrients into anabolic or catabolic pathways [32]. Collectively, cells in a tumor undergo anabolism and catabolism reprogramming of glucose, lipid, and amino acid.

One of the hallmarks of cancer is the abnormal regulation of cellular metabolism, which reconstitutes the TMME [14, 33]. Cancer and stromal cells in a tumor adapt their anabolism and catabolism of glucose, lipid, and amino acid to sustain the energy and macromolecules needed for cancer growth [34]. The metabolism of cells in the tumor is rewired; then, the nutrients, substrates, metabolic intermediates, and final metabolites are unbalanced and accumulate intracellularly or extracellularly, thus forming a reprogrammed metabolic environment in the tumor, i.e., TMME. Therefore, the intracellular metabolic reprogramming of cells and abnormal extracellular metabolite accumulation are all included in the TMME. For example, the TME is always hypoxic; therefore, cancer and stromal cells tend to be highly glycolytic and produce a large amount of lactate, constructing an acidic TMME. Moreover, cancer cells competitively capture nutrients of glucose, lipid, and amino acid in the TME and ultimately use these nutrients for their cancer growth, invasion, and metastasis [35, 36]. Hence, we summarize the characteristics of the TMME in terms of the following three aspects: (1) hypoxia; (2) high acidity or acidosis; and (3) nutrient deprivation of glucose, lipid, and amino acid. These characteristics of the TMME reciprocally switch the metabolism of stromal cells, including fibroblasts and immune cells, and contribute to the formation of a tumor-promoting immunosuppressive TMME.

TGF-β signaling is one of the most important pathways influencing tumor initiation [37], growth [38], and metastasis [39]. Consistently, we observed that TGF-β activation is essential for lung metastasis growth in head and neck squamous cell carcinoma [40]. Currently, there are many agents designed to target TGF-β signaling that have achieved satisfying clinical cancer treatment efficacy [10]. Recent studies have demonstrated that TGF-β signaling is a main metabolic driver in the TMME and thus plays a crucial role during cancer progression [27, 41]. In the following sections, we will elaborate on metabolic reprogramming at the cellular, tissular (metabolic coupling), and organismal levels (host metabolism). Then, we will highlight the TGF-β-dependent mechanism involved in these metabolic alterations, aiming to widen our scope of knowledge on the TMME and to facilitate the development of more cancer therapies from the perspective of TGF-β-dependent metabolism.

## TGF-β-dependent metabolism of cancer cells and targeted therapies

### Glucose metabolism

#### Glucose metabolism phenotypes of cancer cells

In the 1920s, Otto Warburg found that even in the presence of oxygen, cancer cells still prefer glycolysis but not the TCA cycle, and this phenomenon is called the Warburg effect. One glucose molecule generates 30–32 adenosine triphosphate (ATP) in the TCA cycle but only 2 ATP through glycolysis. Why would cancer cells choose inefficient glycolysis instead of the TCA cycle? At first, Otto Warburg assumed that mitochondrial function is impaired in cancer cells [42]. However, Weinhouse et al. [43, 44] showed that oxidative phosphorylation (OXPHOS) can occur in cancer cells at a speed similar to normal cells. Therefore, glycolysis is accelerated in cancer cells but is not related to damaged mitochondria, and OXPHOS is still the main ATP energy source in most cancer tissues. Hence, another question was raised: What is the meaning of enhanced glycolysis [45]? Researchers observed that glycolysis provides precursors for the synthesis of biomass, including lipids, nucleotides, and amino acids, which are essential for cell mitosis [46, 47]. Additionally, lactate produced by the Warburg effect contributes to the acidic TMME, which leads to increased proliferation, apoptosis resistance, and metastasis of cancer cells [48]. Therefore, the main function of the Warburg effect is to sustain cancer cell biological behavior, while the tricarboxylic acid (TCA) cycle produces sufficient ATP for cell survival.

Even though mitochondria are not impaired, researchers have demonstrated that enzymes in the TCA cycle can be altered in the TMME. Mutation and expression changes in succinate dehydrogenase (SDH), isocitrate dehydrogenase, fumarate hydratase, and malate dehydrogenase, are related with progression of colorectal cancer and other types of cancer [49–51]. These studies suggest that mutation and changes in the total amount of TCA cycle enzymes may accelerate cancer progression.

The pentose phosphate pathway (PPP) is an offshoot of glycolysis, diverging at the level of glucose-6-phosphate (G6P) and playing a crucial role in cancer cell glucose reprogramming [52, 53]. PPP consists of two phases, i.e., the oxidative phase and non-oxidative phase. Reprogramming of the oxidative phase of PPP is mainly achieved through the oxidative phase enzyme G6P dehydrogenase (G6PD), which serves as the “gateway” between glycolysis and PPP. G6PD is expressed at higher rates in ovarian [54] and renal cancer [55], which indicates greater PPP flux. G6PD was reported as an enzyme that promotes cisplatin resistance [56], and G6PD inhibition increases chemotherapy sensitivity [57]. Non-oxidative phase reprogramming involves changes in two enzymes: transketolase and transaldolase. Transketolase has been reported to be elevated in breast, prostate, and lung cancer cells [58–60], and increased transaldolase levels have been found in hepatocellular carcinoma [61]. In summary, elevated expression of enzymes in PPP indicates that cancer cells tend to exhibit increased flux into this pathway, thus sustaining their proliferation and survival by producing biomass building blocks.

Glycogen is a multibranched polysaccharide of glucose that serves as energy storage and provides an immediate source of glucose to support the energy requirements of cells. Glycogen metabolism consists of glycogenesis and glycogenolysis. Glycogenesis has been reported to be upregulated in cancers including clear cell renal cell carcinoma [62], ovarian clear cell carcinoma [63], and melanoma [64]. Hypoxia, as a hallmark of cancer, resulted in glycogenesis via glycogen synthase induction and that glycogen increased cancer cell survival under hypoxia and nutrient restriction [65, 66]. Therefore, glycogenesis promotes cancer cell survival under hypoxic conditions [67, 68]. Then, how does glycogen protect cancer cell death? Glycogenolysis, the process by which glycogen is converted to glucose-1-phosphate and then to G6P and enter the glycolytic pathway, offers another energy source for tumors under nutrient restraint stress [69]. Liu et al. [70] found that dysregulated glycogenolysis boosted glycogen storage, and glycogen can trigger a tumor-promoting signaling pathway to avoid cancer cell death and accelerate cancer progression in a non-metabolic manner. In addition, glycogen can provide energy for cancer cells in nutrient-restricted TMME [71]. Taken together, upregulated glycogen synthesis and dysregulated glycogenolysis contributed to glycogen accumulation in cancer cells, thus protecting cancer cell from death under hypoxia and providing cancer cell energy under nutrient deprivation. Glycogen metabolism could be a promising anticancer target.

#### TGF-β signaling in glucose metabolism of cancer cells

EMT is essential for malignant transformation and metastatic formation, and it is characterized by morphological alterations during which apical–basal polarized epithelial cells are transformed into cells with a mesenchymal spindle shape [72]. EMT is induced through canonical or non-canonical TGF-β signaling, or their cooperation [73–75]. Recent studies have shown that glucose metabolic rewiring is concomitant with EMT, and they are mutually reinforcing. This section illustrates how TGF-β-induced EMT and metabolic alterations interact with each other (Table 1).

**Table 1:** TGF-β-dependent glucose metabolic reprogramming and ROS regulation of cells in cancer

	Signaling components	TGF-β-dependent metabolic component change	Metabolic reprogramming/cell biology influenced	Cancer type	Experimental status	Ref.
*Cancer cell*						
Glycolysis	TGF-β1-GLUT1	TGF-β enhanced the expression of GLUT1	Increased glucose uptake, induced EMT	BC, pancreatic carcinoma	In vitro human cell culture	[76–78]
	ANGPTL2-α5β1-TGF-β-ZEB1-GLUT3	ANGPTL2 increased GLUT3 expression by TGF-β signaling activation	Elevated glycolysis, promoted metastasis and EMT	NSCLC	In vitro human cell culture	[266]
	TGF-β1-HK2	TGF-β1 increased mRNA expression of HK2	Increased glycolysis; Promoted proliferation and metastasis	Neuroblastoma and gallbladder cancer	In vivo mouse model	[84]
	TGF-β1-PFKFB3	TGF-β1 elevated PFKFB3	Increased glucose uptake, glycolytic flux, and lactate production; Promoted invasion	Glioma and pancreatic carcinoma	In vitro human cell culture	[84, 87]
	TGF-β-TGFIF/PKM2	TGFIF and PKM2 were increased under TGF-β1 stimulation	Promote Warburg effect and promoted EMT	Colorectal cancer, lung carcinoma	In vitro human cell culture	[89, 90]
	TGF-β-mTOR-p70s6k-PKM2	TGF-β1 increased the expression of PKM2	Influenced glycolysis and Warburg effect, induced EMT	Cervical cancer	In vitro human cell culture	[267]
TCA cycle	SDHB-TGF-β-SMAD3/SMAD4-SNAL1	SDHB deficiency activated TGF-β signaling	Induced mitochondrial enzyme SDH dysfunction; Increased invasion and migration via EMT	Colorectal cancer	In vitro human cell culture	[96]
Pentose phosphate pathway	TGF-β1-FOXM1-HMGA1-G6PD-TGF-β1	Increased the expression of G6PD via TGF-β signaling activation	Enhanced PPP and thus increased cisplatin resistance	NSCLC	In vitro human cell culture	[101]
Glycogen Synthesis	TGF-β1-LEFTY2-SGLT1 and GYS1	inhibited LEFTY2 expression, and decreased SGLT1 and GYS1	Negated glycogen formation	Endometrial cancer	In vitro human cell culture	[102]
	GSK-3β-TGF-β/SMAD3 signaling	GSK-3β inhibited activity of SMAD3 under TGF-β stimulation	Not mentioned	HCC	In vitro human cell culture	[103]
	TGF-β-GSK-3β-HNF4α	Inhibited GSK-3β and then hamper the activation of tumor suppressor HNF4α	Promoted EMT	HCC	In vitro human cell culture	[104]
ROS	TGF-β2-catalase-H_2_O_2_	Reduced the amount of H_2_O_2_ by catalase overexpression	Regulated H_2_O_2_ redox balance and acquired aggressive dissemination phenotype	NSCLC	In vitro bovine and human cell culture	[268]
	TGF-β1-ROS-ERK	Activated ERK signaling by TGF-β1-mediated ROS production	Downregulated ATP consumption, inhibited cell growth, and induced apoptosis	Colon cancer	In vitro human cell culture	[269]
*Fibroblast*						
Glycolysis	TGF-β-CAV-1-TGF-β activation	Downregulated CAV-1 and activated TGF-β signaling in turn	Promoted RWE, increased glycolysis and decreased OXPHOS	Skin cancer	In vitro human cell culture; in vivo mouse model	^[16]^
	TGF-β1-IDH3α	Downregulated IDH3α by TGF-β1 treatment	Increased glycolysis and switched from oxidative phosphorylation to aerobic glycolysis	Melanoma	In vitro human cell culture; in vivo mouse model	[150]
TCA cycle	TGF-β-PDK1	Activated PDK1	Decrease entry of pyruvate into the TCA cycle	Lymphoma and renal cell carcinoma	In vitro human cell culture	[169, 170]
*NK cell*						
Glycolysis and OXPHOS	GARP-TGF-β-mTOR1-CD71	Increased expression of GARP activated TGF-β signaling and then downregulated mTOR1 and CD71	Reduced glycolysis and OXPHOS; Damaged effector function of NK cells	BC	In vitro human cell culture	[219]
*Macrophage*						
OXPHOS	TGF-β ligand	May enhance OXPHOS by TGF-β signaling activation	Promoted macrophage polarization to M2-phenotype and inhibited its immune toxicity	Melanoma	In vitro mouse cell culture	[221]
*T cell*						
OXPHOS	TGF-β-SMAD-ATP synthase-IFNγ	Inhibited ATP synthase activity	Inhibited IFNγ production and diminished T cell function	Pancreatic, lung, urothelial, and cholangiocellular cancers	In vitro human cell culture	[235]

*GLUT* glucose transporter; *HK2* hexokinase 2; *PFKFB3* 6-phosphofructo-2-kinase; *TGIF2* TGF-β-induced factor homeobox 2; *H3K9* histone H3 lysine 9; *ANGPTL2* angiopoietin-like protein 2; *ZEB1* zinc finger E-box-binding homeobox 1; *PKM2* pyruvate kinase M2; *TCA cycle* Tricarboxylic acid cycle; *OXPHOS* oxidative phosphorylation; *SDHB* succinate dehydrogenase B subunit; *SDH* succinate dehydrogenase; *FOXM1* forkhead box M1; *HMGA1* high mobility group A; *G6PD* glucose-6-phosphate dehydrogenase; *LEFTY2 *endometrial bleeding-associated factor; *GSK-3β* glycogen synthase kinase 3; *HNF4α* hepatocyte nuclear factor 4; *mtDNA* mitochondrial DNA; *Cyt C* cytochrome c; *ROS* reactive oxygen species; *ERK* extracellular signal-regulated kinase; *PCK1* phosphoenolpyruvate carboxykinase 1; *CAV-1* caveolin-1; *IDH3α* isocitric dehydrogenase 3; *PDK1* pyruvate dehydrogenase kinase 1; *GARP* glycoprotein A repetitions predominant; *BC* breast cancer; *NSCLC* non-small cell lung cancer; *HCC* hepatocellular carcinoma; and *PDAC* pancreatic ductal adenocarcinoma

The first step of glycolysis is the entry of glucose into the cytoplasm, and the glucose transporter (GLUT) family, including GLUT1 and GLUT3, mediates the first step in cellular glucose usage. TGF-β induces GLUT1 overexpression in pancreatic ductal adenocarcinoma (PDAC), breast cancer, glioma, and gastric cancer cells [76, 77]. GLUT1 expression is correlated with EMT markers, including E-cadherin and vimentin, and it is accompanied by increased glucose uptake during TGF-β-induced EMT in breast cancer cells [76, 78]. Inhibiting glucose uptake by resveratrol in gastric cancer cells abrogates glucose uptake and tumor growth in a dose- and time-dependent manner [79]. However, silencing *GLUT1* induces chemoresistance in breast cancer cells [80]; therefore, the efficacy of targeting GLUT1 should be evaluated. GLUT3 shows upregulated expression during TGF-β-induced EMT in non-small cell lung cancer (NSCLC) cells. Inhibiting GLUT3 expression reduces glucose import and the proliferation of NSCLC cells [81]. Furthermore, GLUT3 has been identified as a transcriptional target of ZEB1 that facilitates EMT [81]. These results demonstrate that TGF-β upregulates the GLUT family, and glucose uptake is thus enhanced and exhibits a role in promoting malignant biological properties of cancer cells, including EMT, chemoresistance and proliferation. GLUTs could be potential targets for cancer.

Hexokinase 2 (HK2) is the first key enzyme in glycolysis and phosphorylates glucose to generate G6P. HK2 has been reported to be required for tumor initiation in mouse models and is related to cancer cell proliferation and metastasis in neuroblastoma and gallbladder cancer [82, 83]. TGF-β1 increases the mRNA expression of HK2 in glioblastoma cells [84], indicating that TGF-β enhances glycolysis partially by upregulating HK2. The enzyme 6-phosphofructo-2-kinase/fructose-2,6-bisphosphatase 3 (PFKFB3) is responsible for the synthesis of fructose-2,6-bisphosphate, an allosteric activator of the glycolytic enzyme 6-phosphofructo-1-kinase (PFK1) [85, 86]. TGF-β1 elevates PFKFB3 expression and enhances glycolysis in Panc1 pancreatic carcinoma cells. PFKFB3 silencing inhibits TGF-β-induced invasion in this human Panc1 cell line by repressing SNAIL expression [87]. This study suggested that the enzyme PFKFB3 is a promoter of TGF-β-induced EMT. Moreover, PFKFB3 is also elevated by TGF-β1 in human glioma cells, increasing fructose-2,6-bisphosphate, glucose uptake, glycolytic flux, and lactate production [84]. These results revealed that TGF-β-induced PFKFB3 overexpression is responsible for upregulating the Warburg effect by increasing the glycolytic enzyme PFK1. PFKFB3 serves as a “crossroad” connecting the Warburg effect and EMT.

Pyruvate kinase M2 (PKM2) is frequently overexpressed in human cancers and contributes to tumorigenesis [88]. This enzyme participates in the second to last step of glycolysis, during which one phosphoenolpyruvate (PEP) is dephosphorylated to pyruvate with the production of 2 ATPs. In colon cancer cells, PKM2 interacts with TGF-β-induced factor homeobox 2 (TGIF2) during TGF-β-induced EMT. TGIF2 is a TGF-β signaling transcriptional repressor, and the complex between PKM2 and TGIF2 promotes histone H3K9 deacetylation, resulting in a decrease in E-cadherin transcription, which contributes to metastasis by inducing EMT of cancer cells [89]. Consistently, in lung cancer A549 cells, TGF-β induces the overexpression of PKM2 by TGIF2 during EMT, and decreasing PKM2 results in the downregulation of EMT [90]. These data suggest that the TGIF2 is the mediator between TGF-β and PKM2, and this “TGF-β-TGIF2/PKM2” positive regulation network strengthens EMT and demonstrates a connection between glycolysis enzymes and EMT. Many small-molecule inhibitors and hormones can inhibit cell proliferation by targeting PKM2 [91, 92]. Inhibitors, namely shikonin and its analogs lapachol, lead to reduced glycolysis and increased necroptosis and apoptosis in human breast cancer cells and melanoma cells [92, 93], supporting PKM2 as a potential TGF-dependent glycolysis target for cancer therapy.

The results shown above reveal that TGF-β stimulates glycolysis. Conversely, glycolysis-induced acidosis also enhances TGF-β1-mediated EMT. Extracellular lactate induces SNAIL1 and EMT by directly remodeling the extracellular matrix and releasing activated TGF-β1 in human lung adenocarcinoma cells [94]. Furthermore, high extracellular lactate levels contribute to immune evasion, thereby promoting tumor growth and metastasis [95]. This study suggested that “TGF-β1-Warburg effect-lactate-TGF-β1” forms a positive regulation loop that constitutes an TGF-β-dependent acidic and immunosuppressive TME.

In addition to glycolysis, the TGF-β pathway is also related to the TCA cycle and the PPP pathway in cancer cells. It has been reported that succinate dehydrogenase B subunit (SDHB) knockdown contributes to colorectal cancer cell invasion and migration via EMT by activating the TGF-β signaling pathway through SNAIL1-SMAD3/SMAD4 [96]. Similarly, other researchers have observed that the knockdown of SDHB results in a hypermethylated epigenome, which can induce EMT in mouse ovarian cancer cells [97]. These studies demonstrated that the TCA cycle changes lead to TGF-β signaling-induced EMT via changes in enzymes such as SDHB. However, research concerning the role of TGF-β in regulating the TCA cycle remains to be explored. Inhibition of the TGF-β pathway through knockdown of TGF-βRI in hepatocellular carcinoma SNU449 cells correlates with reduced expression of PPP-related genes, including G6PD, hexose-6-phosphate dehydrogenase, and 6-phosphogluconolactonase [98]. This study indicated a role for TGF-β signaling in shunting glucose into the PPP pathway, which provides precursors for lipid and nucleotide synthesis. Moreover, TGF-β signaling is responsible for cisplatin resistance [99, 100]. The “TGF-β1-FOXM1-HMGA1-TGF-β1” positive feedback loop plays a crucial role in cisplatin-resistant NSCLC by upregulating the expression of G6PD, a critical enzyme of the PPP, while interrupting the “FOXM1-HMGA1-G6PD” pathway can sensitize the cells to cisplatin, providing a potential therapeutic target to strengthen chemosensitivity in cisplatin-resistant NSCLC  [101]. These studies indicated that TGF-β-induced cisplatin resistance is partially mediated by encouraging PPP, which provides precursors for nucleotide synthesis.

Glucose can be utilized not only for glycolysis but also for glycogenesis. LEFTY2 (endometrial bleeding-associated factor) is a cytokine that is released shortly before menstrual bleeding. LEFTY2 upregulates the expression and activity of the glucose transporters sodium-dependent glucose transporter 1 (SGLT1) and GYS1 in Ishikawa and HEC1a cells (two human endometrial cancer cell lines). It facilitates cellular glucose uptake and glycogenesis, although TGF-β1 can diminish this effect in endometrial cancer cells [102], demonstrating that TGF-β1 negates glycogen synthesis. Glycogen synthase kinase (GSK-3) is a serine/threonine kinase that deactivates the glycogen synthase enzyme and obstructs glycogen synthesis. In humans, there are two GSK isoforms, GSK-3α and GSK-3β. GSK-3β can negatively modulate TGF-β/SMAD3 signaling by facilitating SMAD3 deactivation after SMAD3 phosphorylation in HepG2 hepatocellular carcinoma cells [103]. These results reveal a negative regulation of TGF-β signaling by GSK-3 through inhibiting SMAD3 activity. Conversely, GSK-3 can be regulated by TGF-β signaling in hepatocellular carcinoma. TGF-β inactivates GSK-3β, which hampers activation of the tumor suppressor hepatocyte nuclear factor 4 alpha, a transcription factor that downregulates the expression of EMT master genes, including SNAIL1 [104]. In this way, TGF-β signaling upregulates SNAIL and finally contributes to EMT by GSK-3β inactivation, which may promote glycogen synthesis. A similar TGF-β-mediated LEFTY/AKT/GSK-3 inactivation/SNAIL axis that promotes ovarian clear cell EMT was recently observed [105]. These data reveal that TGF-β signaling may have a dual role in glycogen synthesis in different cancer types. GSK-3 can negatively regulate or be regulated by TGF-β signaling. Upregulation of GSK-3 could be a potential cancer therapy by preventing TGF-β-induced EMT in certain types of cancer.

### Lipid metabolism

#### Lipid metabolism phenotypes of cancer cells

Lipids are various organic compounds that are insoluble in water. They include cholesterol, phospholipids, sphingolipids, and triglycerides. Fatty acids are the main building blocks of lipids and can connect with various metabolic pathways to synthesize complex lipids. Lipid metabolism reprogramming in cancer cells was commonly disregarded in the past but has received increasing attention in recent years. Studies have demonstrated that lipid metabolism reprogramming plays an important role in providing energy, biomolecules for membrane synthesis, and lipid signals during cancer progression [34].

Cholesterol is not only an important part of the cell membrane but also an energy source for cells [106]. The distribution and abundance of cholesterol are closely correlated with membrane fluidity and cancer cell biological behaviors. Zhao et al. observed that the cholesterol efflux channel ATP-binding cassette transporter A1 potentiates breast cancer cell metastasis in vitro and in vivo by decreasing membrane cholesterol abundance, which increases cell membrane fluidity and EMT [107]. Furthermore, the authors found that ATP-binding cassette A1 is overexpressed in 41% of metastatic tumors [107], revealing that cholesterol negatively regulates cell membrane fluidity and the consequent metastatic activity of cancer cells. However, other researchers have observed that positive regulation may exist between cholesterol and cancer cell metastasis. Baek et al. [108] found that 27-hydroxycholesterol, a metabolite derived directly from cholesterol, promotes lung metastasis of breast cancer by its action on myeloid cells in distal metastatic sites, thus promoting an immunosuppressive environment. These data indicate that cholesterol may enhance cancer cell metastasis by metabolite-initiated signals. Collectively, cholesterol plays a paradoxical role in cancer cell metastasis, and targeting cholesterol metabolism should be re-evaluated in each cancer type.

Fatty acids are required for bulk tumor growth. Cancer cells acquire more fatty acids by increasing de novo fatty acid synthesis, lipid uptake, and lipolysis, thus sustaining their rapid proliferative rate and providing an essential energy source [109], and even protecting cells from apoptosis while regulating cancer migration and invasion [110]. In addition, as cancer cells tend to store glycogen, they have more lipid droplets that are representative of lipid storage than normal cells [111]. In the hypoxic TME, a fatty acid uptake protein, fatty acid-binding protein 7, is upregulated in breast cancer cells, which results in lipid droplet formation [112]. Fatty acid synthase (FASN) upregulation is another mechanism that induces fatty acid accumulation in cancer cells [113, 114]. Fatty acid oxidation (FAO) involves the breakdown of fatty acids into acetyl-CoA units. Aiderus et al. [115] reported that FAO is downregulated in breast, colorectal, prostate, and head and neck cancer, among others. However, Mozolewska et al. [116] suggested that FAO is accelerated in colorectal cancer, and targeting FAO is a potential treatment, suggesting that FAO alterations are context- or cancer-type-dependent. Overall, fatty acid acquisition, including FA synthesis, lipid uptake, and lipolysis, increases in cancer cells and is a promising therapeutic strategy for human cancer. However, FAO is not a potential cancer target because of its bidirectional roles in cancer development.

#### TGF-β signaling in lipid metabolism of cancer cells

Cholesterol is a major cell membrane component of lipid rafts/caveolae and is closely related to TGF-β signaling responses. NADPH steroid dehydrogenase-like protein (NSDHL) is an enzyme involved in cholesterol biosynthesis. NSDHL is highly expressed in human breast cancer tissues and predicts a poor prognosis. NSDHL knockdown suppresses breast cancer cell proliferation and migration via TGF-βRII endosomal degradation [117]. Thus, NSDHL promotes breast cancer proliferation and metastasis through inhibition of TGF-βRII degradation, indicating that cholesterol upregulates TGF-βRII and subsequent TGF-β signaling. However, other researchers have reported contradicting results between cholesterol synthesis and TGF-β signaling. Cholesterol is unevenly distributed on the cell membrane and is dynamically exchanged between the cytoplasm and the membrane through endosome formation. TGF-β receptors are located in cholesterol-enriched subdomains, and cholesterol-mediated TGF-β receptor endocytosis and subsequent degradation are known as important repressive mechanisms of the TGF-β signaling pathway [118–120], demonstrating that cholesterol downregulates TGF-β receptors. Similarly, in PDAC, cholesterol biosynthesis interruption by NSDHL inactivation or treatment with cholesterol-lowering statin drugs induces the transformation of glandular pancreatic carcinomas to a mesenchymal phenotype via TGF-β1 overexpression in mouse models [121]. These data suggest that NSDHL or cholesterol synthesis downregulates TGF-β1 production and inhibits EMT. Moreover, cholesterol can downregulate TGF-β signaling responses by decreasing the TGF-βRII/TGF-βRI-binding ratio of TGF-β on the cell surface [122]. These data reveal that cholesterol downregulates TGF-β signaling in cancer cells. Taken together, cholesterol biosynthesis contradictorily influences TGF-β signaling by regulating TGF-βRII and TGF-β1 expression in different cancers, which explains the paradoxical role of cholesterol in cancer cell metastasis. In addition, TGF-β also regulates cholesterol synthesis as an upstream component. Zhao et al. [123] observed that TGF-β treatment of MDA-MB-231 human breast cancer cells decreases the amount of cholesterol, while a TGF-β inhibitor increases it. Sterol regulatory element-binding transcription factor 2 (SREBF2) is a major regulator of cholesterol synthesis. TGF-β-induced EMT-related transcription factor ZEB1 decreases cholesterol in breast cancer cells by forming a complex with C-terminal-binding protein, and the ZEB1-C-terminal-binding protein complex binds to the SREBF2 promoter and inhibits its activity [124]. This study suggested that cholesterol downregulation is a common consequence of TGF-β-induced EMT, and TGF-β signaling decreases cholesterol synthesis by increasing EMT transcription factors. Based on the above results, cholesterol can function as an upstream and downstream component of TGF-β signaling and has a paradoxical role in cancer growth and metastasis.

Fatty acid synthesis is commonly upregulated in cancer cells. FASN is a multifunctional and central lipid biosynthesis enzyme that is responsible for fatty acid formation from acetyl-CoA, malonyl-CoA, and NADPH [125]. FASN is related to TGF-β signaling. Cisplatin-resistant NSCLC A549CisR and H157CisR cell lines harbor slight growth retardation but exhibit higher EMT and increased metastatic potential. These cisplatin-resistant cells show an upregulation of FASN and TGF-β1, and FASN inhibition results in a slight growth reduction and a significant reduction in TGF-β1, thus decreasing the EMT/metastatic potential of cisplatin-resistant cells. Intriguingly, TGF-β inhibitor SB525334 treatment downregulates and TGF-β1 stimulation upregulates FASN levels [126]. These results indicate the presence of a “FASN-TGF-β1-FASN” positive loop in cisplatin-resistant cancer cells. Consistent with TGF-βRI knockdown in SNU449 hepatocellular carcinoma cells, decreased levels of sphingolipids and phospholipids have been detected together with decreased expression of fatty acid synthesis genes, such as acyl CoA synthetase 5 (ACSL5) and peroxisome proliferator-activated receptor gamma (PPARγ) [98]. These data indicate that TGF-β signaling increases fatty acid synthesis by upregulating fatty acid synthesis-related proteins, including FASN, ACSL5, and PPARγ. Conversely, TGF-β1 induces EMT and activates p-AMPK in MCF-7 breast cancer cells. In this setting, p-AMPK increases FAO accompanied by decreased FASN and augments fatty acid β‑oxidation enzymes, such as carnitine palmityl transferase 1 and CD36, in MCF‑7 breast cancer cells during EMT [127]. This study implies a role of the non-canonical TGF-β signaling pathway and negative TGF-β signaling regulation in fatty acid synthesis. Taken together, TGF-β signaling may play a paradoxical role in fatty acid synthesis via the bidirectional regulation of FASN expression (Table 2).

**Table 2:** TGF-β-dependent metabolic reprogramming of lipid and amino acid in cancer

Signaling components	TGF-β-dependent metabolic component change	Metabolic reprogramming/cell biology influenced	Cell Type	Cancer type	Experimental status	Ref.
Lipid						
*Cholesterol synthesis*						
NSDHL-TGF-βR2	NSDHL promoted TGF-βR2 activation	Promoted cholesterol biosynthesis. Facilitated breast cancer cell proliferation and metastasis	Cancer cell	BC	In vitro human cell culture; Preclinical in vivo mouse model	[117]
NSDHL-SREBP1-TGF-β1	NSDHL inhibited TGF-β1 production	Promoted cholesterol biosynthesis; Inhibited EMT	Cancer cell	PDAC	In vitro mouse cell culture; Preclinical in vivo mouse model	[121]
TGF-β-ZEB1/CtBP complex-SREBF2-TGF-βRI	ZEB1/CtBP complex Inhibited the activity of SREBF2 via bounding to its promoter	Decreased cholesterol synthesis; Increased EMT and metastasis	Cancer cell	BC	In vitro mouse cell culture; Preclinical in vivo mouse model	[123]
CAV-1-AKT-TGF-β1	Downregulated CAV-1 in CAFs increased TGF-β1 through AKT activation	Increased levels of intracellular cholesterol and high metastatic behavior in CAV-1-depleted CAFs	CAF	Prostate cancer	In vitro human cell culture	[176]
*Fatty acid synthesis*						
TGF-β1-FASN-TGF-β1	“FASN-TGF-β1-FASN” positive regulatory loop	Increased fatty acid synthesis; Increased EMT/metastasis	Cancer cell	NSCLC	In vitro human cell culture	[126]
TGF-β1-ACSL5 and PPARγ	Increased ACSL5 and PPARγ	Reduced mitochondrial respiration; Increased EMT	Cancer cell	HCC	In vitro human cell culture	[98]
TGF-β1-p-AMPK-FASN	Activated p-AMPK and thus decreased FASN	Decreased fatty acid synthesis; Increased EMT	Cancer cell	BC	In vitro human cell culture	[127]
*Endocytosis and lipid droplet formation*						
Acidic TMME-TGF-β2 releasement-CD36	Acidosis increased TGF-β2 releasement and then CD36	Increased fatty acid uptake and formation of lipid droplet; Enhanced anoikis resistance and cancer cell invasiveness	Cancer cell	Uterus and colon cancer	In vitro human cell culture	[131]
*Fatty acid oxidation*						
TGF-β1-p-AMPK-CPT1 and CD36	Activated p-AMPK and thus increased CPT1 and CD36	Enhanced fatty acid oxidation pathway; Increased EMT	Cancer cell	BC	In vitro human cell culture	[127]
TGF-β-TGF-βRI	TGF-βRI was observed to be upregulated	Increased β-oxidation of long-chain fatty acids. Promoted TGF-β-induced EMT	Cancer cell	HCC	In vitro human cell culture	[270]
Amino acid						
TGF-β-P4HA3	Induced the expression of P4HA3	Increased the levels of Asp, Glu, and Lys	Cancer cell	NSCLC	In vitro human cell culture; Preclinical in vivo mouse model	[137]
TGF-β-SLC7A5 and GLS1	upregulated Gln transporter SLC7A5 and GLS1	Enhanced Gln anaplerosis	Cancer cell	HCC	In vitro human cell culture	[98]

*NSDHL* NAD(P)H steroid dehydrogenase-like protein; *SREBF2* sterol regulatory element-binding transcription factor 2; *ZEB1* zinc finger E-box-binding homeobox 1; *CtBP* C-terminal-binding protein; *CAV-1* caveolin-1; *CAFs* cancer associated fibroblasts; *FASN* fatty acid synthase; *ACSL5* acyl CoA synthetase 5; *PPARγ* peroxisome proliferator-activated receptor gamma; *p-AMPK* phosphorylated AMP-activated protein kinase; *ERK* extracellular signal-regulated kinase; *LDs* lipid droplets; *CPT1* carnitine palmityl transferase 1; *P4HA3* prolyl 4-hydroxylase subunit alpha 3; *SLC7A5* solute carrier family 7 member 5; *BC* breast cancer; *PDAC* pancreatic ductal adenocarcinoma; *NSCLC* non-small cell lung cancer; and *HCC* hepatocellular carcinoma

In addition to cholesterol metabolism and FASN-mediated fatty acid synthesis, lipid droplets also depend on TGF-β signaling in cancer cells. The acidic TMME induces the formation of lipid droplets [128, 129], which are the storage organelles at the center of lipid and energy homeostasis [130]. Acidosis promotes autocrine TGF-β2 in human uterus and colon cancer cells, and TGF-β signaling activation facilitates the FA uptake and formation of lipid droplets that act as an energy store, and it readily supports anoikis resistance and cancer cell invasiveness. TGF-β2 activation promotes both EMT and FAO by increasing the acetyl-CoA pool, and the latter enhances SMAD2 activity [131]. These results demonstrate that canonical TGF-β signaling is involved in LD formation. Since fatty acids are a basic component of lipid droplets, an increase in lipid droplet formation partially explains why fatty acid synthesis is upregulated in cancer cells, i.e., for the storage of energy sources for cancer cells in the nutrient-deprived TMME.

### Amino acid metabolism

Proteins in the human body should first be broken down into amino acids, and amino acid metabolism should then be representative of the core metabolism after protein absorption. Amino acid metabolism has extremely extensive effects in cancer cells, including (1) the generation of amino acids as building blocks and their conversion to glucose, lipids, and precursors for nucleic acid synthesis; (2) the supply of bioenergy through producing α-ketoacid, which can be oxidized by the TCA cycle and undergo oxidative phosphorylation for ATP production; (3) the generation of nutrient signals to activate cancer-related pathways; and (4) maintenance of the intracellular redox status [132, 133]. Abnormal amino acid metabolism has been reported, and its potential impact on TMME is becoming increasingly important.

#### Amino acid phenotypes of cancer cells

Cancer cells have increased amino acid requirements to meet their rapid proliferation demand. Amino acids consist of two classes: nonessential amino acids, including glutamate (Glu), glutamine (Gln), serine (Ser), glycine (Gly), and proline (Pro); essential amino acids, such as arginine (Arg), leucine (Leu), and methionine (Met) [134]. Increased Gln metabolism is a common metabolic reprogramming that occurs in cancer. Glutaminolysis can be engaged in cancer cells when the glucose supply is deficient. Gln is first converted into glutamate, which is metabolized to alpha-ketoglutarate (α-KG) in mitochondria, an intermediate metabolite used in the TCA cycle for OXPHOS-driven energy production. Ser and Gly are linked in the biosynthesis of proteins, nucleic acids, and lipids that are crucial to cancer proliferation [134]. Proline is a unique proteinogenic secondary amino acid and a basic component of collagen, and proline metabolism is involved in the aggressive phenotype of cancer [135]. Arg is an essential amino acid, and many types of cancer cells die rapidly in culture medium deprived of Arg [136].These studies indicate that cancer cells show a different appetite for amino acids, which may be linked with a higher demand for biomacromolecules for cancer cell proliferation and immune evasion. Further studies should be conducted to extend our knowledge on how these amino acid changes contribute to cancer development and to help modulate cancer patients’ diets in cases of developing cancer or cancer progression.

#### TGF-β signaling in amino acid metabolism of cancer cells

TGF-β-dependent reprogramming of amino acid metabolism also correlated with EMT. In human lung adenocarcinoma A549 cells, TGF-β treatment-induced EMT, increased the levels of aspartic acid (Asp), Glu, and lysine (Lys), whereas decreased the levels of alanine, asparagine, citrulline), Gln, Gly, histidine, hydroxyproline, isoleucine, Leu, phenylalanine, Pro, threonine, and tyrosine (Tyr). To mimic the amino acid changes elicited by TGF-β, A549 cells were cultured in media depleted of Ala, Asn, Gly, His, hydroxyproline, Ile, Leu, Met, Phe, Pro, Thr, Trp, Tyr, and valine (Val). Treatment with media depleted of amino acids induced EMT-like responses similar to TGF-β-induced EMT [137]. These results suggested that specific amino acid depletion is sufficient to induce EMT, and amino acid metabolism plays an essential role during EMT. Except for the regulation of these EMT genes, amino acids are also responsible for the cell shape. Depletion of Phe, Thr, tryptophan (Trp), Lys, Val, Met, Leu, Ile, Gln, Arg, or Tyr, but not His, significantly induced morphological changes from an epithelial pebble-like shape to an elongated mesenchymal shape in A549 cells [137]. Prolyl 4-hydroxylase subunit alpha 3 (P4HA3), a key enzyme in collagen synthesis, was upregulated and involved in the alteration of amino acid metabolism in TGF-β-stimulated cells. P4HA3 knockdown abrogated TGF-β-induced amino acid changes and EMT [137], highlighting that the key collagen synthesis enzyme P4HA3 is a critical component that mediates TGF-β-induced amino acid metabolism reprogramming and a potential EMT target. These data demonstrates that amino acid changes induced by TGF-β contributed to cancer cell EMT, and collagen synthesis is relative with this process.

Additionally, elevated Gln metabolism after TGF-β treatment has been verified. In hepatocellular carcinoma, TGF-β upregulates Gln transporter solute carrier family 7 member 5 and glutaminase 1, which induces enhanced Gln anaplerosis [98]. In this way, TGF-β increases the absorption of glutamate extracellularly and pushes Gln metabolites into the TCA cycle. Increased Gln addiction provides resistance to metabolic stress through energy production. These results strongly suggest a role of TGF-β signaling in promoting Gln metabolism and thereby increasing the survival of cancer cells (Table 2).

### Other TGF-β-dependent metabolism in cancer cells

Reactive oxygen species (ROS) are mainly produced in mitochondria by energy metabolism and play an important role in balancing the cellular redox state. ROS also serve as signaling molecules to regulate cancer biological processes, such as TGF-β-induced EMT. ROS levels in cancer are higher than those in normal tissue, and ROS affect many aspects of tumorigenesis. ROS production is suggested to be induced by TGF-β and to mediate cell proliferation, apoptosis, and EMT. The mitochondrial enzyme superoxide dismutase 2, which catalyzes O_2_^•−^ radicals to H_2_O_2_ and oxygen, is upregulated upon TGF-β treatment in human oral and esophageal epithelial cell lines [138]. In addition, TGF-β also elicits NADPH oxidase 4 to produce O_2_^•−^ followed by dismutation into H_2_O_2_, and these H_2_O_2_ molecules inhibit protein tyrosine phosphatase 1B, a negative regulator of EMT. Additionally, NADPH oxidase 4-derived H_2_O_2_ stimulates TGF-β-induced p38-MAPK activation, which enhances EMT by elevating SNAIL1 expression [27]. Altogether, TGF-β induces ROS production and ROS mainly facilitates EMT. Nitric oxide (NO) is another important redox and a cytotoxic molecule that was previously believed to be a mediator of macrophage cytotoxicity [139]. Interestingly, researchers found that cancer cells also produce NO [140–142]. TGF-β1 significantly downregulates NO synthesis in colon carcinoma cells via an intracellular mechanism [142], suggesting that targeting TGF-β1 could serve as a cancer therapy by upregulating NO production. In conclusion, ROS may have a dual role in cancer progression depending on the specified ROS species.

**Fig. 3:**
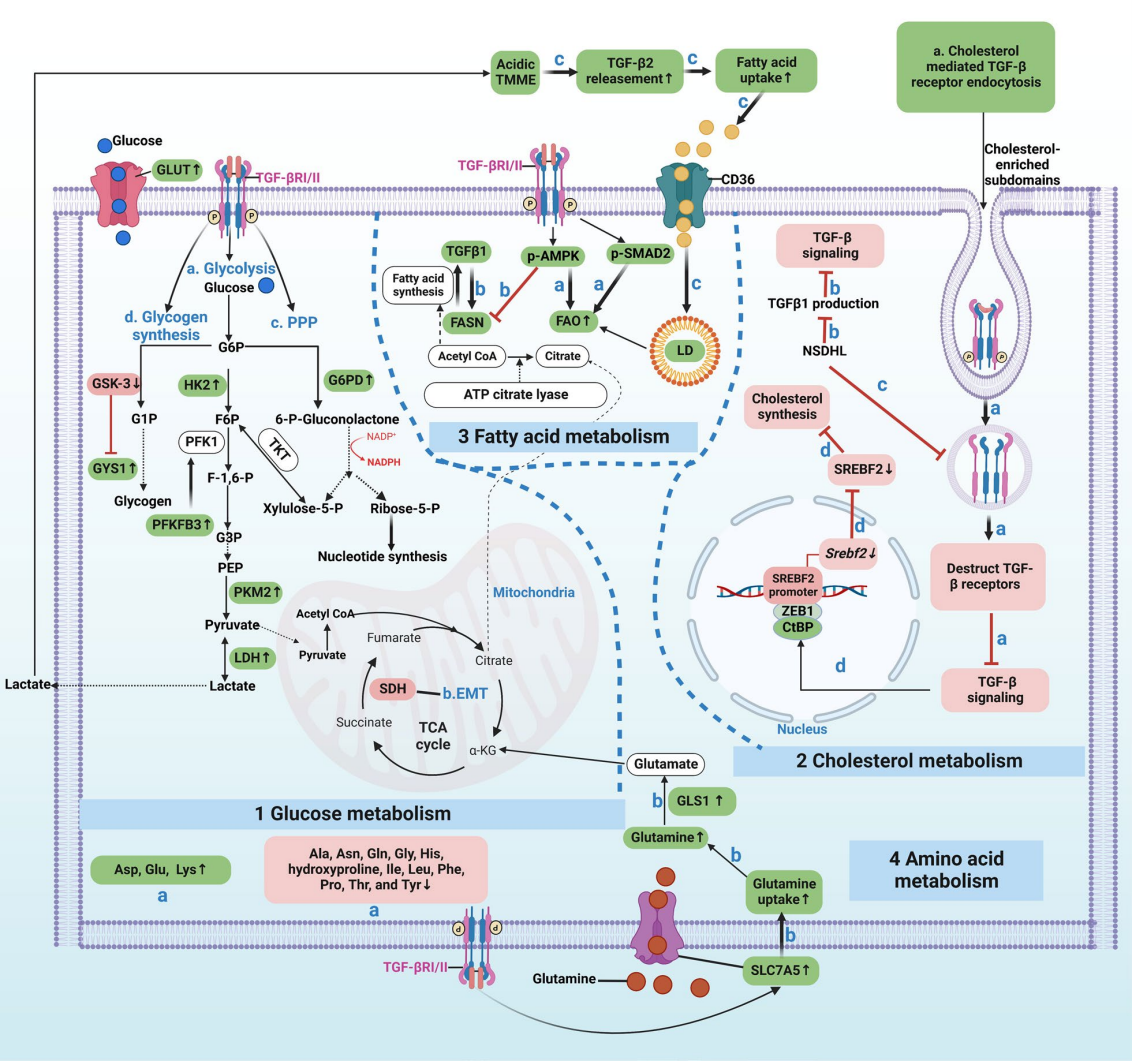
TGF-β-dependent metabolism reprogramming of cancer cells. 1. For glucose metabolism, TGF-β signaling can: **a**. enhance glycolysis by directly increasing the glycolytic enzyme expression of PKM2 and LDH, and indirectly elevate the PFKFB3, which augments the expression of PFK1, one of the glycolytic enzymes; **b**. TCA cycle enzyme SDH mutation leads to TGF-β-induced EMT; **c**. augment PPP by increasing G6PD, the first key enzyme of PPP; and **d**. promote glycogen synthesis by boosting GYS1 expression via inhibiting GSK-3 that deactivates GYS1. 2. For cholesterol metabolism, **a**. cholesterol-enriched subdomain-mediated TGF-β receptor endocytosis can: destruct TGF-β receptors that abrogate TGF-β signaling initiation; **b**. cholesterol synthesis enzyme NSDHL can inhibit TGF-β1 production and attenuate TGF-β signaling; **c**. NSDHL also prevents TGF-βRII endocytosis and then facilitates TGF-β signaling; and **d**. TGF-β signaling can decrease cholesterol synthesis by inactivating SREBF2, a cholesterol synthesis promoter. 3. For fatty acid metabolism, **a**. canonical (p-SMAD2) and non-canonical (p-AMPK) TGF-β signaling accelerates FAO; **b**. TGF-β1 can increase FASN expression, thus allowing fatty acid synthesis and accumulation in cancer cells; conversely, FASN enhances TGF-β1 production. Moreover, non-canonical (p-AMPK) signaling decreases FASN; **c**. TGF-β2 releasement by the acidic TMME enables more fatty acid entry into cancer cells and forms LD for lipid storage used by FAO. 4. For amino acid metabolism, TGF-β signaling can: **a**. increase Asp, Glu, and Lys, while decrease Ala, Asn, Gln, etc.; **b**. bolster glutamine’s entry into cancer cells by elevating SLC7A5, and enhance glutamine’s catalyzation into glutamate via increasing GLS1, and glutamate can flow into TCA cycle. Green highlighted items mean TGF-β signaling positively regulates them, or they are positively regulated by TGF-β signaling. Red vice versa. And these green and red ones are potentially TGF-β-dependent metabolic targets in cancer. HK2: Hexokinase 2; G6P: glucose 6-phosphate; F6P: fructose 6-phosphate; PFK1: phosphofructokinase 1; PFKFB3: 6-phosphofructo-2-kinase/fructose-2,6-biphosphatase 3; PPP: pentose phosphate pathway; Acetyl-CoA: acetyl coenzyme A; GYS1: glycogen synthase 1; GSK-3: glycogen synthase kinase 3; F-1,6-P: fructose-1,6-bisphosphate; G3P: glyceraldehyde 3-phosphate; PEP: phosphoenolpyruvate; PKM2: pyruvate kinase M2; LDH: Lactate dehydrogenase; SREBF2: sterol regulatory element-binding transcription factor 2; FASN: fatty acid synthase; FAO: fatty acid oxidation; LD: lipid droplet. ASP: aspartic acid; Glu: glutamic acid; Lys: lysine; Ala: alanine; Asn: asparagine; Gln: glutamine; Gly: glycine; His: histidine; Ile: isoleucine; Leu: leucine; Phe: phenylalanine; Pro: proline; Thr: threonine; Tyr: tyrosine. SLC7A5: glutamine transporter solute carrier family 7 member 5; and GLS1: glutaminase 1

Overall, for glucose metabolism, TGF-β signaling enhances glycolysis and PPP by upregulating related enzymes. In this way, TGF-β signaling increases the production of macromolecule precursors to sustain cancer cell survival and activity. In addition, TCA enzyme mutation in cancer can activate TGF-β signaling-induced EMT. Moreover, TGF-β signaling downregulates or facilitates glycogen synthesis during EMT in different contexts. Regardless, targeting TGF-β-dependent glycolysis and PPP by its mediated enzymes could benefit cancer therapy by obstructing biomass precursor synthesis, EMT and chemotherapy resistance. For lipid metabolism, cholesterol and fatty acid metabolism are influenced by TGF-β signaling. They can be upstream and downstream components of TGF-β signaling. As for amino acid metabolism, TGF-β signaling can increase Asp, Glu, and Lys while decreasing Ala, Asn, and Gln, among others. Additionally, under TGF-β stimulation, Gln entry into cancer cells is elevated by SLC7A5 upregulation. Furthermore, TGF-β signaling enhances Gln catalysis into glutamate by increasing GLS1, and glutamate can flow into the TCA cycle (Fig. 3).

## TGF-β-dependent metabolism of stromal–epithelial coupling and targeted therapies

CAFs and immune cells reprogram their metabolism mainly for cancer cell support. The cellular metabolism of stromal cells closely interacts with cancer cell metabolism or biological behavior and vice versa. These interactions are called coupling, such as CAFs–epithelium and epithelial–immunometabolic coupling. CAFs can be derived from several sources, including resident normal fibroblasts (NFs), mesenchymal stem cells, and EMT [143]. TGF-β1 treatment induces NFs to become CAFs in various cancers, including breast, bladder, colorectal, and pancreatic cancer [144–146]. Elevated p-SMAD2 and p-SMAD3 were found during this process, implying that canonical TGF-β signaling is active in this process [145]. Furthermore, TGF-β1 alters the epigenetic signature of fibroblasts, resulting in differential gene expressions, such as α-SMA and FAP, and stronger collagen synthesis in CAFs [147]. TGF-β signaling is also related to the immunosuppressive features of immune cells. Overall, TGF-β signaling is closely correlated with CAFs and immune cell behaviors, which are derived by cellular metabolism. This section will illustrate the role of TGF-β signaling in stromal cell metabolism reprogramming and stromal–epithelial metabolism coupling.

### CAFs–epithelia metabolism coupling

#### Metabolic phenotypes of CAFs

CAFs are the most abundant stromal cells that promote cancer growth and metastasis [10, 148, 149]. Glucose metabolism reprogramming in CAFs is mainly involved in glycolysis and the TCA cycle. Glycolysis is enhanced in CAFs, as glycolytic enzymes including HK2 and 6-phosphofructokinase liver type, are significantly upregulated in CAFs [150–152]. Zhang et al. [150] identified that the TCA cycle enzyme isocitrate dehydrogenase 3α (IDH3α) is decreased in CAFs. In primary fibroblasts with IDH3α knockdown, glucose uptake and lactate production are increased, whereas oxygen consumption is decreased. Therefore, the downregulation of the TCA cycle enzyme IDH3α is responsible for the enhanced aerobic glycolysis in CAFs, revealing a negative relationship between glycolysis and the TCA cycle. IDH3*α* downregulation decreases *α*-KG production, which inhibits the activity of prolyl hydroxylase domain-containing protein 2, a HIF-1 downregulator; its inhibition enables HIF-1*α* protein stabilization in the cytosol [153, 154]. HIF-1α has been reported to be associated with the upregulation of the glycolytic pathway [155]. Hence, HIF-1α promotes glycolysis by increasing glucose uptake and OXPHOS inhibition by upregulating NADH dehydrogenase ubiquinone 1 alpha subcomplex, 4-like 2 (NDUFA4L2), a negative regulator of mitochondrial complex 1 [150]. Hence, IDH3α downregulation increases glycolysis via HIF-1α. Taken together, glycolytic enzyme upregulation and IDH3α downregulation promote glycolysis and inhibit OXPHOS, shedding light on the initiation of aerobic glycolysis in CAFs.

Altered lipid metabolism in CAFs has received increasing concern in recent years. Similar to cancer cells, CAFs in colorectal cancer undergo lipid metabolism, which symbolizes more fatty acid accumulation resulting from CAF FASN upregulation. Fatty acids are secreted extracellularly and are taken up by colorectal cancer cells to increase their migration. CAF-induced colorectal cancer cell migration is abolished by FASN knockdown or by reducing the uptake of fatty acids in vitro and in vivo [159]. These data suggest that fatty acids secreted from CAFs contribute to colorectal cancer cell migration, provide new insight into the mechanism of CRC metastasis, and suggest that FASN could be a potential target for anti-CRC metastasis treatment in the future. Since FASN is elevated in both cancer cells and CAFs, FASN could be a potential epithelial–stromal common target proposed in our previous study [160]. Similarly, in PDAC, intracellular levels of lysophospholipids, another type of lipid, increase dramatically in activated stroma-associated pancreatic stellate cells, a CAF-like cell type in pancreatic ductal adenocarcinoma, and some of them are secreted into the TME, from which some are directly absorbed and utilized by PDAC cells for membrane lipid formation [161]. Ketone bodies are intermediate products produced by fatty acid catabolism. CAFs generate more ketone bodies than NFs, and cancer cells reutilize these ketone bodies for OXPHOS in a similar manner to lactate to increase cancer cell proliferation [162]. Caveolin-1 (CAV-1) plays an important role in regulating lipid metabolism. Hu et al. [163] revealed that CAV-1 levels in tumor grafts are correlated with the expression levels of the enzymes that regulate lipolysis. TGF-β deficiency can increase stromal autophagy and the generation of ketone bodies. This research highlights that ketone bodies, as metabolites of fatty acids, are another energy source that can be transferred from CAFs to cancer cells. To conclude, CAFs exhibit higher catabolism to provide synthetic substrates and energy for cancer cell utilization.

Gln metabolism in CAFs promotes tumor growth. Yang et al. [164] found that CAFs have an upregulated Gln anabolic pathway compared with NFs by increasing Gln synthetase, and these Gln molecules maintain cancer cell growth when glucose is scarce. Cancer cells can absorb Gln from CAFs in a similar manner to lactate and ketone body transfer [163]. Gln is catalyzed in cancer cells by upregulated glutaminase and produces glutamate, which can enter the TCA cycle for ATP generation. In this way, ovarian cancer growth was accelerated in an ovarian cancer mouse model. Cotargeting of stromal Gln synthetase and cancer cell glutaminase disrupts this metabolic coupling, inducing tumor regression in this setting [164]. Mestre-Farrera et al. [165] observed that Gln deprivation promotes the migration and invasion of CAFs into the Gln-enriched environment, which, in turn, facilitates the movement of cancer cells toward nutrient-rich territories. These results demonstrated that CAFs are also addicted to Gln and shed light on the importance of Gln in CAF-mediated cancer cell movement. In summary, CAFs can directly “feed” cancer cell energetic metabolites, including lactate, ketone bodies, fatty acids, and amino acids, in a host–parasite pattern and finally contribute to tumor growth and metastasis.

#### TGF-β signaling in CAFs–epithelia coupling

The high rate of glycolysis in CAFs is believed to be one of the driving forces supporting tumor growth, which is called CAFs–epithelium glucose metabolism coupling and is defined as the “reverse Warburg effect (RWE).” “RWE” is a two-compartment tumor metabolism model in which catabolic CAFs undergo aerobic glycolysis and generate energy-rich metabolites, such as lactate and pyruvate, to feed mitochondrial OXPHOS in adjacent anabolic cancer cells [152, 166, 167]. Catabolic CAFs export lactates or pyruvates through MCT-4, and cancer cells can directly absorb these energy-rich metabolites through MCT-1 and then apply them to anabolism and proliferation [152]. This process enables cancer cells to live without blood vessels, as they can directly absorb energetic metabolites produced by CAFs, thus illustrating how cancer cells might survive during metastasis.

**Fig. 4:**
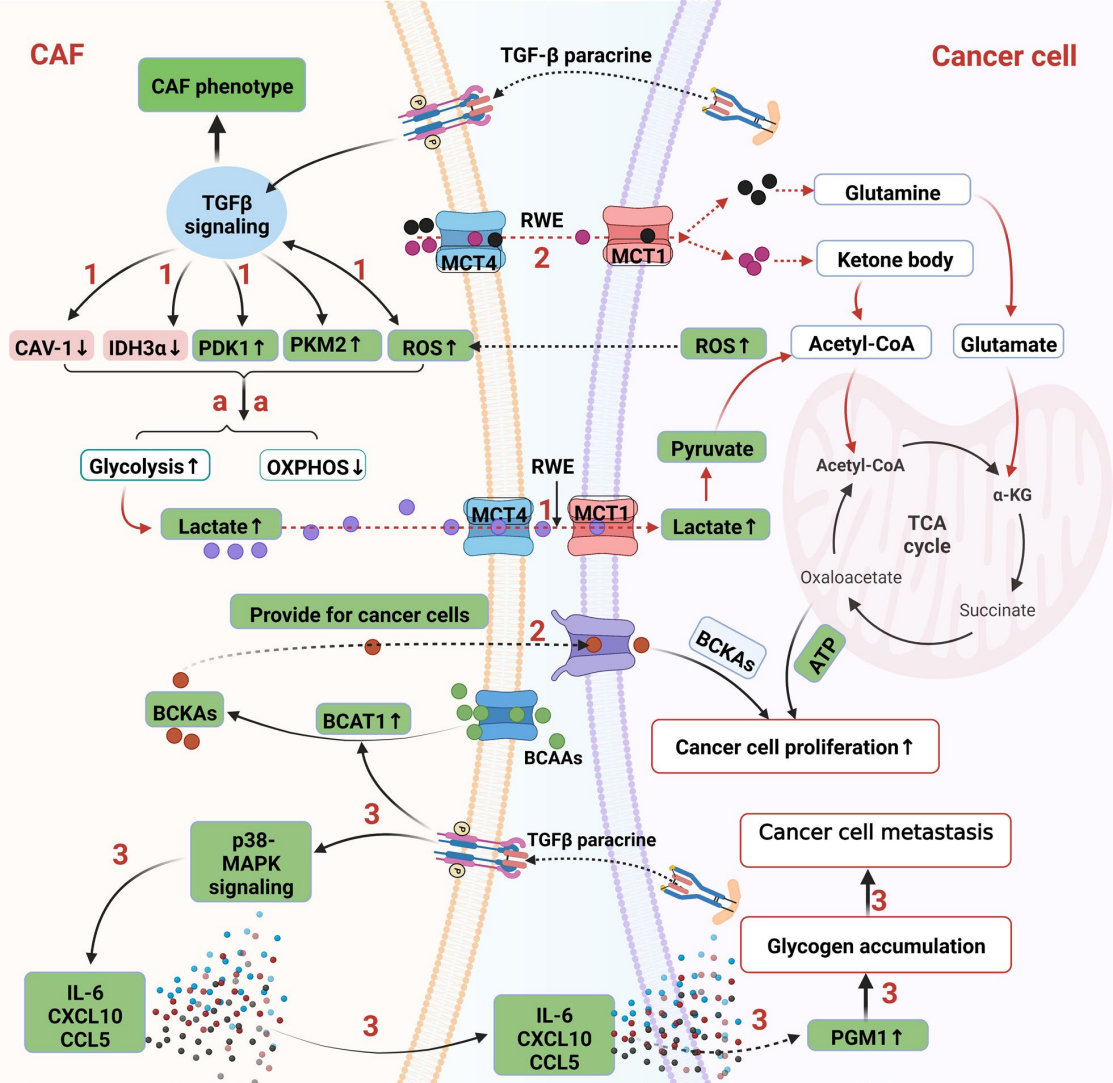
TGF-β-dependent CAF–cancer cell metabolic coupling. 1. TGF-β signaling increases RWE of CAFs via decreasing CAV-1 and IDH3α, while increasing PDK1, PKM2, and ROS. ROS conversely promotes TGF-β signaling, which sustains CAF phenotype. In this way, aerobic glycolysis of CAFs increases and a large amount of lactate molecules are produced. Lactate shuttles from CAFs to cancer cells via MCT-4 and MCT-1. Lactates are then converted to pyruvate and utilized for TCA cycle of cancer cell. 2. Glutamine, ketone body, and BCKAs are also substrates of RWE that are produced by CAFs and then are transferred into cancer cells for TCA cycle of cancer cell. 3. TGF-β-mediated metabolic coupling can also correlate with glycogen metabolism. Cancer cell-derived TGF-β cytokines trigger the TGF-β non-canonical p38-MAPK signaling in CAFs via paracrine, which stimulates the production of several cytokines including IL-6, CXCL10, and CCL5 from CAFs. These cytokines induce glycogen metabolism upregulation in cancer cells via phosphorylation and activation of PGM1, an enzyme that is involved in glycogen synthesis. Then glycogen is accumulated in cancer cells and promotes cancer cell metastasis. Green highlighted items mean TGF-β signaling positively regulates them or they are positively regulated by TGF-β signaling. Red vice versa. And these green and red ones are potentially TGF-β-dependent metabolic targets in cancer. PDK1: pyruvate dehydrogenase kinase 1; ROS: reactive oxygen species; RWE: reverse Warburg effect; BCAAs: branched-chain amino acids; BCKAs: branched-chain α-ketoacids. BCAT1: BCAA transaminases; and PGM1: phosphoglucomutase 1

TGF-β signaling is involved in RWE by regulating metabolic enzyme or molecules. Hu et al. [168] reported that the glycolytic enzyme HK2 is increased during the differentiation of CAFs induced by TGF-β1, indicating that TGF-β1 not only induces HK2 upregulation in the cancer cells mentioned above but also in CAFs. Fibroblasts can be activated by adjacent breast cancer cell-derived TGF-β in a paracrine fashion, leading to CAV-1 loss and subsequently enhanced oxidative stress, autophagy/mitophagy, and glycolysis in CAFs [16]. Furthermore, these CAF-secreted metabolites can spread among neighboring fibroblasts and sustain the growth of breast cancer cells [16]. These results indicate that TGF-β signaling promotes RWE through CAV-1 downregulation. CAV-1 downregulation inhibits TGF-βRII protein degradation and activates TGF-β signaling, supporting the “TGF-β1-CAV-1 downregulation-TGF-β activation” positive regulatory loop. IDH3α, as an enzyme of the TCA cycle, is another downstream target of TGF-β, and its downregulation contributes to RWE by increasing glycolysis and decreasing OXPHOS in fibroblasts, as discussed above [150]. In addition, TGF-β treatment decreases the flow of pyruvate to the TCA cycle by directly activating the gene encoding pyruvate dehydrogenase kinase 1 (PDK1), and this enzyme inactivates the TCA cycle enzyme pyruvate dehydrogenase in mouse and human fibroblasts, a human Burkitt’s lymphoma cell line, and human renal cell carcinoma cell lines [169, 170]. Our research team successfully separated and cultured human oral CAFs from human cancer [156], and verified the upregulated glycolysis via PFKFB3 and PKM2 overexpression in oral CAFs [157, 158]. However, we found that TGF-βRII was downregulated in oral CAFs and thus promoted PKM2 nuclear translocation via increasing p-ERK1/2, which was responsible for the elevated glycolysis in oral CAFs [157, 171]. This study demonstrates that TGFβRII is reversely correlated with glycolysis via activation of non-canonical TGF-β signaling. Therefore, TGF-β signaling regulates downstream of CAV-1, IDH3α, HIF-1α, PDK1, and PKM2, thus promoting CAFs–epithelia metabolic coupling, i.e., RWE, by enhancing glycolysis and decreasing the TCA cycle (Fig. 4). These downstream molecules could be potential cancer therapy targets by interrupting CAFs–epithelia coupling.

In addition to the downstream components of TGF-β signaling shown above, ROS have been described as upstream components of TGF-β signaling that mediate CAFs–epithelia glucose metabolism coupling. ROS are a byproduct of biological reactions and are mainly produced in mitochondria through oxidative metabolism. Moreover, they are one of the main factors responsible for metabolic reprogramming [172]. ROS and TGF-β signaling, two essential regulators of cancer, undoubtedly interact to promote cancer progression. Martinez-Outschoorn et al. [173] reported that MCF-7 breast cancer cells secrete ROS that can trigger oxidative stress in neighboring CAFs, and oxidative stress significantly reduces mitochondrial activity and increases glucose uptake in CAFs. This study indicated that the metabolic coupling between cancer cells and CAFs is mutualistic and that ROS act as messengers from cancer cells to CAFs and strengthen glycolysis in CAFs. ROS also activate TGF-β signaling in other settings. Long-term radiation induces damage to mitochondria via an increase in mitochondrial ROS levels in fibroblasts. Subsequently, mitochondrial ROS activate TGF-β signaling, which in turn mediates the expression of α-SMA in radiation-induced myofibroblasts [174]. In this way, fibroblasts are activated and transformed into a CAF phenotype, leading to tumor growth by enhancing angiogenesis. These data suggest that ROS lead to TGF-β-induced CAF transformation. Since ROS can also increase glycolysis, which is elevated in CAFs, it is reasonable to propose the hypothesis that ROS-induced glycolysis drives the transformation of fibroblasts to CAFs. Conversely, other researchers have reported that TGF-β signaling increases ROS levels in lung CAFs [175]. This study revealed “ROS-TGF-β-ROS” as a positive loop that influences ROS-mediated metabolic coupling between cancer cells and CAFs (Fig. 4).

TGF-β-mediated metabolic coupling can also correlate with glycogen metabolism. Cancer cell-derived TGF-β cytokines trigger TGF-β non-canonical p38-MAPK signaling via paracrine signaling, which stimulates the production of several cytokines from CAFs that induce glycogen metabolism upregulation in cancer cells via phosphorylation and activation of phosphoglucomutase 1, an enzyme involved in glycogenesis. Then, glycogen is used in glycolysis. Furthermore, this study revealed that deletion of p38 in CAFs or glycogen phosphorylase inhibition in cancer cells reduces metastasis [70]. These results suggest that p38-MAPK non-canonical TGF-β signaling in CAFs initiated by TGF-β ligands from cancer cells increases glycogen accumulation in cancer cells, and glycogen as an energy source can be used by cancer cells to facilitate the growth of metastatic tumors. Therefore, TGF-β-activated CAFs not only directly “feed” cancer cells with nutrients but also promote the nutrient synthesis of cancer cells via the cytokine paracrine pathway (Fig. 4). Thus, glycogen synthesis is another TGF-β-dependent CAFs–epithelia metabolic coupling target.

TGF-β signaling is also involved in the lipid and amino acid metabolism of CAFs. CAV-1-depleted fibroblasts exhibit increased levels of intracellular cholesterol and improved TGF-β1 levels via AKT activation, contributing to the metastatic behavior of tumor cells [176]. Since CAV-1 downregulation is a common event in CAFs induced by TGF-β activation, it is reasonable to believe that TGF-β signaling results in a decrease in CAV-1 and cholesterol accumulation in CAFs. CAV-1-induced TGF-β1 production mediates CAF–cancer cell coupling. Further studies are needed to evaluate this hypothesis. Branched-chain amino acids (BCAAs) have been correlated with an increased risk of PDACs. BCAA transaminase 1 (BCAT1) first deaminates BCAAs to branched-chain α-ketoacids (BCKAs). Zhu et al. [177] found that PDAC cancer cells have a marked BCKA reliance on PDAC cell proliferation. The TGF-β/SMAD5 axis directly upregulates the BCAT1 activity of CAFs to allow CAFs to produce more BCKAs, which can be absorbed by cancer cells directly (Fig. 4) [177]. This study revealed TGF-β and BCAT1 as feasible therapeutic targets in PDAC by abrogating BCKA nutrient transfer from CAFs to cancer cells. In conclusion, TGF-β signaling contributes to CAF–epithelial lipid and BCKA metabolism coupling through CAV-1 downregulation and BCAT1 enzyme modulation. CAV-1 adjusts both RWE and lipid metabolism coupling; therefore, it is a common target of CAF–epithelial glucose and lipid metabolism coupling in CAFs.

### Immune cells–epithelia metabolism coupling

**Fig. 5:**
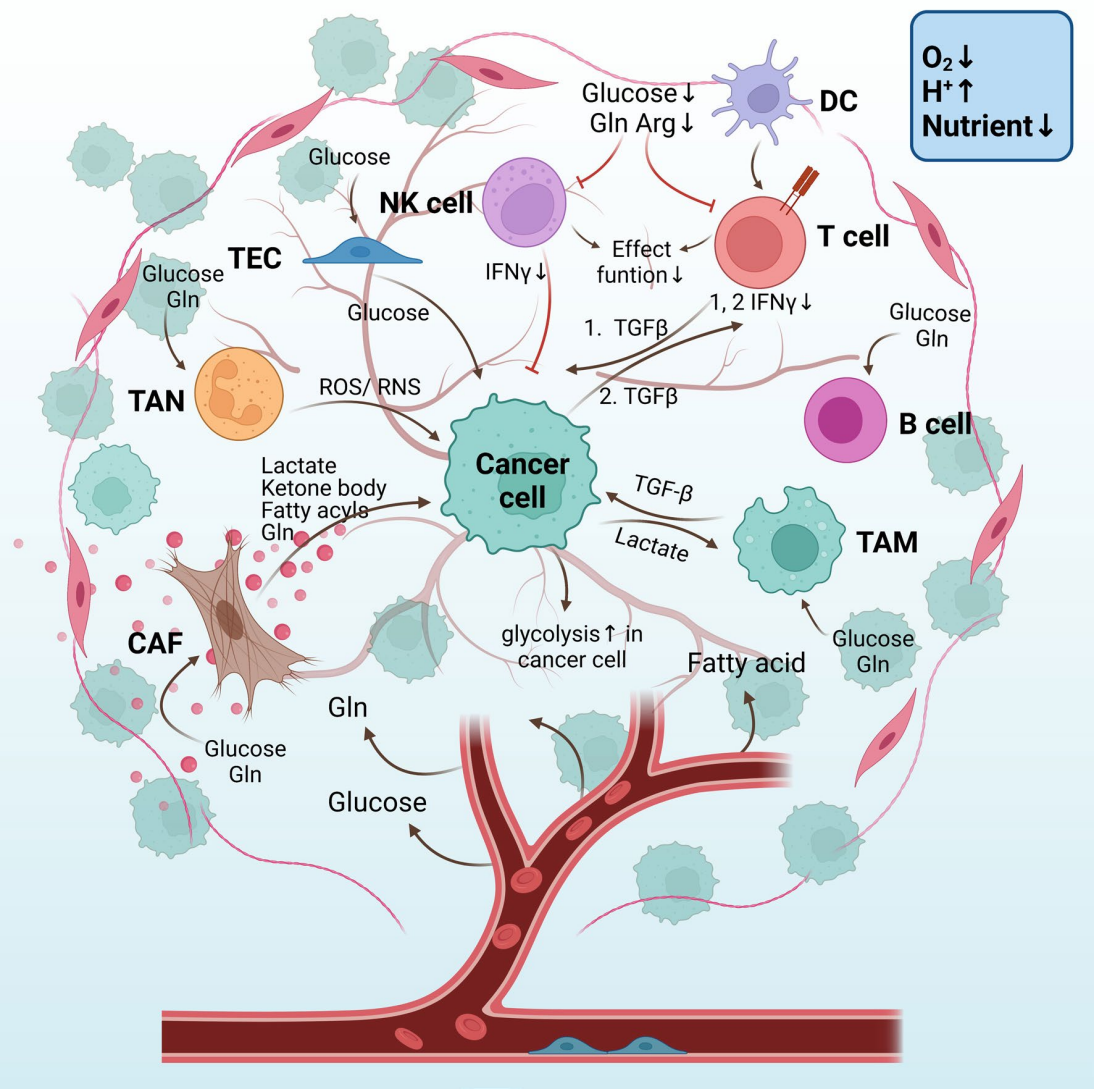
The “fruit tree” schematic diagram of the metabolic coupling between stromal and cancer cells through nutrients and metabolites. CAFs provide mitochondrial fuels for cancer cells, including lactate, ketone body, fatty acyls, and Gln. TANs can release ROS and RNS to kill cancer cells directly. TECs absorb glucose molecules and then provide them to cancer cells. TAMs are influenced by lactate that is exported from cancer cells, while TAMs provide cancer cells with TGF-β which elevated the glycolytic rate of cancer cells. Malignant B cells tend to be glycolytic and Gln-addicted. Since all cells compete for nutrients of glucose, fatty acid, and amino acid, under this nutrient-deficient TMME, T cells and NK cells are immunosuppressive with less IFNγ releasement. TGF-β-mediated metabolic coupling with cancer cells mainly occurs in T cells based on previous literature. 1. Cancer cells with increased aerobic glycolysis by HK2 expression are easier to “educate” antitumor CD4 + T cell to be immune-suppressed through ways below: Stimulate CD4 + T cell secret TGF-β, an immune-suppressing cytokine; and glucose competition between cancer cells and CD4 + T cells lowers glycolytic rate in CD4 + T cells, which decreases the antitumor activity of CD4 + T cells by IFNγ production decline. 2. Cancer cell-derived TGF-β can also decrease INFγ secretion of CD4+ T cells. Few/abnormal blood vessels lead to a hypoxic TMME(O_2_↓) which stimulates aerobic glycolysis in cancer cells and stromal cells. Lactate produced by glycolysis contributes to an acidic TMME(H^+^↑). Since all cells in TMME compete for the restricted nutrient, therefore nutrient is deficient (Nutrient↓) in TMME. CAFs: cancer-associated fibroblasts, Gln: glutamine; Arg: arginine; TAN: tumor-associated neutrophils; ROS: reactive oxygen species; RNS: reactive nitrogen species. TECs: tumor endothelial cells; TAMs: tumor-associated macrophages; Arg: arginine; TMME: tumor metabolic microenvironment; and HK2: Hexokinase 2

Immune cells can either control or advance tumor development during different disease stages. Depending on whether immune responses are specific, immune cells are divided into two categories: innate and adaptive immune cells. Innate immune cells include natural killer (NK) cells, macrophages, neutrophils, and dendritic cells, while adaptive immune cells consist of T cells and B cells [178]. These cells are usually educated by cancer cells to be immunosuppressed, allowing their immune evasion, and immune cells have a dynamic crosstalk with tumor cells and their surrounding environment [41]. Cellular immunometabolism, a branch that studies the role of metabolic reprogramming in immune cell function, influences cancer development by modulating the immunosuppressed or effector function of immune cells [179]. Herein, we will describe how cellular immunometabolism influences the activity of immune cells, immune cells–epithelia coupling, and the role played by TGF-β signaling in this process (Fig. 5, the “fruit tree” schematic diagram).

#### Metabolic phenotypes of innate immune cells

NK cells are cytotoxic lymphocytes of the innate immune system capable of killing cancerous cells [180]. Accumulated evidence has shown that their effector functions are closely linked to cellular metabolism [181]. Upon activation, NK cells exhibit increased glucose uptake, as evidenced by elevated GLUT1 expression, and glycolysis is subsequently enhanced [182, 183], indicating that glucose is a major nutrient supporting NK cell activity. Since NK cell activity is suppressed in the tumor, it is rational to hypothesize that glucose restriction, a hallmark of TMME, may reduce glycolysis and impair cancer cell toxicity. Cong et al. [184] proved this hypothesis in a murine lung cancer model. They observed increased expression of fructose-1,6-bisphosphatase, an enzyme that inhibits glycolysis, in NK cells of the lung cancer microenvironment. NK cell effector functions can be regained by the inhibition of fructose-1,6-bisphosphatase [184]. These data imply that the antitumor effect of NK cells can be strengthened by glycolysis upregulation. Fatty acid metabolism may also influence NK cell activity in the tumor. NK cells with high lipid content have a diminished ability to lyse cancer cells in both preclinical surgical models and human surgical colorectal cancer patients. A study using a mouse model further elucidated that increased lipid accumulation in NK cells after surgery is due to the upregulation of MSR1, CD36, and CD68 [185]. MSR1, CD36, and CD68 could be cancer targets for patients with surgical colorectal cancer by elevating NK cell toxicity. Cancer cells are addicted to Gln, as discussed above, so the TMME is also deficient in Gln. Data have shown that when activated NK cells are cultured in Gln-deficient conditions, OXPHOS and glycolysis significantly decrease and IFNγ production is substantially inhibited [186]. This study demonstrated the importance of amino acid (Gln) metabolism for NK cell activation, and Gln metabolism is linked with glucose metabolism. NK cell function is not affected by Gln metabolism inhibitors, but cancer cells are affected. We predict that these inhibitors could result in Gln accumulation within the TMME owing to decreased utilization by cancer cells, which would facilitate the antitumor functions of tumor-infiltrating NK cells. These findings indicate the efficacy of anticancer therapies using Gln metabolism inhibitors. In summary, enhancing glycolysis, impeding lipid accumulation and Gln metabolism in NK cells could be potential directions for facilitating their tumor-killing ability.

Macrophages are known to be one of the most abundant immune cells in the TME and can influence cancer progression [187, 188]. The metabolic phenotypes of macrophages in terms of glucose, lipids, and amino acids are altered to some degree. For glucose metabolism, tumor extract-stimulated bone marrow-derived macrophages, which mimic tumor-associated macrophages (TAMs), show an upregulated molecular signature of glycolysis, and its key enzyme, HK2, is elevated [189]. A similar phenomenon was observed by Arts et al. [190] where the coculture of monocytes from a healthy donor with thyroid cancer cells resulted in differentiated macrophages displaying a metabolic transcriptomic signature with increased glycolysis and activation of the AKT1/mammalian target of rapamycin (mTOR) pathway, an essential regulator of cell metabolism [190]. Therefore, glycolysis is enhanced in TAMs and should be responsible for its features in the tumor. Lipids exert a crucial role in TAM generation. Su et al. [191] demonstrated that human and murine TAMs harbor enriched lipid accumulation via an increase in CD36, a scavenger receptor that is responsible for lipid uptake. Additionally, elevated FAO in TAMs simultaneously occurs. CD36-KO TAMs lose their tumor proliferation-promoting ability in vitro and vivo and exhibit an M1-macrophage gene signature [191]. These results demonstrate that the inhibition of lipid uptake by CD36 in TAMs suppresses their function as pro-tumor cells. In terms of amino acid metabolism, TAMs, particularly the M2 type, serving as protumorigenic TAMs, show increased Arg and Gln consumption. Increased lactate in the TME favors the catabolism of Arg, resulting in increased secretion of tumor-supporting factors (i.e., ornithine and polyamines) by TAMs. Gln restriction impairs M2 polarization, with concomitant TCA cycle downregulation [192], implying that Gln metabolism is essential for TAM properties. In conclusion, enhanced glycolysis, lipid uptake, lipid accumulation, FAO, Arg and Gln catabolism are the metabolic “engines” that sustain the tumor-supporting features of TAMs. Targeting these processes may benefit cancer treatment.

Neutrophils are the most abundant circulating leukocytes in humans, and they have been recently known as an essential component of the innate immune system involved in cancer development [193–195]. Neutrophils release ROS and reactive nitrogen species (RNS) and then cause cancer cell gene damage and mutation that can both lead to carcinogenesis or cancer cell death. This demonstrates the dual roles and plasticity of neutrophils in cancer. Neutrophil metabolism has a heavy reliance on glycolysis due to the limited number of mitochondria [196]. Ancey et al. [197] applied a mouse model of lung adenocarcinoma and found that, compared with normal neutrophils, GLUT1 and glucose metabolism are increased in tumor-associated neutrophils (TANs). *Glut1* deletion or loss of GLUT1 reduces the number of TANs. Furthermore, in the absence of GLUT1 in TANs, tumor growth decreases, and radiotherapy efficacy is enhanced [197]. These results underline the importance of GLUT1 and glucose metabolism in TANs, and decreasing glucose uptake can change neutrophils into the pro-tumor subtype. Targeting glucose metabolic alterations in TANs is a promising strategy to favor antitumor neutrophils. Further studies are required to investigate the metabolic alterations of TANs and their metabolic targets in the future. Tumor-elicited neutrophils have previously been characterized as a type of myeloid-derived suppressor cells (MDSCs), a heterogeneous population of immature neutrophils and monocytes with functional differences from healthy blood neutrophils [198]. MDSCs are pathologically activated neutrophils and monocytes with immunosuppressive activity [199]. MDSCs promote tumor growth by inhibiting T cell responses and promoting cancer cell proliferation and migration [200]. MDSC metabolism mainly depends on glycolysis, but MDSCs also acquire energy from the TCA cycle, FAO, and other lipid metabolism pathways to sustain their own survival and tumor activity [201, 202]. MDSCs from humans and mice are all characterized by a high uptake of free fatty acids and increased expression of FAO enzymes. Selectively targeting fatty acid metabolism of MDSCs by etomoxir can impede their immune suppression [203], demonstrating that targeting FAO may serve as a useful approach to hinder the immune-suppressive function of MDSCs. Glutaminolysis can be engaged in neutrophils when the glucose supply is insufficient. In experimental mouse models injected intraperitoneally with the murine ovarian cancer cell line ID8, TANs were shown to use Gln as a major fuel for OXPHOS to support their immunosuppressive roles. These data revealed that Gln catabolism is upregulated in TANs. Taken together, TANs have enhanced glycolysis, FAO, and glutaminolysis, and these catabolism pathways sustain the immunosuppressive features of TANs.

Dendritic cells are major antigen-presenting cells in the human body and are responsible for T cell activation [204]. They can process and present antigens and express them on major histocompatibility complex, and then antigen-specific T cells recognize and induce a specific immune response. Once activated, they mainly rely on glycolysis and PPP to maintain their energy demand and sustain their migration [205]. DCs show lipid accumulation, which results in upregulated fatty acid synthesis in cancer cells and DC lipid uptake from the TME. Lipid accumulation reduces the antigen-processing capacity of DCs and causes them to produce the more tolerogenic cytokine IL-10 [206]. Amino acid metabolism, including Trp and Arg, is essential for dendritic cell function [207]. However, it is necessary to study the metabolic reprogramming of DCs within the TME and how these reprogramming properties influence DC function.

#### Metabolic phenotypes of adaptive immune cells

Cancer cells can evade immune surveillance due to T cell dysfunction. Tumor-infiltrating T cells often partially lose their effector function, and the underlying mechanisms are to some extent related to cell metabolism. CD4^+^ and CD8^+^ T cells in a quiescent state generate most of their energy using the TCA cycle, as their biosynthesis needs are limited and they oxidize glucose, lipids and amino acids for energy production [208]. However, once T cells are activated, a conversion to glycolysis and stimulation of anabolic pathways occur, and the metabolic intermediates of glucose metabolism are used to synthesize biomass-like proteins, lipids, and nucleic acids [208]. Therefore, the shift to anabolism enables T cells to accumulate more energetic nutrients for their activity.

CD4^+^ T cells can be stimulated and differentiated into effector T cell (Teff) or inducible regulatory T cell (Treg) subsets. Michalek et al. showed that Teffs and Tregs require distinct metabolic programs to support these functions [209]. Th1, Th2, and Th17 Teff cells express high levels of GLUT1 and therefore are highly glycolytic. In contrast, Tregs express low GLUT1 levels and exhibit high lipid oxidation rates regulated by activated AMP-activated protein kinase [209]. This study demonstrated the importance of glycolysis for CD4^+^ Teff toxicity and lipid oxidation in Treg cells. However, glycolysis inhibition with 2-deoxy-D-glucose favors memory CD8^+^ T cell antitumor function [210], implying that glycolysis inhibits the effector function of CD8^+^ T cells. These studies revealed that the roles of glycolysis are not consistent in different types of T cells. In addition to glucose metabolism, cholesterol, as a kind of lipid, in the TME induces the expression of immune checkpoints in CD8 + T cells, and cholesterol deprivation can rescue CD8^+^ T cell effector function [211]. The depletion of amino acids, such as Arg and Trp, impairs effector T cell recruitment and tumor cell toxicity [212, 213], demonstrating that Arg and Trp are linked to T cell effector functions within the TMME. Overall, T cells rely on glycolysis, cholesterol, Arg, and Trp metabolism alterations to regulate their activity and effector function; however, metabolic reprogramming may be specific for each type of T cell.

Studies concerning metabolic remodeling of B cells have focused on B cell malignancy. In Burkitt lymphoma cells, elevated Myc and HIF-1α induce the expression of HK2 and PDK1, enzymes that inactivate pyruvate dehydrogenase and decrease mitochondrial respiration, thereby favoring aerobic glycolysis in malignant B cells [214]. Myc also promotes constitutive expression of lactate dehydrogenase A [215], which diverts glucose-derived pyruvate into lactate, thereby preventing its conversion to acetyl-CoA and its further oxidation in the TCA cycle. Myc-transformed cells also display increased mitochondrial mass and O_2_ consumption [215, 216], indicating that OXPHOS may also be upregulated. Myc activity is also associated with increased Gln metabolism [217]. In summary, malignant B cells represent metabolic reprogramming traits similar to those of solid cancer cells, i.e., elevated glycolysis and heavy Gln metabolism reliance, but the role of infiltrating B cells in solid tumors has not been systematically examined.

#### TGF-β signaling in immune cells–epithelia coupling

TGF-β signaling is instrumental for the immunosuppressive properties of innate and adaptive cells, thereby attenuating the antitumor ability of the major immune cells within the TME [5, 218]. Considering that metabolism is closely correlated with their effector function, it is reasonable to propose that there is a tight relationship between TGF-β signaling and cellular immunometabolism (Table 3). Many studies on TGF-β signaling in cellular immunometabolism have shown that activation of this cascade represses cancer immune function, especially in NK and T cells. The regulatory effect of TGF-β signaling on NK cell metabolism has been extensively studied. Slattery et al. [219] showed that NK cells in metastatic breast cancer patients are exhausted and have metabolic defects including reduced glycolysis and oxidative phosphorylation. TGF-β signaling is responsible for these effects in patients. Blocking TGF-β signaling with anti-TGF-β antibodies restores IFNγ production in patient NK cells in vitro; therefore, NK cells have increased oxidative glucose metabolism and glycolysis partially by mTORC1 activity rescue [219]. These results suggest that non-canonical TGF-β signaling participates in NK cell immunometabolism. Similarly, Zaiatz-Bittencourt et al. [220] observed that human NK cell activation induces increased oxidative phosphorylation and glycolysis. TGF-β can inhibit these metabolic changes, and inhibition of the TGF-β signaling pathway by B431542, a TGF-βRI inhibitor, is able to restore metabolic and functional response alterations induced by TGF-β [220]. These results demonstrate that TGF-β signaling is responsible for the downregulation of glucose metabolism and thus leads to the repression of NK cell function. Inhibiting TGF-β signaling is a feasible plan to enhance the effector function of NK cells in cancer via metabolic advantages.

**Table 3:** TGF-β-dependent stromal cell metabolic reprogramming in cancer

CAFs	NK cells (exhausted)	macrophages	Neutrophils/MDSCs	T cells (exhausted)	B cells
					
*Stromal cell metabolic reprogramming*					
Glycolysis↑	Glycolysis↓	Glycolysis↑	Glycolysis↑	Glycolysis↓	Glycolysis↑
Fatty acid synthesis↑	Lipid accumulation ↑	Lipid accumulation ↑ FAO↑	FAO↑	Cholesterol↑ FAO↑	Further studies needed
Gln anabolism↑	Gln catabolism ↓	Gln and Arg catabolism↑	Gln catabolism↑	Arg and tryptophan metabolism↓	Gln catabolism↑
*TGF-β-dependent stromal cell metabolic reprogramming*					
CAV-1↓ or ROS↑-Glycolysis↑	mTOR↓-Glycolysis↓	OXPHOS↑-M2 macrophages↑	Arginase↑-Pro-tumor features ↑	OXPHOS↑ and glycolysis↓, FAO ↑-Tregs↑	Further studies needed
a.IDH3α↓PDK1↑-TCA cycle↓ b. BCAT1 ↑-BCKAs↑	mTOR↓-OXPHOS↓	Arginase↑-Pro-tumor features ↑	CD39 and CD73↑-adenosine↑-Pro-tumor features ↓	ATP synthase↓-IFNγ↓-Effector function↓	Further studies needed

*MDSC* myeloid-derived suppressor cells; *Gln* glutamine; *FAO* fatty acid oxidation; *Arg* arginine; *CAV-1* caveolin-1; *ROS* reactive oxygen species; *IDH3α* isocitric dehydrogenase 3; *BCAT1* branched chain amino acid transaminase 1; *BCKAs* branched-chain α-ketoacids; *mTOR* mammalian target of rapamycin; *OXPHOS* oxidative phosphorylation; *Tregs *regulatory T cells

The TGF-β-mediated metabolic shift leads to the phenotypic plasticity of immune cells, such as TAMs. Park et al. [221] recently showed that exosomes derived from cancer cell lines cultured in hypoxic conditions are highly enriched in TGF-β. These exosomes are able to promote infiltrating myeloid cell polarization toward M2-TAMs and to boost their effector functions by enhancing OXPHOS. In macrophages, TGF-β has been reported to upregulate arginase activity [222], which catalyzes Arg, and its activity is positively related to the immunosuppressive function of M2-TAMs [223]. Therefore, TGF-β signaling may upregulate OXPHOS and Arg catabolism to switch macrophages to M2 subtypes in cancer. M2-TAMs can also influence cancer cells via TGF-β. Anti-inflammatory M2-TAMs secrete the cytokine TGF-β, which decreases the TCA cycle metabolic enzyme succinate dehydrogenase (SDH) and results in the accumulation of succinate in human breast cancer cells. Then, the accumulated succinate enhances the stability of HIF-1α and reprograms cell metabolism to a highly glycolytic state [224]. This finding revealed that TGF-β produced by TAMs can alter metabolism in cancer cells via paracrine.

Under TGF-β exposure, neutrophils undergo N2 polarization [225]. Similar to M2 macrophages, N2 neutrophils are also immunosuppressive and benefit cancer progression. The metabolism of N2-TANs is characterized by a high level of arginase 1 and iNOS expression, which are involved in Arg catabolism and contribute to T cell exhaustion in tumors. This study suggested that Arg catabolism, like in macrophages, mediates the immunosuppressive feature of N2-TANs, and TGF-β may also exert a role to some extent. Breast, lung, melanoma or colon cancer cell lines were injected intravenously into mouse models of cancer metastasis, and TGF-β-stimulated TANs expressed higher levels of arginase 1 and iNOS [226]. This study verified the role of TGF-β signaling in forming N2-TANs by increasing Arg catabolism. Two enzymes, CD39 and CD73, are upregulated in MDSCs, a type of immature neutrophil, and they can catabolize ATP to generate extracellular adenosine, a well-known inhibitor of antitumor immunity [227, 228]. Li et al. [229] reported that the lasting activation of these two ATP metabolizing enzymes in MDSCs from NSCLC patients is triggered by TGF-β-mTOR-HIF-1 signaling. Therefore, TGF-β signaling links the immunosuppressive features of N2 neutrophils and MDSCs with Arg and ATP catabolism, respectively. These catabolism pathways produce immunosuppressive substances, such as arginase 1, iNOS and adenosine, providing novel targets for immunometabolism intervention of MDSCs.

The regulatory effect of TGF-β signaling on adaptive immune cell metabolism mainly focuses on T cell metabolism, especially CD4^+^ T cells. TGF-β treatment of CD4^+^ T cells induces the conversion of CD4^+^ T cells into Treg cells, which have high oxidative metabolism and limited glycolysis, by lowering the expression of glycolytic genes, such as *Glut1* and *Hk2,* and promoting the inhibition of the TCR-CD28-PI3K-mTOR pathway [209]. Concurrently, the mitochondrial membrane potential and respiratory capacity are increased, which is further associated with increased FAO, thus providing intermediates for the TCA cycle [230]. These results demonstrate that TGF-β signaling is responsible for the metabolic features of Tregs, i.e., elevated FAO and downregulated glycolysis. Therefore, TGF-β-mediated immunometabolism weakens antitumor immune function and contributes to the formation of an immunosuppressive tumor microenvironment. Cancer cells and CD4^+^ T cells show metabolic coupling, and the coupling is mediated by TGF-β signaling. Ho et al. [231] observed that mouse melanoma cancer cells with increased aerobic glycolysis induced by HK2 expression more easily “educate” antitumor CD4^+^ T cells to be immunosuppressed, and CD4^+^ T cell secretion augments TGF-β, an immune-suppressing cytokine. These results suggest that glucose competition between cancer cells and CD4^+^ T cells induces glucose deprivation, which impairs antitumor ability in CD4^+^ T cells partially because of TGF-β signaling activation. Furthermore, in this setting, CD4^+^ T cells display decreased glycolytic metabolite PEP and increased PEP production through phosphoenolpyruvate carboxykinase 1 (PCK1) overexpression-boosted effector functions. [231] Moreover, PCK1-overexpressing T cells restrict tumor growth and prolong the survival of melanoma-bearing mice. A similar phenomenon was also observed in CD8 + T cells [231, 232]. From these results, we can conclude that the Warburg effect of cancer cells represses the antitumor ability of T cells through the secretion of TGF-β and downregulation of aerobic glycolysis in T cells via glucose competition. Consistently, highly glycolytic melanoma cells also compromises the efficacy of T cell immunotherapy, including adoptive T cell therapy and anti-PD-1 treatment, through an impaired T cell killing ability [233, 234]. In these settings, restricting glycolysis in cancer cells improves therapeutic efficacy [233, 234], indicating that targeting glycolysis in cancer cells, such as HK2 and PEP, is a potential candidate for combinatorial therapeutic intervention by interrupting immune cells–epithelia coupling and thus increasing T cell antitumor activity. Another study demonstrated that TGF-β derived from tumors specifically inhibits mitochondrial complex V (ATP synthase) activity and thus impairs the inhibition of mitochondrial complex V (ATP synthase) activity in CD4^+^ T cells [235]. In this way, ATP synthase inhibition alone causes IFNγ production impairment in CD4^+^ T cells [235]. These data demonstrate that TGF-β secreted by cancer cells directly diminishes the effector function of immune cells, i.e., T cells, through metabolic paralysis. In summary, TGF-β mediates immune cells–epithelia coupling via the downregulation of T cell glycolysis by competition or ATP synthesis paralysis, thereby decreasing T cell effector function and ultimately promoting cancer progression. Further studies are necessary to identify additional TGF-β-dependent immune cells–epithelia coupling targets that enable sensitive T cell-based cancer immunotherapy.

### Endothelia–epithelia metabolism coupling

Blood vessels are crucial for oxygen and nutrient transportation to the tumor. Cancers depend on blood vessels for oxygen and nutrient supply. ECs are the single cell layer that lines blood vessels and regulates exchanges between the bloodstream and the surrounding tissue. TGF-β can modulate angiogenesis and induce endothelial–mesenchymal transition (EndMT), a phenomenon in which ECs undergo morphological, functional, and molecular changes, including a decrease in their adhesion protein and increased expression of mesenchymal biomarkers [236]. Similar to EMT, EndMT may also undergo metabolic reprogramming. We will discuss EC metabolic reprogramming and its potential relationship with TGF-β signaling below.

Tumor vessels are highly abnormal in their structure and function. They are hyperproliferated, thus rapidly forming blood vessels to sustain tumor growth. ECs display the Warburg effect like cancer cells, i.e., they are highly glycolytic. Transcriptomic profiling combined with metabolomics, tracer, and flux analysis of mouse B16-F10 tumor ECs (TECs) revealed that these cells rely more on glycolysis than normal ECs (NECs), and glycolytic activator PFKFB3 blockade induces cancer cell invasion, intravasation, and metastasis by normalizing tumor vessels [237]. Other researchers have observed a similar phenomenon in mouse ovarian TECs, and they further reported that TECs increase glycolysis via upregulation of vascular endothelial growth factor (VEGF) by cyclooxygenase 2 [238]. Importantly, TECs push glycolytic intermediates to the PPP and serine biosynthesis pathway to generate building blocks for nucleotide synthesis [237]. To conclude, TECs are prone to glycolysis and PPP, thus sustaining their proliferation by producing biomacromolecule precursors. Similar to other cells in tumors, TECs also express increased FASN levels to increase lipid synthesis. Under conditions of this glycolytic restriction, Gln contributes to endothelial ATP synthesis and improves cell viability, [239] suggesting that Gln is a substitute for TECs when glucose is deficient.

TGF-β signaling affects angiogenesis through the activin receptor-like kinase 1 (ALK-1) interaction, which is specifically expressed in vascular ECs. TGF-β can bind to ALK-1, activating EC proliferation via p-SMAD1/5 signaling [240]. ECs can also be triggered by TGF-β to undergo EndMT [241]. Since proliferation and EndMT all require energy, similar to cancer cells, we hypothesized that TGF-β signaling also regulates EC metabolic reprogramming. As the enzyme in the first step of PPP, G6PD deficiency activates endothelial cell and leukocyte adhesion via the TGF-β/NADPH oxidases/ROS signaling pathway and thus increases the risk of cardiovascular disease. Xiong et al. demonstrated that endothelial FAO is essential to maintain endothelial cell features and that FAO disruption thickens the cardiac valve by inducing TGF-β-dependent EndMT [242]. However, these studies are all about TGF-β-dependent EC metabolic reprogramming in heart diseases. Therefore, the relationship concerning TGF-β-TEC metabolic reprogramming-cancer remains to be explored in the future.

TGF-β is a key mediator of angiogenesis, which results from crosstalk between the endothelium and other cells induced by TGF-β [243]. During vessel maturation, TGF-β secreted by the endothelium induces mesenchymal cells to differentiate into pericytes and smooth muscle cells, which contributes to angiogenesis [244]. These findings suggest that endothelial cells produce TGF-β and alter neighboring mesenchymal cells into blood vessel cellular components. Zonneville et al. [245] demonstrated that tumor-derived TGF-β enhances tumor vascularization by increasing pericyte–endothelium contraction via a TGF-β-fibronectin axis. Inactivation of tumor TGF-β signaling reduces the blood vessel density and lumen size, decreasing tumor growth, suggesting the potential therapeutic effect of targeting TGF-β signaling against angiogenesis, thereby impeding tumor growth.

Recently, metabolic crosstalk between the endothelium and other cells, including cancer and immune cells, has been reported to influence tumor progression. In glioma, tumor cells suffering from hypoxia can secrete VEGF to upregulate GLUT1 expression in the brain endothelium. This process consequently allows more glucose molecules to cross glucose transporters of endothelial cells and then be delivered into the tumor [246]. These data indicate that the interruption of glucose uptake via glucose transporter inhibitors may alter endothelial–cancer cell metabolic coupling, leading to reduced tumor growth. Hypoxic TAMs strongly upregulate the expression of REDD1 to hinder glycolysis in TAMs and curtail their excessive angiogenic response, with consequent formation of abnormal blood vessels through aberrant vascular junctions [247]. This study revealed that the glucose metabolism of TAMs may influence endothelial cell junctions and thus hinder tumor angiogenesis. Taken together, cancer cells may alter the glucose metabolism of endothelial cells, and the glucose metabolism of TAMs can influence endothelial cell junction formation. These endothelial metabolic couplings with cancer or immune cells further control tumor growth. TGF-β1 has been demonstrated to engage in Gln metabolism in endothelial cells [248]; however, the roles of TGF-β signaling in the processes of metabolic crosstalk remain unclear.

### Other cells in tumor metabolic microenvironment

Adipocytes are the cells that primarily compose adipose tissue for storing energy as fat*.* Autophagy in human adipocytes can promote free fatty acid release and is then utilized for ovarian cancer cell proliferation [249]. Clement et al. [250] showed that human adipocyte vesicles transfer lipids and enzymes to stimulate FAO in melanoma cells. This FAO-stimulated mitochondrial activity redistributes mitochondria to membrane protrusions of migrating cells, which is instrumental for melanoma cell migration. Moreover, metastasis-on-a-chip indicated that adipocyte-derived lipids induce human cancer cell migration via cancer cell HIF-1α activation [251]. In conclusion, adipocytes mainly promote cancer progression by lipid transfer to cancer cells, providing an energy source for cancer cell proliferation and migration.

Erythrocytes can exist in the tumor as clotting and liquid blood, and only a few studies have been found concerning their role in cancer progression until now. A recent study by Karsten et al. [252] showed that erythrocytes cocultured with the NSCLC A549 cell line have elevated production of the cytokines IL-8, basic fibroblast growth factor, and VEGF. In addition, this coculture system promotes CD8 + T cell expansion and immune function [252]. Hercbergs A et al. [253] demonstrated that a high peripheral erythrocyte glutathione concentration may contribute to resistance to cancer chemotherapy. These studies demonstrate that erythrocytes are also crucial for cancer immunology and chemotherapy response. More attention may need to be paid to tumor-infiltrated erythrocytes in cancer development and the potential role of TGF-β signaling in erythrocyte metabolism.

## TGF-β-mediated host metabolism reprogramming in cancer

**Fig. 6:**
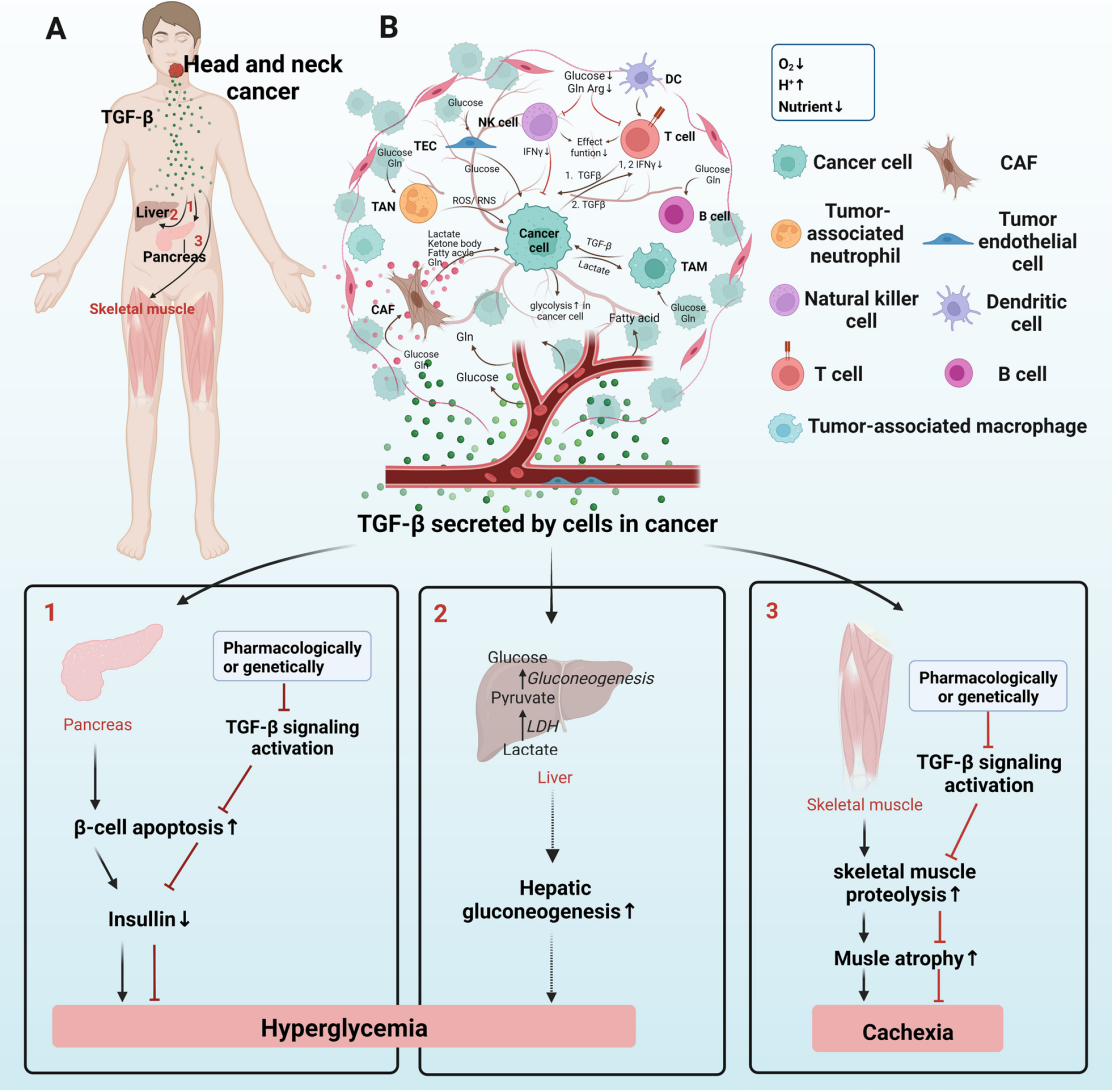
TGF-β-mediated host metabolism reprogramming in cancer. **A** Cancer is like a malignant organ that produces a large amount of TGF-β cytokine, then it circulates all over the host body including the liver, pancreas, and skeletal muscle. **B** In this way, TGF-β ligand resembles an endocrine factor and leads to hyperglycemia through 1. inducing β-cell apoptosis (verified) and 2. hepatic gluconeogenesis (hypothesized). 3. Cachexia is induced by TGF-β signaling by skeletal muscle proteolysis (verified). Pharmacologically or genetically inhibiting TGF-β signaling can attenuate these processes and reverse hyperglycemia and cachexia.

In the sections discussed above, we focused on metabolism in the local tumor. However, cancer and inflammatory cells result in the accumulation and release of soluble factors from the TME into the circulatory system. These factors result in pathological endocrine effects, thus allowing for interaction between the TME and the patient’s organs and systems, leading to the development of cancer-associated systemic metabolic alterations, i.e., host metabolism reprogramming in cancer (Fig. 6).

Hyperglycemia, or high blood glucose, is a condition in which there is an excess amount of glucose in the blood circulation. A recent study reported that patients with pancreatic cancer may develop hyperglycemia or diabetes 2–3 years before the diagnosis of pancreatic cancer [254]. TGF-β signaling has been documented to cause β-cell apoptosis, which abrogates insulin secretion. Pharmacologically or genetically suppressing TGF-β signaling protects against PDAC-driven β-cell apoptosis [255]. This study revealed a link between TGF-β signaling and cancer hyperglycemia. TGF-β signaling also promotes gluconeogenesis. High serum levels of TGF-β1 have been detected in pancreatic cancer [256], and we consistently detected a higher TGF-β ligand level in metastatic oral squamous cell carcinoma [40]. Both studies demonstrated that the TME produces a large amount of TGF-β1 and may exert an endocrine effect on host metabolism. Intriguingly, TGF-β1/Smad3 signaling has been demonstrated to promote hepatic gluconeogenesis via the regulation of protein phosphatase 2A, AMPK, and FoxO1. Genetic and pharmacological inhibition of TGF-β1/SMAD3 signaling suppresses endogenous glucose production [257]. Therefore, we hypothesized that circulating TGF-β1 derived from the TME may serve as a driver of hyperglycemia through elevated hepatic gluconeogenesis. Taken together, targeting the TGF-β pathway may be a promising alternative treatment against pancreatic cancer-induced hyperglycemia by preventing β-cell apoptosis and reducing hepatic gluconeogenesis.

Cachexia, as an adverse effect of cancer, is a condition that causes severe weight loss and skeletal muscle loss or atrophy [258]. Cancer cachexia is defined as a host metabolic disorder that cannot be fully reversed by conventional nutritional support [259]. Studies have suggested that enhanced autophagy induces impaired mitochondrial function and exacerbates muscle atrophy in tumor-bearing mice [260, 261]. Yang et al. [262] revealed that TGF-β1 promotes atrophy of skeletal muscle or skeletal muscle proteolysis, both in vivo and in vitro*, by* increasing HMGB1/autophagy pathway activity. This study suggested that TGF-β signaling may contribute to muscle atrophy in cancer. As expected, Greco et al. [263] verified that TGF-β blockade using a neutralizing antibody significantly improves overall mortality, weight loss, fat mass, lean body mass, bone mineral density, and skeletal muscle proteolysis in mice with advanced pancreatic cancer. Overall, these studies suggest that TGF-β-targeted therapies may benefit cancer cachexia treatment. Targeting TGF-β signaling is a promising therapy to relieve hyperglycemia and cachexia in cancer patients through host metabolism reprogramming interventions.

## Conclusions

Even though the role of TGF-β signaling in cancer proliferation, migration, invasion, and immune evasion has been extensively studied, the TGF-β signaling-metabolism regulation network is not well evaluated in cancer. Therefore, our review illustrates the effect of TGF-β signaling on cancer metabolism. We define the TMME as the metabolic TME. Cellular metabolism and subsequent metabolic coupling are the foundations of the TMME. TGF-β signaling is a metabolic reprogramming driver for the formation of the TMME by regulating metabolic intermediates. First, the influence of TGF-β signaling on the TMME can drive cellular metabolism toward cancer growth, metastasis, and immune evasion. On the one hand, TGF-β signaling can elevate glycolysis in cancer cells and CAFs, producing a large amount of lactate and forming an acidic TMME. On the other hand, the glycolytic rates of NK cells and T cells are limited to decrease their cancer cell toxicity. Second, TGF-β signaling mainly facilitates the metabolic coupling between cancer and stromal cells, including CAFs, macrophages, and T cells. In this way, CAFs provide cancer cells with energetic metabolites via RWE, macrophage-secreted TGF-β enhances cancer cell glycolysis, and cancer cell-derived TGF-β decreases T cell effector function by ATP production paralysis in T cells. Finally, TGF-β signaling contributes to host metabolism impairment and induces hyperglycemia or cachexia. Targeting TGF-β signaling is promising to reverse abnormal cellular, tissue and organismal metabolism in cancer and is advantageous to cancer treatment. Currently, many TGF-β inhibition agents have entered clinical trials (Table 4), and achieved good clinical efficacy [264]. However, hurdles exist and need to be overcome. Enlightened by this review, we will next elucidate how to face these hurdles from the perspective of TGF-β-dependent metabolic reprogramming.

**Table 4:** TGF-β-targeted therapies in cancer

Agent	Target	Treatment	Application	Experiment status	Clinical outcome	Clinical trial
*Small-molecule inhibitor*						
Galunisertib	TGFβRI	Combination with durvalumab	Pancreatic cancer	I; Completed	Had acceptable tolerability and safety	NCT02734160
		Combination with nivolumab	NSCLC and HCC	I/II; Completed	Some patients exhibited complete or partial remission	NCT02423343
LY3200882	TGFβRI	Combination with pembrolizumab	Advanced cancer	Ib/II; Withdrawn	No results posted	NCT04158700
		Combination with capecitabine	Colorectal cancer	I/II; Not yet recruiting	No results posted	NCT04031872
Vactosertib	TGFβRI	Monotherapy	Solid tumor	I; Completed	No results posted	NCT02160106
		Combination with pembrolizumab	NSCLC	II; Recruiting	No results posted	NCT04515979
PF06952229	TGFβRI	Monotherapy/Combination with enzalutamide	Solid tumor	I; Terminated	No results posted	NCT03685591
TEW-7197	TGFβRI ALK5	Monotherapy	Solid tumor	I; Completed	No results posted	NCT02160106
		Combination with FOLFOX	Pancreatic cancer	I/II; Recruiting	No results posted	NCT03666832
*Neutralizing antibody*						
Fresolimumab	TGFβ1/2/3	Monotherapy	MPM	II; Completed	3 patients (out of 13) showed stable disease	NCT01112293
		Combination with radiotherapy	BC	II; Completed	Suppressed tumor	NCT01401062
NIS793	TGFβ1/2/3	Combination with chemotherapy	PDAC	III; Recruiting	No results posted	NCT04935359
		Combination with PDR001	Advanced malignancies	I; Completed	No results posted	NCT02947165
SAR439459	TGFβ1/2/3	Monotherapy/Combination with cemiplimab	Solid tumor	I; Terminated	No results posted	NCT03192345
		Monotherapy/Combination with novel agents in RRMM	Plasma cell myeloma	I/II; Recruiting	No results posted	NCT04643002
SRK181	TGFβ1	Monotherapy/Combination with anti-PD-(L)1 antibody	Solid tumor	I; Recruiting	No results posted	NCT04291079
ABBV151	GARP-TGFβ1	Monotherapy/Combination with ABBV-181	Solid tumor	I; Recruiting	No results posted	NCT03821935
LY3022859	TGFβRII	Monotherapy	Solid tumor	I; Completed	The maximum tolerated dose was not determined	NCT01646203
Luspatercept	TGFβ1/2/3	Monotherapy	MDS	II; Completed	Was well tolerated and effective	NCT02268383
		Monotherapy	MDS	III; Completed	Reduced the severity of anemia	NCT02631070
AVID200	TGFβ1/3	Monotherapy	Solid tumor	I; Active, not recruiting	No results posted	NCT03834662
M7824	TGFβ1/2/3 and PD-L1	Monotherapy	NSCLC	III	No significant improvement of OS	NCT03631706
		Monotherapy	metastatic BTC	II/III	No results posted	NCT04066491
		Combination with chemotherapy	NSCLC	I/II; Completed	No results posted	NCT03840915
		Monotherapy	BC	I; Completed	No results posted	NCT03524170
*Antisense oligonucleotide*						
AP12009	TGFβ2 mRNA	Monotherapy	Pancreatic cancer, melanoma and CRC	I; Completed	No results posted	NCT00844064
		Monotherapy	GBM	II; Completed	No results posted	NCT00431561
*Vaccine*						
Lucanix	TGFβ2	Monotherapy	NSCLC	II; Completed	No results posted	NCT01058785
		Monotherapy	NSCLC	III; Completed	No results posted	NCT00676507
Vigil ™	TGFβ1/2	Combination with pembrolizumab	Advanced melanoma	I; Completed	No results posted	NCT02574533
		Monotherapy/Combination with temozolomide and irinotecan	Ewing's Sarcoma	II; Completed	Reduce disease burden	NCT02511132
*Integrin inhibitor*						
Cilengitide	Integrins αvβ3 and αvβ5	Monotherapy	Prostate cancer	II; Completed	Had good tolerance but no detectable clinical activity	NCT00121238
		Monotherapy	HNSCC	I/II; Completed	No significant effect	NCT00705016
SF1126	Integrin-targeted PI3 kinase	Monotherapy	Solid tumor	I; Completed	No results posted	NCT00907205
		Monotherapy	HNSCC	II; Terminated	No data available	NCT02644122
IMGN388	Integrin αv	Monotherapy	Solid tumor	I; Completed	No results posted	NCT00721669
Abergrin	Integrin αvβ3	Monotherapy	MM	I; Completed	No results posted	NCT00111696
Volociximab	Integrin α5β1	Combination with gemcitabine	Pancreatic cancer	II; Completed	No results posted	NCT00401570

(Information was obtained from https://www.clinicaltrials.gov/)

*NSCLC* non-small cell lung cancer; *HCC* hepatocellular carcinoma; *CRC* colorectal cancer; *BC* breast cancer; *nal-IRI* liposomal irinotecan; *BTC* biliary tract cancer; *PDAC* pancreatic ductal adenocarcinoma; *GC* gastric cancer; *MPM* malignant pleural mesothelioma; *RCC* renal cell carcinoma; *MM* malignant melanoma; *MDS* myelodysplastic syndromes; *GBM* glioblastoma; *OC* ovarian cancer; *HNSCC* head and neck squamous cell carcinoma; *PPC* primary peritoneal cancer

First, targeting TGF-β signaling at the level of TGF-β isoforms or its receptors has safety concerns due to the control of diverse processes and numerous responses, negative feedbacks may occur and compromise the anti-TGF-β effect. Therefore, targeting downstream metabolic proteins of TGF-β increases the specificity of therapeutic measures. For example, some patients treated with the monoclonal antibody fresolimumab have keratoacanthomas, and this adverse effect can be explained by the loss of the inhibitory effects of TGF-β on keratinocyte proliferation [265]. In this way, selecting approaches to target TGF-β signaling at the level of intracellular mediators, such as enzymes, is safer. Additionally, TGF-β regulates enzymes in glucose, lipid, and amino acid metabolism of cancer cells and can provide us with a regulatory network. Thus, the central metabolic protein that commonly regulates glucose, lipid, and amino acid metabolism in a cell type can be identified. For example, CAV-1 promotes both glucose and lipid metabolism in CAFs. In CAFs, CAV-1 could be a promising target that can fail the “engine” of CAFs by glucose and lipid metabolism correction. Currently, many agents have been designed to target cancer metabolism, thus impeding cancer progression (Table 5). The potential TGF-β-dependent metabolism targets are listed in Tables 1 and 2. Future studies can utilize the agents shown in Table 5 to further explore the feasibility of targeting TGF-β-dependent metabolic proteins for cancer treatment.

**Table 5:** Metabolism-targeted drugs

Strategy	Agent	Experiment status	Application	Cancer biological behavior	Clinical trial/Ref.
GLUTs inhibitor	Silybin	Phase II; In vivo mouse model	Prostate cancer	Suppressed cancer growth	NCT00487721 [271]
	Rapaglutin A	In vivo mouse model; in vitro human cell culture	Breast cancer	Suppressed cancer growth	[272]
HK II inhibitor	Metformin	Phase II	Breast cancer	Inhibited cancer cell proliferation	NCT01266486 [273]
	2-deoxyglucose	Phase I/II	Prostate cancer	Inhibited cancer growth	NCT00633087 [274]
	3-Bromopyruvate	In vitro human cell culture	Liver cancer	Inhibited cancer growth	[275]
CAV-1 inhibitor	Methyl-β-cyclodextrin	In vitro human cell culture	Colorectal cancer	Increased the BITC-induced anti-cancer effect	276
MCT-1 inhibitor	AZD3965	Phase I	Lymphoma	Inhibited cancer growth	NCT01791595 [277]
PDK inhibitor	Dichloroacetate	Phase I; In vitro human cell culture	Advanced solid tumor	Reduced tumor growth and enhanced Adriamycin cytotoxicity	NCT00566410 [278, 279]
	Hordenine	In vitro human cell culture	Lung cancer	Decreased cancer cell proliferation	[280]
IDH inhibitor	Ivosidenib (AG-120)	Phase I	Brain cancer	Inhibited tumorigenesis	NCT02073994 [281]
	Ivosidenib (AG-120)	Phase III	Cholangiocarcinoma	Reduced tumor growth	NCT02989857 [282]
	Enasidenib	Phase I/II	Leukemia	Inhibited cancer growth	NCT01915498 [283, 284]
	Olutasidenib (FT-2102)	Phase I/II	AML	Suppressed tumor growth	NCT02719574 [285, 286]
	Vorasidenib (AG-881)	Phase I	Glioma	Acquired complete remission	NCT02481154 [287]
HIF-1α inhibition	Apigenin	Phase I	Ovarian, prostate and breast cancer	Downregulated tumor angiogenesis	NCT03526081 NCT03139227 [288–292]
	Semaxanib (SU5416)	Phase II	Metastatic melanoma	Reduced tumor metastasis	NCT00017316 [293]
	2-Methoxyestradiol	Phase I; Phase II	Prostate, breast, brain, head and neck cancer and liver cancer	Inhibited tumor growth and angiogenesis	NCT00030095; NCT00592579 [294–296]
	PX-478	Phase I	Solid tumors and Lymphoma	Enhanced radiosensitivity and suppressed tumor growth	NCT00522652 [297–299]
	BAY 87-2243	Phase I	Neoplasms	Impaired OXPHOS and reduced cancers growth	NCT01297530 [300–302]
OXPHOS inhibition	Lonidamine	Phase II; In vivo mouse model	Glioma	Limited tumor growth	[303, 304]
G6PD inhibitor	Dehydroepiandrosterone	Phase I	Breast cancer	Increased the number of monocytes and NK cells	NCT00972023 [305]
	Polydatin	In vitro human cell culture	Breast cancer	Increased cancer cell autophagy and lapatinib effect on breast cancer cells	[306]
GSK-3 inhibitor	Lithium chloride	In vivo mouse model; in vitro human cell culture	Pancreatic and breast cancers	Enhanced autophagy and apoptosis in cancer cells and reduced cancer growth	[307]
PGAM1 inhibitor	HKB99	In vivo mouse model	NSCLC	Suppressed tumor growth and metastasis	[308]
	PGMI-004A	In vivo mouse model	NSCLC	Attenuated cell proliferation and tumor growth	[309]
ACSLs inhibition	Triacsin C	In vitro human cell culture	Colon and breast cancers	Decreased cell proliferation and chemotherapy resistance	[310, 311]
ACAT-1	Avasimibe	In vitro human cell culture	Ovarian cancer	Enhanced chemosensitivity to cisplatin treatment	[312]
FASN inhibitor	C75	FDA approved	NSCLC	Reduced tumor growth	[313, 314]
	TVB-2640	Phase II	NSCLC, ovarian, and breast cancer	Inhibited cancer growth	NCT02223247 [315]
	Cerulenin	In vitro human cell culture	Lung cancers	Blocked cancer cell proliferation	[316]
HMGCR inhibitor	Fluvastatin	In vivo mouse model	NSCLC	Inhibited cancer growth	[317]
GLS inhibitor	Telaglenastat (CB-839)	In vivo mouse model	Melanoma	Improvement in tumor growth inhibition with anti-PD1 and anti-CTLA4 antibodies	[318]
	Withangulatin A derivative 7	In vivo mouse model	Breast cancer	Inhibited cancer growth	[319]

*GLUT* glucose transporter; *HK II* hexokinase II; *CAV-1* caveolin-1; *BITC* benzyl isothiocyanate; *MCT-1* monocarboxylate transporter 1; *PDK1* pyruvate dehydrogenase kinase 1; *IDH* isocitrate dehydrogenase; *HIF-1* hypoxia-inducible factor-1; *OXPHOS* oxidative phosphorylation; *G6PD* glucose-6-phosphate dehydrogenase; *GSK-3* glycogen synthase kinase; *PGAM* phosphoglycerate mutase; *NSCLC* non-small cell lung cancer; *ACSL* acyl CoA synthetase; *FASN* fatty acid synthase; *HMGCR* 3-hydroxy-3-methylglutaryl-CoA reductase; and *GLS1* glutaminase 1

Second, since cancer-associated stromal and cancer cells are metabolically coupled to support cancer cells, targeting both cancer cells and their adjacent stromal cells within the TME is an attractive therapeutic schedule. Our research group previously proposed the concept of common target perturbation (CTP) [160]. Through a systems biology approach, we found that TGF-βRIII is significantly changed in cancer and verified that TGF-βRIII is downregulated in both cancer cells and fibroblasts. TGF-βRIII was found to be a common epithelial–mesenchymal target in oral squamous cell carcinoma. Simultaneous perturbation of TGF-βRIII in oral cancerous epithelial cells and their adjacent CAFs effectively inhibits tumor growth in vivo and shows superiority to the unilateral perturbation of TβRIII in either cell type alone. Inspired by CTP conception, we proposed the concept of metabolic coupling target perturbation (MCTP). For example, CAV-1-, IDH3α-, and HIF-1α-mediated epithelial–CAF metabolic coupling are epithelial–CAF MCTPs. HK2, as a Warburg effect promoter, also decreases glycolysis in T cells and makes them immune tolerant through glucose competition. Therefore, HK2 is an epithelium-T cell MCTP. Future studies may need to verify the cancer treatment efficacy of these MCPTs. Considering that TGF-β signaling influences the immune, mechanical, and metabolic microenvironment in cancer, effective TGF-β-associated common target is expected to achieve the effect of targeting metabolic microenvironment, mechanical microenvironment, and immune microenvironment together.

Third, combining TGF-β-dependent metabolism-targeted therapy with immunotherapy or conventional cancer therapy may maximize their efficacy. Numerous immunotherapies have been approved for application in cancer patients, with prominent effects on cancer therapy status, among which immune checkpoint inhibitors such as PD-1/PD-L1 blockade are the most widely used therapies. Since the anticancer effects of these immunotherapies are limited, TGF-β therapies usually combine PD-1/PD-L1 blockade to strengthen their efficacy. M7824 is a classical drug that targets both PD-L1 and TGF-β signaling and comprises the recombinant anti-PD-L1 antibody and TGF-βRII fusion protein. However, M7824 failed in multiple Phase III clinical trials, including triple-negative breast cancer and NSCLC. The following reasons might be responsible for the failure. In terms of M7824 drug design, the TGF-βRII structure in M7824 may not capture TGF-β efficiently. We inferred that better outcomes could be achieved if TGF-βRI/TGF-βRII complex was made to mimic the in vitro TGFβ ligand-receptor interactions. Besides, phosphorylation of TGFβRI should be avoided to prevent the amplified TGF-β signaling. As for M7824 patient selection, except for the high expression of PD-L1, patients may also need to satisfy the requirement of TGF-β target therapy, such as the TGF-β activation. This makes the patient selection much stricter. Considering the tumor heterogeneity, not all patients meet these requirements. Moreover, both anti-PD-L1 signaling and TGF-β signaling focus on cancer immunology but not killing cells directly. TGF-β target therapies could combine with treatment that directly kill cancer cells, such as radiotherapy and chemotherapy to achieve better anti-tumor effect. TGF-β target therapy resistance will occur after a period of medication application, and new agents should be used. Metabolic reprogramming influences the effector function of immune cells. Future studies could explore the targeting of TGF-β-regulated metabolic enzymes and their combination with immunotherapy. For example, TGF-β signaling is responsible for Treg transformation by downregulating glycolysis via a decrease in GLUT1 and HK2. Therefore, the GLUT1 and HK2 inhibitors shown in Table 4 could be tested to determine whether they can combine with and increase the efficacy of PD-1/PD-L1 blockade therapy.

Overall, preclinical in vitro and animal model studies have verified that targeting TGF-β efficiently prevents cancer progression by regulating metabolic enzymes via failing the “engine.” TGF-β-dependent metabolism provides far more promising targets that can substitute for TGF-β inhibitors at the level of TGF-β isoforms or their receptors. However, one enzyme usually has isozymes depending on their different structures. Future studies to design agents that specifically target one isozyme are needed to minimize adverse effects and provide potent cancer therapeutic effects.

## Data Availability

Not applicable.

## Abbreviations

TGF-β: Transforming growth factor-β
TME: Tumor microenvironment
TMME: Tumor metabolic microenvironment
EMT: Epithelial–mesenchymal transition
TECs: Tumor endothelial cells
TCA: Tricarboxylic acid
GLUT: Glucose transporter
PDAC: Pancreatic ductal adenocarcinoma
NSCLC: Non-small cell lung cancer
HK2: Hexokinase 2
PFKFB3: 6-phosphofructo-2-kinase/fructose-2,6-bisphosphatase 3
PFK1: 6-Phosphofructo-1-kinase
PKM2: Pyruvate kinase M2
TGIF2: TGF-β-induced factor homeobox 2
PPP: Pentose phosphate pathway
LEFTY2: Endometrial bleeding-associated factor
GSK-3: Glycogen synthase kinase
FAO: Fatty acid oxidation
NSDHL: NADPH steroid dehydrogenase-like protein
SREBF2: Sterol regulatory element-binding transcription factor 2
FASN: Fatty acid synthase
ACSL5: Acyl CoA synthetase 5
PPARγ: Peroxisome proliferator-activated receptor gamma
NFs: Normal fibroblasts
RWE: Reverse Warburg effect
PDK1: Pyruvate dehydrogenase kinase 1
BCAT1: BCAA transaminase 1
BCKAs: Branched-chain α-ketoacids

## References

[1] Yeh HW, Lee SS, Chang CY, Lang YD, Jou YS (2019). A new switch for TGFβ in cancer. Cancer Res.

[2] Davis MD, Suzaki I, Kawano S, Komiya K, Cai Q, Oh Y, Rubin BK (2019). Tissue factor facilitates wound healing in human airway epithelial cells. Chest.

[3] Hinck AP, Mueller TD, Springer TA (2016). Structural biology and evolution of the TGF-β family. Cold Spring Harb Perspect Biol.

[4] Wynn TA, Barron L (2010). Macrophages: master regulators of inflammation and fibrosis. Semin Liver Dis.

[5] Batlle E, Massagué J (2019). Transforming growth factor-β signaling in immunity and cancer. Immunity.

[6] Xu X, Zheng L, Yuan Q, Zhen G, Crane JL, Zhou X, Cao X (2018). Transforming growth factor-β in stem cells and tissue homeostasis. Bone research.

[7] Kaplan DH, Li MO, Jenison MC, Shlomchik WD, Flavell RA, Shlomchik MJ (2007). Autocrine/paracrine TGFbeta1 is required for the development of epidermal langerhans cells. J Exp Med.

[8] Wu F, Weigel KJ, Zhou H, Wang XJ (2018). Paradoxical roles of TGF-β signaling in suppressing and promoting squamous cell carcinoma. Acta Biochim Biophys Sin.

[9] Liu S, Ren J, Ten Dijke P (2021). Targeting TGFβ signal transduction for cancer therapy. Signal Transduct Target Ther.

[10] Shi X, Young CD, Zhou H, Wang X (2020). Transforming growth factor-β signaling in fibrotic diseases and cancer-associated fibroblasts. Biomolecules.

[11] Seoane J, Gomis RR (2017). TGF-β family signaling in tumor suppression and cancer progression. Cold Spring Harb Perspect Biol.

[12] Bagati A, Kumar S, Jiang P, Pyrdol J, Zou AE, Godicelj A, Mathewson ND, Cartwright ANR, Cejas P, Brown M (2021). Integrin αvβ6-TGFβ-SOX4 pathway drives immune evasion in triple-negative breast cancer. Cancer Cell.

[13] Lin YT, Wu KJ (2020). Epigenetic regulation of epithelial-mesenchymal transition: focusing on hypoxia and TGF-β signaling. J Biomed Sci.

[14] Faubert B, Solmonson A, de Berardinis RJ (2020). Metabolic reprogramming and cancer progression. Science.

[15] Seyfried TN (2015). Cancer as a mitochondrial metabolic disease. Front Cell Dev Biol.

[16] Guido C, Whitaker-Menezes D, Capparelli C, Balliet R, Lin Z, Pestell RG, Howell A, Aquila S, Andò S, Martinez-Outschoorn U (2012). Metabolic reprogramming of cancer-associated fibroblasts by TGF-β drives tumor growth: connecting TGF-β signaling with "Warburg-like" cancer metabolism and L-lactate production. Cell Cycle.

[17] Fosslien E (2008). Cancer morphogenesis: role of mitochondrial failure. Ann Clin Lab Sci.

[18] Wang YA, Li XL, Mo YZ, Fan CM, Tang L, Xiong F, Guo C, Xiang B, Zhou M, Ma J (2018). Effects of tumor metabolic microenvironment on regulatory T cells. Mol Cancer.

[19] García-Cañaveras JC, Chen L, Rabinowitz JD (2019). The tumor metabolic microenvironment: lessons from lactate. Cancer Res.

[20] Eisenberg L, Eisenberg-Bord M, Eisenberg-Lerner A, Sagi-Eisenberg R (2020). Metabolic alterations in the tumor microenvironment and their role in oncogenesis. Cancer Lett.

[21] Xiao Z, Dai Z, Locasale JW (2019). Metabolic landscape of the tumor microenvironment at single cell resolution. Nat Commun.

[22] Magalhaes I, Yogev O, Mattsson J, Schurich A (2019). The metabolic profile of tumor and virally infected cells shapes their microenvironment counteracting T Cell immunity. Front Immunol.

[23] Yang E, Wang X, Gong Z, Yu M, Wu H, Zhang D (2020). Exosome-mediated metabolic reprogramming: the emerging role in tumor microenvironment remodeling and its influence on cancer progression. Signal Transduct Target Ther.

[24] Li X, Wenes M, Romero P, Huang SC, Fendt SM, Ho PC (2019). Navigating metabolic pathways to enhance antitumour immunity and immunotherapy. Nat Rev Clin Oncol.

[25] Boroughs LK, DeBerardinis RJ (2015). Metabolic pathways promoting cancer cell survival and growth. Nat Cell Biol.

[26] O'Neill LA, Pearce EJ (2016). Immunometabolism governs dendritic cell and macrophage function. J Exp Med.

[27] Hua W, Ten Dijke P, Kostidis S, Giera M, Hornsveld M (2020). TGFβ-induced metabolic reprogramming during epithelial-to-mesenchymal transition in cancer. Cellular Mol Life Sci: CMLS.

[28] Ye J, Medzhitov R (2019). Control strategies in systemic metabolism. Nat Metab.

[29] Ocaña MC, Martínez-Poveda B, Quesada AR, Medina M (2019). Metabolism within the tumor microenvironment and its implication on cancer progression: an ongoing therapeutic target. Med Res Rev.

[30] Egeblad M, Nakasone ES, Werb Z (2010). Tumors as organs: complex tissues that interface with the entire organism. Dev Cell.

[31] Judge A, Dodd MS (2020). Metabolism. Essays Biochem.

[32] Zhu J, Thompson CB (2019). Metabolic regulation of cell growth and proliferation. Nat Rev Mol Cell Biol.

[33] Deng S, Wang S, Shi X, Zhou H (2022). Microenvironment in oral potentially malignant disorders: multi-dimensional characteristics and mechanisms of carcinogenesis. Int J Mol Sci.

[34] Koundouros N, Poulogiannis G (2020). Reprogramming of fatty acid metabolism in cancer. Br J Cancer.

[35] Kimmelman AC, White E (2017). Autophagy and tumor metabolism. Cell Metab.

[36] Gupta S, Roy A, Dwarakanath BS (2017). Metabolic cooperation and competition in the tumor microenvironment: implications for therapy. Front Oncol.

[37] Shen W, Tao G-Q, Zhang Y, Cai B, Sun J, Tian Z-Q (2017). TGF-β in pancreatic cancer initiation and progression: two sides of the same coin. Cell Biosci.

[38] Penafuerte C, Bautista-Lopez N, Bouchentouf M, Birman E, Forner K, Galipeau J (2011). Novel TGF-β antagonist inhibits tumor growth and angiogenesis by inducing IL-2 receptor-driven STAT1 activation. J Immunol.

[39] Xie F, Ling L, van Dam H, Zhou F, Zhang L (2018). TGF-β signaling in cancer metastasis. Acta Biochim Biophys Sin.

[40] Shi X, Luo J, Weigel KJ, Hall SC, Du D, Wu F, Rudolph MC, Zhou H, Young CD, Wang X-J (2021). Cancer-associated fibroblasts facilitate squamous cell carcinoma lung metastasis in mice by providing TGFβ-mediated cancer stem cell niche. Front Cell Dev Biol.

[41] Angioni R, Sánchez-Rodríguez R, Viola A, Molon B (2021). TGF-β in cancer: metabolic driver of the tolerogenic crosstalk in the tumor microenvironment. Cancers.

[42] Warburg O (1956). On the origin of cancer cells. Science.

[43] Weinhouse S (1972). Glycolysis, respiration, and anomalous gene expression in experimental hepatomas: G.H.A Clowes memorial lecture. Cancer Res.

[44] Weinhouse S (1955). Oxidative metabolism of neoplastic tissues. Adv Cancer Res.

[45] Gatenby RA, Gillies RJ (2004). Why do cancers have high aerobic glycolysis?. Nat Rev Cancer.

[46] Vander Heiden MG, Cantley LC, Thompson CBJ (2009). Understanding the Warburg effect: the metabolic requirements of cell proliferation. Science.

[47] Lunt SY, Vander Heiden MG (2011). Aerobic glycolysis: meeting the metabolic requirements of cell proliferation. Annu Rev Cell Dev Biol.

[48] de la Cruz-López KG, Castro-Muñoz LJ, Reyes-Hernández DO, García-Carrancá A, Manzo-Merino J (2019). Lactate in the regulation of tumor microenvironment and therapeutic approaches. Front Oncol.

[49] Bardella C, Pollard PJ, Tomlinson I (2011). SDH mutations in cancer. Biochem Biophys Acta.

[50] Letouzé E, Martinelli C, Loriot C, Burnichon N, Abermil N, Ottolenghi C, Janin M, Menara M, Nguyen AT, Benit P (2013). SDH mutations establish a hypermethylator phenotype in paraganglioma. Cancer Cell.

[51] Marquez J, Flores J, Kim AH, Nyamaa B, Nguyen ATT, Park N, Han J (2019). Rescue of TCA cycle dysfunction for cancer therapy. J Clin Med.

[52] Patra KC, Hay N (2014). The pentose phosphate pathway and cancer. Trends Biochem Sci.

[53] Jiang P, Du W, Wu M (2014). Regulation of the pentose phosphate pathway in cancer. Protein Cell.

[54] Feng Q, Li X, Sun W, Sun M, Li Z, Sheng H, Xie F, Zhang S, Shan C (2020). Targeting G6PD reverses paclitaxel resistance in ovarian cancer by suppressing GSTP1. Biochem Pharmacol.

[55] Zhang Q, Han Q, Yang Z, Ni Y, Agbana YL, Bai H, Yi Z, Yi X, Kuang Y, Zhu Y (2020). G6PD facilitates clear cell renal cell carcinoma invasion by enhancing MMP2 expression through ROS-MAPK axis pathway. Int J Oncol.

[56] Yamawaki K, Mori Y, Sakai H, Kanda Y, Shiokawa D, Ueda H, Ishiguro T, Yoshihara K, Nagasaka K, Onda T (2021). Integrative analyses of gene expression and chemosensitivity of patient-derived ovarian cancer spheroids link G6PD-driven redox metabolism to cisplatin chemoresistance. Cancer Lett.

[57] Hong W, Cai P, Xu C, Cao D, Yu W, Zhao Z, Huang M, Jin J (2018). Inhibition of glucose-6-phosphate dehydrogenase reverses cisplatin resistance in lung cancer cells via the redox system. Front Pharmacol.

[58] Schmidt M, Voelker HU, Kapp M, Krockenberger M, Dietl J, Kammerer U (2010). Glycolytic phenotype in breast cancer: activation of Akt, up-regulation of GLUT1, TKTL1 and down-regulation of M2PK. J Cancer Res Clin Oncol.

[59] da Costa IA, Hennenlotter J, Stühler V, Kühs U, Scharpf M, Todenhöfer T, Stenzl A, Bedke J (2018). Transketolase like 1 (TKTL1) expression alterations in prostate cancer tumorigenesis. Urol Oncol.

[60] Schultz H, Kähler D, Branscheid D, Vollmer E, Zabel P, Goldmann T (2008). TKTL1 is overexpressed in a large portion of non-small cell lung cancer specimens. Diagn Pathol.

[61] Wang C, Guo K, Gao D, Kang X, Jiang K, Li Y, Sun L, Zhang S, Sun C, Liu X (2011). Identification of transaldolase as a novel serum biomarker for hepatocellular carcinoma metastasis using xenografted mouse model and clinic samples. Cancer Lett.

[62] Du W, Zhang L, Brett-Morris A, Aguila B, Kerner J, Hoppel CL, Puchowicz M, Serra D, Herrero L, Rini BI (2017). HIF drives lipid deposition and cancer in ccRCC via repression of fatty acid metabolism. Nat Commun.

[63] Gounaris I, Brenton JD (2015). Molecular pathogenesis of ovarian clear cell carcinoma. Future Oncol.

[64] Pletneva MA, Andea A, Palanisamy N, Betz BL, Carskadon S, Wang M, Patel RM, Fullen DR, Harms PW (2014). Clear cell melanoma: a cutaneous clear cell malignancy. Arch Pathol Lab Med.

[65] Pescador N, Villar D, Cifuentes D, Garcia-Rocha M, Ortiz-Barahona A, Vazquez S, Ordoñez A, Cuevas Y, Saez-Morales D, Garcia-Bermejo ML (2010). Hypoxia promotes glycogen accumulation through hypoxia inducible factor (HIF)-mediated induction of glycogen synthase 1. PLoS ONE.

[66] Shen GM, Zhang FL, Liu XL, Zhang JW (2010). Hypoxia-inducible factor 1-mediated regulation of PPP1R3C promotes glycogen accumulation in human MCF-7 cells under hypoxia. FEBS Lett.

[67] Pelletier J, Bellot G, Gounon P, Lacas-Gervais S, Pouysségur J, Mazure NM (2012). Glycogen synthesis is induced in hypoxia by the hypoxia-inducible factor and promotes cancer cell survival. Front Oncol.

[68] Iida Y, Aoki K, Asakura T, Ueda K, Yanaihara N, Takakura S, Yamada K, Okamoto A, Tanaka T, Ohkawa K (2012). Hypoxia promotes glycogen synthesis and accumulation in human ovarian clear cell carcinoma. Int J Oncol.

[69] Dauer P, Lengyel E (2019). New roles for glycogen in tumor progression. Trends Cancer.

[70] Liu Q, Li J, Zhang W, Xiao C, Zhang S, Nian C, Li J, Su D, Chen L, Zhao Q (2021). Glycogen accumulation and phase separation drives liver tumor initiation. Cell.

[71] Favaro E, Bensaad K, Chong MG, Tennant DA, Ferguson DJ, Snell C, Steers G, Turley H, Li JL, Günther UL (2012). Glucose utilization via glycogen phosphorylase sustains proliferation and prevents premature senescence in cancer cells. Cell Metab.

[72] Pastushenko I, Blanpain C (2019). EMT transition states during tumor progression and metastasis. Trends Cell Biol.

[73] Xue G, Restuccia DF, Lan Q, Hynx D, Dirnhofer S, Hess D, Rüegg C, Hemmings BA (2012). Akt/PKB-mediated phosphorylation of Twist1 promotes tumor metastasis via mediating cross-talk between PI3K/Akt and TGF-β signaling axes. Cancer Discov.

[74] Brandl M, Seidler B, Haller F, Adamski J, Schmid RM, Saur D, Schneider G (2010). IKK(α) controls canonical TGF(ß)-SMAD signaling to regulate genes expressing SNAIL and SLUG during EMT in panc1 cells. J Cell Sci.

[75] Zhang L, Wang X, Lai C, Zhang H, Lai M (2019). PMEPA1 induces EMT via a non-canonical TGF-β signalling in colorectal cancer. J Cell Mol Med.

[76] Li W, Wei Z, Liu Y, Li H, Ren R, Tang Y (2010). Increased 18F-FDG uptake and expression of Glut1 in the EMT transformed breast cancer cells induced by TGF-beta. Neoplasma.

[77] Liu M, Quek LE, Sultani G, Turner N (2016). Epithelial-mesenchymal transition induction is associated with augmented glucose uptake and lactate production in pancreatic ductal adenocarcinoma. Cancer Metab.

[78] Nilchian A, Giotopoulou N, Sun W, Fuxe J (2020). Different regulation of Glut1 expression and glucose uptake during the induction and chronic stages of TGFβ1-induced EMT in breast cancer cells. Biomolecules.

[79] Dai H, Deng HB, Wang YH, Guo JJ (2018). Resveratrol inhibits the growth of gastric cancer via the Wnt/β-catenin pathway. Oncol Lett.

[80] Oh S, Kim H, Nam K, Shin I (2017). Silencing of Glut1 induces chemoresistance via modulation of Akt/GSK-3β/β-catenin/survivin signaling pathway in breast cancer cells. Arch Biochem Biophys.

[81] Masin M, Vazquez J, Rossi S, Groeneveld S, Samson N, Schwalie PC, Deplancke B, Frawley LE, Gouttenoire J, Moradpour D (2014). GLUT3 is induced during epithelial-mesenchymal transition and promotes tumor cell proliferation in non-small cell lung cancer. Cancer Metab.

[82] Botzer LE, Maman S, Sagi-Assif O, Meshel T, Nevo I, Yron I, Witz IP (2016). Hexokinase 2 is a determinant of neuroblastoma metastasis. Br J Cancer.

[83] Chen J, Yu Y, Li H, Hu Q, Chen X, He Y, Xue C, Ren F, Ren Z, Li J (2019). Long non-coding RNA PVT1 promotes tumor progression by regulating the miR-143/HK2 axis in gallbladder cancer. Mol Cancer.

[84] Rodríguez-García A, Samsó P, Fontova P, Simon-Molas H, Manzano A, Castaño E, Rosa JL, Martinez-Outshoorn U, Ventura F, Navarro-Sabaté À (2017). TGF-β1 targets Smad, p38 MAPK, and PI3K/Akt signaling pathways to induce PFKFB3 gene expression and glycolysis in glioblastoma cells. FEBS J.

[85] De Bock K, Georgiadou M, Schoors S, Kuchnio A, Wong BW, Cantelmo AR, Quaegebeur A, Ghesquière B, Cauwenberghs S, Eelen G (2013). Role of PFKFB3-driven glycolysis in vessel sprouting. Cell.

[86] Van Schaftingen E, Lederer B, Bartrons R, Hers HG (1982). A kinetic study of pyrophosphate: fructose-6-phosphate phosphotransferase from potato tubers. Application to a microassay of fructose 2,6-bisphosphate. Eur J Biochem.

[87] Yalcin A, Solakoglu TH, Ozcan SC, Guzel S, Peker S, Celikler S, Balaban BD, Sevinc E, Gurpinar Y, Chesney JA (2017). 6-phosphofructo-2-kinase/fructose 2,6-bisphosphatase-3 is required for transforming growth factor β1-enhanced invasion of Panc1 cells in vitro. Biochem Biophys Res Commun.

[88] Zahra K, Dey T, Ashish, Mishra SP, Pandey U (2020). Pyruvate Kinase M2 and Cancer: the role of PKM2 in promoting tumorigenesis. Front Oncol.

[89] Hamabe A, Konno M, Tanuma N, Shima H, Tsunekuni K, Kawamoto K, Nishida N, Koseki J, Mimori K, Gotoh N (2014). Role of pyruvate kinase M2 in transcriptional regulation leading to epithelial-mesenchymal transition. Proc Natl Acad Sci USA.

[90] Liu Y, Yuan X, Li W, Cao Q, Shu Y (2016). Aspirin-triggered resolvin D1 inhibits TGF-β1-induced EMT through the inhibition of the mTOR pathway by reducing the expression of PKM2 and is closely linked to oxidative stress. Int J Mol Med.

[91] Vander Heiden MG, Christofk HR, Schuman E, Subtelny AO, Sharfi H, Harlow EE, Xian J, Cantley LC (2010). Identification of small molecule inhibitors of pyruvate kinase M2. Biochem Pharmacol.

[92] Chen J, Xie J, Jiang Z, Wang B, Wang Y, Hu X (2011). Shikonin and its analogs inhibit cancer cell glycolysis by targeting tumor pyruvate kinase-M2. Oncogene.

[93] Shankar Babu M, Mahanta S, Lakhter AJ, Hato T, Paul S, Naidu SR (2018). Lapachol inhibits glycolysis in cancer cells by targeting pyruvate kinase M2. PLoS ONE.

[94] Li X, Zhang Z, Zhang Y, Cao Y, Wei H, Wu Z (2018). Upregulation of lactate-inducible snail protein suppresses oncogene-mediated senescence through p16(INK4a) inactivation. J Exp Clin Cancer Res: CR.

[95] Fischer K, Hoffmann P, Voelkl S, Meidenbauer N, Ammer J, Edinger M, Gottfried E, Schwarz S, Rothe G, Hoves S (2007). Inhibitory effect of tumor cell-derived lactic acid on human T cells. Blood.

[96] Wang H, Chen Y, Wu G (2016). SDHB deficiency promotes TGFβ-mediated invasion and metastasis of colorectal cancer through transcriptional repression complex SNAIL1-SMAD3/4. Transl oncology.

[97] Aspuria PP, Lunt SY, Väremo L, Vergnes L, Gozo M, Beach JA, Salumbides B, Reue K, Wiedemeyer WR, Nielsen J (2014). Succinate dehydrogenase inhibition leads to epithelial-mesenchymal transition and reprogrammed carbon metabolism. Cancer Metabol.

[98] Soukupova J, Malfettone A, Hyroššová P, Hernández-Alvarez M-I, Peñuelas-Haro I, Bertran E, Junza A, Capellades J, Giannelli G, Yanes O (2017). Role of the transforming growth factor-β in regulating hepatocellular carcinoma oxidative metabolism. Sci Rep.

[99] Sun W, Ma Y, Chen P, Wang D (2015). MicroRNA-10a silencing reverses cisplatin resistance in the A549/cisplatin human lung cancer cell line via the transforming growth factor-β/Smad2/STAT3/STAT5 pathway. Mol Med Rep.

[100] Bissey P-A, Law JH, Bruce JP, Shi W, Renoult A, Chua MLK, Yip KW, Liu F-F (2018). Dysregulation of the MiR-449b target TGFBI alters the TGFβ pathway to induce cisplatin resistance in nasopharyngeal carcinoma. Oncogenesis.

[101] Zhang R, Tao F, Ruan S, Hu M, Hu Y, Fang Z, Mei L, Gong C (2019). The TGFβ1-FOXM1-HMGA1-TGFβ1 positive feedback loop increases the cisplatin resistance of non-small cell lung cancer by inducing G6PD expression. Am J Transl Res.

[102] Zeng N, Okumura T, Alauddin M, Khozooei S, Rajaxavier J, Zhang S, Singh Y, Shi B, Brucker SY, Wallwiener D (2020). LEFTY2/endometrial bleeding-associated factor up-regulates Na+ coupled glucose transporter SGLT1 expression and glycogen accumulation in endometrial cancer cells. PLoS ONE.

[103] Guo X, Ramirez A, Waddell DS, Li Z, Liu X, Wang XF (2008). Axin and GSK3- control Smad3 protein stability and modulate TGF- signaling. Genes Dev.

[104] Cozzolino AM, Alonzi T, Santangelo L, Mancone C, Conti B, Steindler C, Musone M, Cicchini C, Tripodi M, Marchetti A (2013). TGFβ overrides HNF4α tumor suppressing activity through GSK3β inactivation: implication for hepatocellular carcinoma gene therapy. J Hepatol.

[105] Matsumoto T, Yokoi A, Hashimura M, Oguri Y, Akiya M, Saegusa M (2018). TGF-β-mediated LEFTY/Akt/GSK-3β/Snail axis modulates epithelial-mesenchymal transition and cancer stem cell properties in ovarian clear cell carcinomas. Mol Carcinog.

[106] Goluszko P, Nowicki B (2005). Membrane cholesterol: a crucial molecule affecting interactions of microbial pathogens with mammalian cells. Infect Immun.

[107] Zhao W, Prijic S, Urban BC, Tisza MJ, Zuo Y, Li L, Tan Z, Chen X, Mani SA, Chang JT (2016). Candidate antimetastasis drugs suppress the metastatic capacity of breast cancer cells by reducing membrane fluidity. Can Res.

[108] Baek AE, Yu YA, He S, Wardell SE, Chang CY, Kwon S, Pillai RV, McDowell HB, Thompson JW, Dubois LG (2017). The cholesterol metabolite 27 hydroxycholesterol facilitates breast cancer metastasis through its actions on immune cells. Nat Commun.

[109] Röhrig F, Schulze A (2016). The multifaceted roles of fatty acid synthesis in cancer. Nat Rev Cancer.

[110] Menendez JA, Lupu R (2017). Fatty acid synthase (FASN) as a therapeutic target in breast cancer. Expert Opin Ther Targets.

[111] Tirinato L, Pagliari F, Limongi T, Marini M, Falqui A, Seco J, Candeloro P, Liberale C, Di Fabrizio E (2017). An overview of lipid droplets in cancer and cancer stem cells. Stem Cells Int.

[112] Bensaad K, Favaro E, Lewis CA, Peck B, Lord S, Collins JM, Pinnick KE, Wigfield S, Buffa FM, Li JL (2014). Fatty acid uptake and lipid storage induced by HIF-1α contribute to cell growth and survival after hypoxia-reoxygenation. Cell Rep.

[113] Flavin R, Peluso S, Nguyen PL, Loda M (2010). Fatty acid synthase as a potential therapeutic target in cancer. Future Oncol.

[114] Xu S, Chen T, Dong L, Li T, Xue H, Gao B, Ding X, Wang H, Li H (2021). Fatty acid synthase promotes breast cancer metastasis by mediating changes in fatty acid metabolism. Oncol Lett.

[115] Aiderus A, Black MA, Dunbier AK (2018). Fatty acid oxidation is associated with proliferation and prognosis in breast and other cancers. BMC Cancer.

[116] Mozolewska P, Duzowska K, Pakiet A, Mika A, ŚledziŃski T (2020). Inhibitors of fatty acid synthesis and oxidation as potential anticancer agents in colorectal cancer treatment. Anticancer Res.

[117] Chen M, Zhao Y, Yang X, Zhao Y, Liu Q, Liu Y, Hou Y, Sun H, Jin W (2021). NSDHL promotes triple-negative breast cancer metastasis through the TGFβ signaling pathway and cholesterol biosynthesis. Breast Cancer Res Treat.

[118] Di Guglielmo GM, Le Roy C, Goodfellow AF, Wrana JL (2003). Distinct endocytic pathways regulate TGF-beta receptor signalling and turnover. Nat Cell Biol.

[119] Hayes S, Chawla A, Corvera S (2002). TGF beta receptor internalization into EEA1-enriched early endosomes: role in signaling to Smad2. J Cell Biol.

[120] Chen YG (2009). Endocytic regulation of TGF-beta signaling. Cell Res.

[121] Gabitova-Cornell L, Surumbayeva A, Peri S, Franco-Barraza J, Restifo D, Weitz N, Ogier C, Goldman AR, Hartman TR, Francescone R (2020). Cholesterol pathway inhibition induces TGF-β signaling to promote Basal differentiation in pancreatic cancer. Cancer Cell.

[122] Chen C-L, Huang SS, Huang JS (2008). Cholesterol modulates cellular TGF-beta responsiveness by altering TGF-beta binding to TGF-beta receptors. J Cell Physiol.

[123] Zhao Z, Hao D, Wang L, Li J, Meng Y, Li P, Wang Y, Zhang C, Zhou H, Gardner K (2019). CtBP promotes metastasis of breast cancer through repressing cholesterol and activating TGF-β signaling. Oncogene.

[124] Xue L, Qi H, Zhang H, Ding L, Huang Q, Zhao D, Wu BJ, Li X (2020). Targeting SREBP-2-regulated mevalonate metabolism for cancer therapy. Front Oncol.

[125] Mullen GE, Yet L (2015). Progress in the development of fatty acid synthase inhibitors as anticancer targets. Bioorg Med Chem Lett.

[126] Yang L, Zhang F, Wang X, Tsai Y, Chuang K-H, Keng PC, Lee SO, Chen Y (2016). A FASN-TGF-β1-FASN regulatory loop contributes to high EMT/metastatic potential of cisplatin-resistant non-small cell lung cancer. Oncotarget.

[127] Liu QQ, Huo HY, Ao S, Liu T, Yang L, Fei ZY, Zhang ZQ, Ding L, Cui QH, Lin J (2020). TGF-β1-induced epithelial-mesenchymal transition increases fatty acid oxidation and OXPHOS activity via the p-AMPK pathway in breast cancer cells. Oncol Rep.

[128] Damaghi M, Gillies R (2017). Phenotypic changes of acid-adapted cancer cells push them toward aggressiveness in their evolution in the tumor microenvironment. Cell Cycle.

[129] Corbet C, Feron O (2017). Tumour acidosis: from the passenger to the driver's seat. Nat Rev Cancer.

[130] Olzmann JA, Carvalho P (2019). Dynamics and functions of lipid droplets. Nat Rev Mol Cell Biol.

[131] Corbet C, Bastien E, de Santiago Jesus JP, Dierge E, Martherus R, Vander Linden C, Doix B, Degavre C, Guilbaud C, Petit L (2020). TGFβ2-induced formation of lipid droplets supports acidosis-driven EMT and the metastatic spreading of cancer cells. Nat Commun.

[132] Vučetić M, Cormerais Y, Parks SK, Pouysségur J (2017). The central role of amino acids in cancer redox homeostasis: vulnerability points of the cancer redox code. Front Oncol.

[133] Matés JM, Pérez-Gómez C, de Núñez CI, Asenjo M, Márquez J (2002). Glutamine and its relationship with intracellular redox status, oxidative stress and cell proliferation/death. Int J Biochem Cell Biol.

[134] Li Z, Zhang H (2016). Reprogramming of glucose, fatty acid and amino acid metabolism for cancer progression. Cell Mol Life Sci: CMLS.

[135] Togashi Y, Arao T, Kato H, Matsumoto K, Terashima M, Hayashi H, de Velasco MA, Fujita Y, Kimura H, Yasuda T (2014). Frequent amplification of ORAOV1 gene in esophageal squamous cell cancer promotes an aggressive phenotype via proline metabolism and ROS production. Oncotarget.

[136] Scott L, Lamb J, Smith S, Wheatley DN (2000). Single amino acid (arginine) deprivation: rapid and selective death of cultured transformed and malignant cells. Br J Cancer.

[137] Nakasuka F, Tabata S, Sakamoto T, Hirayama A, Ebi H, Yamada T, Umetsu K, Ohishi M, Ueno A, Goto H (2021). TGF-β-dependent reprogramming of amino acid metabolism induces epithelial–mesenchymal transition in non-small cell lung cancers. Commun Biol.

[138] Kinugasa H, Whelan KA, Tanaka K, Natsuizaka M, Long A, Guo A, Chang S, Kagawa S, Srinivasan S, Guha M (2015). Mitochondrial SOD2 regulates epithelial–mesenchymal transition and cell populations defined by differential CD44 expression. Oncogene.

[139] Hibbs JB, Taintor RR, Vavrin Z (1987). Macrophage cytotoxicity: role for L-arginine deiminase and imino nitrogen oxidation to nitrite. Science.

[140] Xie K, Dong Z, Fidler IJ (1996). Activation of nitric oxide synthase gene for inhibition of cancer metastasis. J Leukoc Biol.

[141] Lejeune P, Lagadec P, Onier N, Pinard D, Ohshima H, Jeannin JF (1994). Nitric oxide involvement in tumor-induced immunosuppression. J Immunol (Baltimore, Md: 1950).

[142] Lagadec P, Raynal S, Lieubeau B, Onier N, Arnould L, Saint-Giorgio V, Lawrence DA, Jeannin JF (1999). Evidence for control of nitric oxide synthesis by intracellular transforming growth factor-beta1 in tumor cells. Implications for tumor development. Am J Pathol.

[143] Wu F, Yang J, Liu J, Wang Y, Mu J, Zeng Q, Deng S, Zhou H (2021). Signaling pathways in cancer-associated fibroblasts and targeted therapy for cancer. Signal Transduct Target Ther.

[144] Hawinkels LJ, Paauwe M, Verspaget HW, Wiercinska E, van der Zon JM, van der Ploeg K, Koelink PJ, Lindeman JH, Mesker W, ten Dijke P (2014). Interaction with colon cancer cells hyperactivates TGF-β signaling in cancer-associated fibroblasts. Oncogene.

[145] Ringuette Goulet C, Bernard G, Tremblay S, Chabaud S, Bolduc S, Pouliot F (2018). Exosomes induce fibroblast differentiation into cancer-associated fibroblasts through TGFβ signaling. Mol Cancer Res: MCR.

[146] Untergasser G, Gander R, Lilg C, Lepperdinger G, Plas E, Berger P (2005). Profiling molecular targets of TGF-beta1 in prostate fibroblast-to-myofibroblast transdifferentiation. Mech Ageing Dev.

[147] Lamprecht S, Sigal-Batikoff I, Shany S, Abu-Freha N, Ling E, Delinasios GJ, Moyal-Atias K, Delinasios JG, Fich A (2018). Teaming up for trouble: cancer cells, transforming growth factor-β1 signaling and the epigenetic corruption of stromal naïve fibroblasts. Cancers.

[148] Lin J, Liu C, Ge L, Gao Q, He X, Liu Y, Li S, Zhou M, Chen Q, Zhou H (2011). Carcinoma-associated fibroblasts promotes the proliferation of a lingual carcinoma cell line by secreting keratinocyte growth factor. Tumour Biol.

[149] Shi X, Luo J, Weigel KJ, Hall SC, Du D, Wu F, Rudolph MC, Zhou H, Young CD, Wang XJ (2021). Cancer-associated fibroblasts facilitate squamous cell carcinoma lung metastasis in Mice by providing TGFβ-mediated cancer stem cell Niche. Front Cell Dev Biol.

[150] Zhang D, Wang Y, Shi Z, Liu J, Sun P, Hou X, Zhang J, Zhao S, Zhou BP, Mi J (2015). Metabolic reprogramming of cancer-associated fibroblasts by IDH3α downregulation. Cell Rep.

[151] Roy A, Bera S (2016). CAF cellular glycolysis: linking cancer cells with the microenvironment. Tumour Biol.

[152] Pavlides S, Whitaker-Menezes D, Castello-Cros R, Flomenberg N, Witkiewicz AK, Frank PG, Casimiro MC, Wang C, Fortina P, Addya S (2009). The reverse Warburg effect: aerobic glycolysis in cancer associated fibroblasts and the tumor stroma. Cell Cycle.

[153] Karuppagounder SS, Ratan RR (2012). Hypoxia-inducible factor prolyl hydroxylase inhibition: robust new target or another big bust for stroke therapeutics?. J Cereb Blood Flow Metab.

[154] Berra E, Benizri E, Ginouvès A, Volmat V, Roux D, Pouysségur J (2003). HIF prolyl-hydroxylase 2 is the key oxygen sensor setting low steady-state levels of HIF-1alpha in normoxia. EMBO J.

[155] Ke Q, Costa M (2006). Hypoxia-inducible factor-1 (HIF-1). Mol Pharmacol.

[156] Liu Y, Hu T, Shen J, Li SF, Lin JW, Zheng XH, Gao QH, Zhou HM (2006). Separation, cultivation and biological characteristics of oral carcinoma-associated fibroblasts. Oral Dis.

[157] Wu F, Wang S, Zeng Q, Liu J, Yang J, Mu J, Xu H, Wu L, Gao Q, He X (2022). TGF-βRII regulates glucose metabolism in oral cancer-associated fibroblasts via promoting PKM2 nuclear translocation. Cell death discovery.

[158] Yang J, Shi X, Yang M, Luo J, Gao Q, Wang X, Wu Y, Tian Y, Wu F, Zhou H (2021). Glycolysis reprogramming in cancer-associated fibroblasts promotes the growth of oral cancer through the lncRNA H19/miR-675-5p/PFKFB3 signaling pathway. Int J Oral Sci.

[159] Gong J, Lin Y, Zhang H, Liu C, Cheng Z, Yang X, Zhang J, Xiao Y, Sang N, Qian X (2020). Reprogramming of lipid metabolism in cancer-associated fibroblasts potentiates migration of colorectal cancer cells. Cell Death Dis.

[160] Meng W, Wu Y, He X, Liu C, Gao Q, Ge L, Wu L, Liu Y, Guo Y, Li X (2014). A systems biology approach identifies effective tumor-stroma common targets for oral squamous cell carcinoma. Can Res.

[161] Li Z, Sun C, Qin Z (2021). Metabolic reprogramming of cancer-associated fibroblasts and its effect on cancer cell reprogramming. Theranostics.

[162] Bonuccelli G, Tsirigos A, Whitaker-Menezes D, Pavlides S, Pestell RG, Chiavarina B, Frank PG, Flomenberg N, Howell A, Martinez-Outschoorn UE (2010). Ketones and lactate "fuel" tumor growth and metastasis: evidence that epithelial cancer cells use oxidative mitochondrial metabolism. Cell Cycle.

[163] Hu L, Xu X, Li Q, Chen X, Yuan X, Qiu S, Yao C, Zhang D, Wang F (2021). Caveolin-1 increases glycolysis in pancreatic cancer cells and triggers cachectic states. FASEB J.

[164] Yang L, Achreja A, Yeung TL, Mangala LS, Jiang D, Han C, Baddour J, Marini JC, Ni J, Nakahara R (2016). Targeting stromal glutamine synthetase in tumors disrupts tumor microenvironment-regulated cancer cell growth. Cell Metab.

[165] Mestre-Farrera A, Bruch-Oms M, Peña R, Rodríguez-Morató J, Alba-Castellón L, Comerma L, Quintela-Fandino M, Duñach M, Baulida J, Pozo ÓJ (2021). Glutamine-directed migration of cancer-activated fibroblasts facilitates epithelial tumor invasion. Can Res.

[166] Martinez-Outschoorn UE, Pavlides S, Howell A, Pestell RG, Tanowitz HB, Sotgia F, Lisanti MP (2011). Stromal-epithelial metabolic coupling in cancer: integrating autophagy and metabolism in the tumor microenvironment. Int J Biochem Cell Biol.

[167] Avagliano A, Granato G, Ruocco MR, Romano V, Belviso I, Carfora A, Montagnani S, Arcucci A (2018). Metabolic reprogramming of cancer associated fibroblasts: the slavery of stromal fibroblasts. Biomed Res Int.

[168] Hu JW, Sun P, Zhang DX, Xiong WJ, Mi J (2014). Hexokinase 2 regulates G1/S checkpoint through CDK2 in cancer-associated fibroblasts. Cell Signal.

[169] Papandreou I, Cairns RA, Fontana L, Lim AL, Denko NC (2006). HIF-1 mediates adaptation to hypoxia by actively downregulating mitochondrial oxygen consumption. Cell Metab.

[170] Kim JW, Tchernyshyov I, Semenza GL, Dang CV (2006). HIF-1-mediated expression of pyruvate dehydrogenase kinase: a metabolic switch required for cellular adaptation to hypoxia. Cell Metab.

[171] Meng W, Xia Q, Wu L, Chen S, He X, Zhang L, Gao Q, Zhou H (2011). Downregulation of TGF-beta receptor types II and III in oral squamous cell carcinoma and oral carcinoma-associated fibroblasts. BMC Cancer.

[172] Martinez-Outschoorn UE, Lin Z, Trimmer C, Flomenberg N, Wang C, Pavlides S, Pestell RG, Howell A, Sotgia F, Lisanti MP (2011). Cancer cells metabolically "fertilize" the tumor microenvironment with hydrogen peroxide, driving the Warburg effect: implications for PET imaging of human tumors. Cell Cycle.

[173] Martinez-Outschoorn UE, Balliet RM, Rivadeneira DB, Chiavarina B, Pavlides S, Wang C, Whitaker-Menezes D, Daumer KM, Lin Z, Witkiewicz AK (2010). Oxidative stress in cancer associated fibroblasts drives tumor-stroma co-evolution: a new paradigm for understanding tumor metabolism, the field effect and genomic instability in cancer cells. Cell Cycle.

[174] Shimura T, Sasatani M, Kawai H, Kamiya K, Kobayashi J, Komatsu K, Kunugita N (2018). Radiation-induced myofibroblasts promote tumor growth via mitochondrial ROS-activated TGFβ signaling. Mol Cancer Res: MCR.

[175] Cruz-Bermúdez A, Laza-Briviesca R, Vicente-Blanco RJ, García-Grande A, Coronado MJ, Laine-Menéndez S, Alfaro C, Sanchez JC, Franco F, Calvo V (2019). Cancer-associated fibroblasts modify lung cancer metabolism involving ROS and TGF-β signaling. Free Radical Biol Med.

[176] Ayala G, Morello M, Frolov A, You S, Li R, Rosati F, Bartolucci G, Danza G, Adam RM, Thompson TC (2013). Loss of caveolin-1 in prostate cancer stroma correlates with reduced relapse-free survival and is functionally relevant to tumour progression. J Pathol.

[177] Zhu Z, Achreja A, Meurs N, Animasahun O, Owen S, Mittal A, Parikh P, Lo TW, Franco-Barraza J, Shi J (2020). Tumour-reprogrammed stromal BCAT1 fuels branched-chain ketoacid dependency in stromal-rich PDAC tumours. Nat Metab.

[178] Leone RD, Powell JD (2020). Metabolism of immune cells in cancer. Nat Rev Cancer.

[179] Kohlgruber AC, LaMarche NM, Lynch L (2016). Adipose tissue at the nexus of systemic and cellular immunometabolism. Semin Immunol.

[180] Myers JA, Miller JS (2021). Exploring the NK cell platform for cancer immunotherapy. Nat Rev Clin Oncol.

[181] Terrén I, Orrantia A, Vitallé J, Astarloa-Pando G, Zenarruzabeitia O, Borrego F (2020). Modulating NK cell metabolism for cancer immunotherapy. Semin Hematol.

[182] Salzberger W, Martrus G, Bachmann K, Goebels H, Heß L, Koch M, Langeneckert A, Lunemann S, Oldhafer KJ, Pfeifer C (2018). Tissue-resident NK cells differ in their expression profile of the nutrient transporters Glut1, CD98 and CD71. PLoS ONE.

[183] Keating SE, Zaiatz-Bittencourt V, Loftus RM, Keane C, Brennan K, Finlay DK, Gardiner CM (2016). Metabolic reprogramming supports IFN-γ production by CD56bright NK cells. J Immunol (Baltimore, Md : 1950).

[184] Cong J, Wang X, Zheng X, Wang D, Fu B, Sun R, Tian Z, Wei H (2018). Dysfunction of natural killer cells by FBP1-induced inhibition of glycolysis during lung cancer progression. Cell Metab.

[185] Niavarani SR, Lawson C, Bakos O, Boudaud M, Batenchuk C, Rouleau S, Tai LH (2019). Lipid accumulation impairs natural killer cell cytotoxicity and tumor control in the postoperative period. BMC Cancer.

[186] Loftus RM, Assmann N, Kedia-Mehta N, O’Brien KL, Garcia A, Gillespie C, Hukelmann JL, Oefner PJ, Lamond AI, Gardiner CM (2018). Amino acid-dependent cMyc expression is essential for NK cell metabolic and functional responses in mice. Nat Commun.

[187] Nielsen SR, Schmid MC (2017). Macrophages as key drivers of cancer progression and metastasis. Mediators Inflamm.

[188] Wu FL, Nolan K, Strait AA, Bian L, Nguyen KA, Wang JH, Jimeno A, Zhou HM, Young CD, Wang XJ (2019). Macrophages promote growth of squamous cancer independent of T cells. J Dent Res.

[189] Liu D, Chang C, Lu N, Wang X, Lu Q, Ren X, Ren P, Zhao D, Wang L, Zhu Y (2017). Comprehensive proteomics analysis reveals metabolic reprogramming of tumor-associated macrophages stimulated by the tumor microenvironment. J Proteome Res.

[190] Arts RJW, Plantinga TS, Tuit S, Ulas T, Heinhuis B, Tesselaar M, Sloot Y, Adema GJ, Joosten LAB, Smit JWA (2016). Transcriptional and metabolic reprogramming induce an inflammatory phenotype in non-medullary thyroid carcinoma-induced macrophages. Oncoimmunology.

[191] Su P, Wang Q, Bi E, Ma X, Liu L, Yang M, Qian J, Yi Q (2020). Enhanced lipid accumulation and metabolism are required for the differentiation and activation of tumor-associated macrophages. Can Res.

[192] Jha AK, Huang SC, Sergushichev A, Lampropoulou V, Ivanova Y, Loginicheva E, Chmielewski K, Stewart KM, Ashall J, Everts B (2015). Network integration of parallel metabolic and transcriptional data reveals metabolic modules that regulate macrophage polarization. Immunity.

[193] Masucci MT, Minopoli M, Del Vecchio S, Carriero MV (2020). The emerging role of neutrophil extracellular traps (NETs) in tumor progression and metastasis. Front Immunol.

[194] Mollinedo F (2019). Neutrophil degranulation, plasticity, and cancer metastasis. Trends Immunol.

[195] Jaillon S, Ponzetta A, Di Mitri D, Santoni A, Bonecchi R, Mantovani A (2020). Neutrophil diversity and plasticity in tumour progression and therapy. Nat Rev Cancer.

[196] Jun HS, Weinstein DA, Lee YM, Mansfield BC, Chou JY (2014). Molecular mechanisms of neutrophil dysfunction in glycogen storage disease type Ib. Blood.

[197] Ancey PB, Contat C, Boivin G, Sabatino S, Pascual J, Zangger N, Perentes JY, Peters S, Abel ED, Kirsch DG (2021). GLUT1 expression in tumor-associated neutrophils promotes lung cancer growth and resistance to radiotherapy. Can Res.

[198] Rice CM, Davies LC, Subleski JJ, Maio N, Gonzalez-Cotto M, Andrews C, Patel NL, Palmieri EM, Weiss JM, Lee JM (2018). Tumour-elicited neutrophils engage mitochondrial metabolism to circumvent nutrient limitations and maintain immune suppression. Nat Commun.

[199] Veglia F, Sanseviero E, Gabrilovich DI (2021). Myeloid-derived suppressor cells in the era of increasing myeloid cell diversity. Nat Rev Immunol.

[200] Veglia F, Perego M, Gabrilovich D (2018). Myeloid-derived suppressor cells coming of age. Nat Immunol.

[201] Al-Khami AA, Rodriguez PC, Ochoa AC (2016). Metabolic reprogramming of myeloid-derived suppressor cells (MDSC) in cancer. Oncoimmunology.

[202] Al-Khami AA, Zheng L, Del Valle L, Hossain F, Wyczechowska D, Zabaleta J, Sanchez MD, Dean MJ, Rodriguez PC, Ochoa AC (2017). Exogenous lipid uptake induces metabolic and functional reprogramming of tumor-associated myeloid-derived suppressor cells. Oncoimmunology.

[203] Hossain F, Al-Khami AA, Wyczechowska D, Hernandez C, Zheng L, Reiss K, Valle LD, Trillo-Tinoco J, Maj T, Zou W (2015). Inhibition of fatty acid oxidation modulates immunosuppressive functions of myeloid-derived suppressor cells and enhances cancer therapies. Cancer Immunol Res.

[204] Balan S, Arnold-Schrauf C, Abbas A, Couespel N, Savoret J, Imperatore F, Villani AC, Vu Manh TP, Bhardwaj N, Dalod M (2018). Large-scale human dendritic cell differentiation revealing notch-dependent lineage bifurcation and heterogeneity. Cell Rep.

[205] Li Y, Wan YY, Zhu B (2017). Immune cell metabolism in tumor microenvironment. Adv Exp Med Biol.

[206] Gardner JK, Mamotte CDS, Patel P, Yeoh TL, Jackaman C, Nelson DJ (2015). Mesothelioma tumor cells modulate dendritic cell lipid content, phenotype and function. PLoS ONE.

[207] Peng X, He Y, Huang J, Tao Y, Liu S (2021). Metabolism of dendritic cells in tumor microenvironment: for immunotherapy. Front Immunol.

[208] Kouidhi S, Noman MZ, Kieda C, Elgaaied AB, Chouaib S (2016). Intrinsic and tumor microenvironment-induced metabolism adaptations of T cells and impact on their differentiation and function. Front Immunol.

[209] Michalek RD, Gerriets VA, Jacobs SR, Macintyre AN, MacIver NJ, Mason EF, Sullivan SA, Nichols AG, Rathmell JC (2011). Cutting edge: distinct glycolytic and lipid oxidative metabolic programs are essential for effector and regulatory CD4+ T cell subsets. J Immunol (Baltimore, Md: 1950).

[210] Sukumar M, Liu J, Ji Y, Subramanian M, Crompton JG, Yu Z, Roychoudhuri R, Palmer DC, Muranski P, Karoly ED (2013). Inhibiting glycolytic metabolism enhances CD8+ T cell memory and antitumor function. J Clin Investig.

[211] Ma X, Bi E, Lu Y, Su P, Huang C, Liu L, Wang Q, Yang M, Kalady MF, Qian J (2019). Cholesterol induces CD8(+) T cell exhaustion in the tumor microenvironment. Cell Metab.

[212] Molon B, Ugel S, Del Pozzo F, Soldani C, Zilio S, Avella D, De Palma A, Mauri P, Monegal A, Rescigno M (2011). Chemokine nitration prevents intratumoral infiltration of antigen-specific T cells. J Exp Med.

[213] Munn DH, Sharma MD, Baban B, Harding HP, Zhang Y, Ron D, Mellor AL (2005). GCN2 kinase in T cells mediates proliferative arrest and anergy induction in response to indoleamine 2,3-dioxygenase. Immunity.

[214] Kim J-w (2007). Gao P, Liu Y-C, Semenza GL, Dang CV: Hypoxia-inducible factor 1 and dysregulated c-Myc cooperatively induce vascular endothelial growth factor and metabolic switches hexokinase 2 and pyruvate dehydrogenase kinase 1. Mol Cell Biol.

[215] Osthus RC, Shim H, Kim S, Li Q, Reddy R, Mukherjee M, Xu Y, Wonsey D, Lee LA, Dang CV (2000). Deregulation of glucose transporter 1 and glycolytic gene expression by c-Myc. J Biol Chem.

[216] Scarpulla RC (2008). Transcriptional paradigms in mammalian mitochondrial biogenesis and function. Physiol Rev.

[217] Caro-Maldonado A, Wang R, Nichols AG, Kuraoka M, Milasta S, Sun LD, Gavin AL, Abel ED, Kelsoe G, Green DR (2014). Metabolic reprogramming is required for antibody production that is suppressed in anergic but exaggerated in chronically BAFF-exposed B cells. J Immunol (Baltimore, Md: 1950).

[218] Derynck R, Turley SJ, Akhurst RJ (2021). TGFβ biology in cancer progression and immunotherapy. Nat Rev Clin Oncol.

[219] Slattery K, Woods E, Zaiatz-Bittencourt V, Marks S, Chew S, Conroy M, Goggin C, MacEochagain C, Kennedy J, Lucas S (2021). TGFβ drives NK cell metabolic dysfunction in human metastatic breast cancer. J Immunother Cancer.

[220] Zaiatz-Bittencourt V, Finlay DK, Gardiner CM (2018). Canonical TGF-β signaling pathway represses human NK cell metabolism. J Immunol (Baltimore, Md : 1950).

[221] Park JE, Dutta B, Tse SW, Gupta N, Tan CF, Low JK, Yeoh KW, Kon OL, Tam JP, Sze SK (2019). Hypoxia-induced tumor exosomes promote M2-like macrophage polarization of infiltrating myeloid cells and microRNA-mediated metabolic shift. Oncogene.

[222] Dzik JM (2014). Evolutionary roots of arginase expression and regulation. Front Immunol.

[223] Arlauckas SP, Garren SB, Garris CS, Kohler RH, Oh J, Pittet MJ, Weissleder R (2018). Arg1 expression defines immunosuppressive subsets of tumor-associated macrophages. Theranostics.

[224] Gómez V, Eykyn TR, Mustapha R, Flores-Borja F, Male V, Barber PR, Patsialou A, Green R, Panagaki F, Li CW (2020). Breast cancer-associated macrophages promote tumorigenesis by suppressing succinate dehydrogenase in tumor cells. Sci Signal.

[225] Rotondo R, Barisione G, Mastracci L, Grossi F, Orengo AM, Costa R, Truini M, Fabbi M, Ferrini S, Barbieri O (2009). IL-8 induces exocytosis of arginase 1 by neutrophil polymorphonuclears in nonsmall cell lung cancer. Int J Cancer.

[226] Pang Y, Gara SK, Achyut BR, Li Z, Yan HH, Day CP, Weiss JM, Trinchieri G, Morris JC, Yang L (2013). TGF-β signaling in myeloid cells is required for tumor metastasis. Cancer Discov.

[227] Angioni R, Liboni C, Herkenne S, Sánchez-Rodríguez R, Borile G, Marcuzzi E, Calì B, Muraca M, Viola A (2020). CD73(+) extracellular vesicles inhibit angiogenesis through adenosine A(2B) receptor signalling. J Extracell Vesicles.

[228] Groth C, Hu X, Weber R, Fleming V, Altevogt P, Utikal J, Umansky V (2019). Immunosuppression mediated by myeloid-derived suppressor cells (MDSCs) during tumour progression. Br J Cancer.

[229] Li J, Wang L, Chen X, Li L, Li Y, Ping Y, Huang L, Yue D, Zhang Z, Wang F (2017). CD39/CD73 upregulation on myeloid-derived suppressor cells via TGF-β-mTOR-HIF-1 signaling in patients with non-small cell lung cancer. Oncoimmunology.

[230] Priyadharshini B, Loschi M, Newton RH, Zhang J-W, Finn KK, Gerriets VA, Huynh A, Rathmell JC, Blazar BR, Turka LA (2018). Cutting edge: TGF-β and phosphatidylinositol 3-kinase signals modulate distinct metabolism of regulatory T cell subsets. J Immunol (Baltimore, Md : 1950).

[231] Ho PC, Bihuniak JD, Macintyre AN, Staron M, Liu X, Amezquita R, Tsui YC, Cui G, Micevic G, Perales JC (2015). Phosphoenolpyruvate Is a Metabolic checkpoint of anti-tumor T cell responses. Cell.

[232] Cham CM, Driessens G, O'Keefe JP, Gajewski TF (2008). Glucose deprivation inhibits multiple key gene expression events and effector functions in CD8+ T cells. Eur J Immunol.

[233] Renner K, Bruss C, Schnell A, Koehl G, Becker HM, Fante M, Menevse AN, Kauer N, Blazquez R, Hacker L (2019). Restricting glycolysis preserves T cell effector functions and augments checkpoint therapy. Cell Rep.

[234] Cascone T, McKenzie JA, Mbofung RM, Punt S, Wang Z, Xu C, Williams LJ, Wang Z, Bristow CA, Carugo A (2018). Increased tumor glycolysis characterizes immune resistance to adoptive T cell therapy. Cell Metab.

[235] Dimeloe S, Gubser P, Loeliger J, Frick C, Develioglu L, Fischer M, Marquardsen F, Bantug GR, Thommen D, Lecoultre Y (2019). Tumor-derived TGF-β inhibits mitochondrial respiration to suppress IFN-γ production by human CD4(+) T cells. Sci Signal.

[236] Gasparics Á, Rosivall L, Krizbai IA, Sebe A (2016). When the endothelium scores an own goal: endothelial cells actively augment metastatic extravasation through endothelial-mesenchymal transition. Am J Physiol Heart Circ Physiol.

[237] Cantelmo AR, Conradi L-C, Brajic A, Goveia J, Kalucka J, Pircher A, Chaturvedi P, Hol J, Thienpont B, Teuwen L-A (2016). Inhibition of the glycolytic activator PFKFB3 in endothelium induces tumor vessel normalization, impairs metastasis, and improves chemotherapy. Cancer Cell.

[238] Zhang L, Li S, Li L, Chen Z, Yang Y (2018). COX-2 inhibition in the endothelium induces glucose metabolism normalization and impairs tumor progression. Mol Med Rep.

[239] Hinshaw DB, Burger JM (1990). Protective effect of glutamine on endothelial cell ATP in oxidant injury. J Surg Res.

[240] Ning J, Zhao Y, Ye Y, Yu J (2019). Opposing roles and potential antagonistic mechanism between TGF-β and BMP pathways: implications for cancer progression. EBioMedicine.

[241] Howell ED, Yzaguirre AD, Gao P, Lis R, He B, Lakadamyali M, Rafii S, Tan K, Speck NA (2021). Efficient hemogenic endothelial cell specification by RUNX1 is dependent on baseline chromatin accessibility of RUNX1-regulated TGFβ target genes. Genes Dev.

[242] Xiong J, Kawagishi H, Yan Y, Liu J, Wells QS, Edmunds LR, Fergusson MM, Yu ZX, Rovira II, Brittain EL (2018). A metabolic basis for endothelial-to-mesenchymal transition. Mol Cell.

[243] Goumans MJ, Ten Dijke P (2018). TGF-β signaling in control of cardiovascular function. Cold Spring Harb Perspect Biol.

[244] Armulik A, Genové G, Betsholtz C (2011). Pericytes: developmental, physiological, and pathological perspectives, problems, and promises. Dev Cell.

[245] Zonneville J, Safina A, Truskinovsky AM, Arteaga CL, Bakin AV (2018). TGF-β signaling promotes tumor vasculature by enhancing the pericyte-endothelium association. BMC Cancer.

[246] Yeh WL, Lin CJ, Fu WM (2008). Enhancement of glucose transporter expression of brain endothelial cells by vascular endothelial growth factor derived from glioma exposed to hypoxia. Mol Pharmacol.

[247] Wenes M, Shang M, Di Matteo M, Goveia J, Martín-Pérez R, Serneels J, Prenen H, Ghesquière B, Carmeliet P, Mazzone M (2016). Macrophage metabolism controls tumor blood vessel morphogenesis and metastasis. Cell Metab.

[248] Guo Y, Deng Y, Li X, Ning Y, Lin X, Guo S, Chen M, Han M (2016). Glutaminolysis was induced by TGF-β1 through PP2Ac regulated Raf-MEK-ERK signaling in endothelial cells. PLoS ONE.

[249] Nieman KM, Kenny HA, Penicka CV, Ladanyi A, Buell-Gutbrod R, Zillhardt MR, Romero IL, Carey MS, Mills GB, Hotamisligil GS (2011). Adipocytes promote ovarian cancer metastasis and provide energy for rapid tumor growth. Nat Med.

[250] Clement E, Lazar I, Attané C, Carrié L, Dauvillier S, Ducoux-Petit M, Esteve D, Menneteau T, Moutahir M, Le Gonidec S (2020). Adipocyte extracellular vesicles carry enzymes and fatty acids that stimulate mitochondrial metabolism and remodeling in tumor cells. EMBO J.

[251] Seo J, Kim KS, Park JW, Cho JY, Chang H, Fukuda J, Hong KY, Chun YS (2021). Metastasis-on-a-chip reveals adipocyte-derived lipids trigger cancer cell migration via HIF-1α activation in cancer cells. Biomaterials.

[252] Karsten E, Breen E, McCracken SA, Clarke S, Herbert BR (2020). Red blood cells exposed to cancer cells in culture have altered cytokine profiles and immune function. Sci Rep.

[253] Hercbergs A, Brok-Simoni F, Holtzman F, Bar-Am J, Leith JT, Brenner HJ (1992). Erythrocyte glutathione and tumour response to chemotherapy. Lancet.

[254] Sharma A, Smyrk TC, Levy MJ, Topazian MA, Chari ST (2018). Fasting blood glucose levels provide estimate of duration and progression of pancreatic cancer Before diagnosis. Gastroenterology.

[255] Lee J-H, Mellado-Gil JM, Bahn YJ, Pathy SM, Zhang YE, Rane SG (2020). Protection from β-cell apoptosis by inhibition of TGF-β/Smad3 signaling. Cell Death Dis.

[256] Zhao J, Liang Y, Yin Q, Liu S, Wang Q, Tang Y, Cao C (2016). Clinical and prognostic significance of serum transforming growth factor-beta1 levels in patients with pancreatic ductal adenocarcinoma. Braz J Med Biol Res.

[257] Yadav H, Devalaraja S, Chung ST, Rane SG (2017). TGF-β1/Smad3 pathway targets PP2A-AMPK-FoxO1 signaling to regulate hepatic gluconeogenesis. J Biol Chem.

[258] Fearon K, Strasser F, Anker SD, Bosaeus I, Bruera E, Fainsinger RL, Jatoi A, Loprinzi C, MacDonald N, Mantovani G (2011). Definition and classification of cancer cachexia: an international consensus. Lancet Oncol.

[259] Pin F, Barreto R, Couch ME, Bonetto A, O'Connell TM (2019). Cachexia induced by cancer and chemotherapy yield distinct perturbations to energy metabolism. J Cachexia Sarcopenia Muscle.

[260] Penna F, Costamagna D, Pin F, Camperi A, Fanzani A, Chiarpotto EM, Cavallini G, Bonelli G, Baccino FM, Costelli P (2013). Autophagic degradation contributes to muscle wasting in cancer cachexia. Am J Pathol.

[261] Penna F, Ballarò R, Martinez-Cristobal P, Sala D, Sebastian D, Busquets S, Muscaritoli M, Argilés JM, Costelli P, Zorzano A (2019). Autophagy exacerbates muscle wasting in cancer cachexia and impairs mitochondrial function. J Mol Biol.

[262] Yang X, Xue P, Liu X, Xu X, Chen Z (2018). HMGB1/autophagy pathway mediates the atrophic effect of TGF-β1 in denervated skeletal muscle. Cell Commun Signal.

[263] Greco SH, Tomkötter L, Vahle AK, Rokosh R, Avanzi A, Mahmood SK, Deutsch M, Alothman S, Alqunaibit D, Ochi A (2015). TGF-β blockade reduces mortality and metabolic changes in a validated murine model of pancreatic cancer cachexia. PLoS ONE.

[264] Lacouture ME, Morris JC, Lawrence DP, Tan AR, Olencki TE, Shapiro GI, Dezube BJ, Berzofsky JA, Hsu FJ, Guitart J (2015). Cutaneous keratoacanthomas/squamous cell carcinomas associated with neutralization of transforming growth factor β by the monoclonal antibody fresolimumab (GC1008). Cancer Immunol Immunother.

[265] Lacouture ME, Morris JC, Lawrence DP, Tan AR, Olencki TE, Shapiro GI, Dezube BJ, Berzofsky JA, Hsu FJ, Guitart J (2015). Cutaneous keratoacanthomas/squamous cell carcinomas associated with neutralization of transforming growth factor β by the monoclonal antibody fresolimumab (GC1008). Cancer Immunol Immunother: CII.

[266] Osumi H, Horiguchi H, Kadomatsu T, Tashiro K, Morinaga J, Takahashi T, Ikeda K, Ito T, Suzuki M, Endo M (2020). Tumor cell-derived angiopoietin-like protein 2 establishes a preference for glycolytic metabolism in lung cancer cells. Cancer Sci.

[267] Cheng KY, Hao M (2017). Mammalian target of rapamycin (mTOR) regulates transforming growth factor-β1 (TGF-β1)-induced epithelial-mesenchymal transition via decreased pyruvate kinase M2 (PKM2) expression in cervical cancer cells. Med Sci Monit.

[268] Haidar M, Metheni M, Batteux F, Langsley G (2019). TGF-β2, catalase activity, H(2)O(2) output and metastatic potential of diverse types of tumour. Free Radical Biol Med.

[269] Zhao Y, Xia S, Cao C, Du X (2019). Effect of TGF-β1 on apoptosis of colon cancer cells via the ERK signaling pathway. J BUON.

[270] Soukupova J, Malfettone A, Bertran E, Hernández-Alvarez MI, Peñuelas-Haro I, Dituri F, Giannelli G, Zorzano A, Fabregat I (2021). Epithelial-mesenchymal transition (EMT) induced by TGF-β in hepatocellular carcinoma cells reprograms lipid metabolism. Int J Mol Sci.

[271] Flaig TW, Glode M, Gustafson D, van Bokhoven A, Tao Y, Wilson S, Su LJ, Li Y, Harrison G, Agarwal R (2010). A study of high-dose oral silybin-phytosome followed by prostatectomy in patients with localized prostate cancer. Prostate.

[272] Guo Z, Cheng Z, Wang J, Liu W, Peng H, Wang Y, Rao AVS, Li RJ, Ying X, Korangath P (2019). Discovery of a potent GLUT inhibitor from a library of rapafucins by using 3D microarrays. Angew Chem Int Ed Engl.

[273] Lord SR, Collins JM, Cheng WC, Haider S, Wigfield S, Gaude E, Fielding BA, Pinnick KE, Harjes U, Segaran A (2020). Transcriptomic analysis of human primary breast cancer identifies fatty acid oxidation as a target for metformin. Br J Cancer.

[274] Stein M, Lin H, Jeyamohan C, Dvorzhinski D, Gounder M, Bray K, Eddy S, Goodin S, White E, Dipaola RS (2010). Targeting tumor metabolism with 2-deoxyglucose in patients with castrate-resistant prostate cancer and advanced malignancies. Prostate.

[275] Sun X, Sun G, Huang Y, Hao Y, Tang X, Zhang N, Zhao L, Zhong R, Peng Y (2020). 3-Bromopyruvate regulates the status of glycolysis and BCNU sensitivity in human hepatocellular carcinoma cells. Biochem Pharmacol.

[276] Yang Q, Miyagawa M, Liu X, Zhu B, Munemasa S, Nakamura T, Murata Y, Nakamura Y (2018). Methyl-β-cyclodextrin potentiates the BITC-induced anti-cancer effect through modulation of the Akt phosphorylation in human colorectal cancer cells. Biosci Biotechnol Biochem.

[277] McNeillis R, Greystoke A, Walton J, Bacon C, Keun H, Siskos A, Petrides G, Leech N, Jenkinson F, Bowron A (2020). A case of malignant hyperlactaemic acidosis appearing upon treatment with the mono-carboxylase transporter 1 inhibitor AZD3965. Br J Cancer.

[278] Chu QS, Sangha R, Spratlin J, Vos LJ, Mackey JR, McEwan AJ, Venner P, Michelakis ED (2015). A phase I open-labeled, single-arm, dose-escalation, study of dichloroacetate (DCA) in patients with advanced solid tumors. Invest New Drugs.

[279] Dai Y, Xiong X, Huang G, Liu J, Sheng S, Wang H, Qin W (2014). Dichloroacetate enhances adriamycin-induced hepatoma cell toxicity in vitro and in vivo by increasing reactive oxygen species levels. PLoS ONE.

[280] Anwar S, Mohammad T, Shamsi A, Queen A, Parveen S, Luqman S, Hasan GM, Alamry KA, Azum N, Asiri AM (2020). Discovery of hordenine as a potential inhibitor of pyruvate dehydrogenase kinase 3: implication in lung cancer therapy. Biomedicines.

[281] Mellinghoff IK, Ellingson BM, Touat M, Maher E, De La Fuente MI, Holdhoff M, Cote GM, Burris H, Janku F, Young RJ (2020). Ivosidenib in isocitrate dehydrogenase 1-mutated advanced glioma. J Clin Oncol.

[282] Abou-Alfa GK, Macarulla T, Javle MM, Kelley RK, Lubner SJ, Adeva J, Cleary JM, Catenacci DV, Borad MJ, Bridgewater J (2020). Ivosidenib in IDH1-mutant, chemotherapy-refractory cholangiocarcinoma (ClarIDHy): a multicentre, randomised, double-blind, placebo-controlled, phase 3 study. Lancet Oncol.

[283] Tong Z, Atsriku C, Yerramilli U, Wang X, Li Y, Reyes J, Fan B, Yang H, Hoffmann M, Surapaneni S (2019). Absorption, distribution, metabolism and excretion of an isocitrate dehydrogenase-2 inhibitor enasidenib in rats and humans. Xenobiotica.

[284] Stein EM, DiNardo CD, Pollyea DA, Fathi AT, Roboz GJ, Altman JK, Stone RM, DeAngelo DJ, Levine RL, Flinn IW (2017). Enasidenib in mutant IDH2 relapsed or refractory acute myeloid leukemia. Blood.

[285] Chen J, Yang J, Cao P (2016). The evolving landscape in the development of isocitrate dehydrogenase mutant inhibitors. Mini Rev Med Chem.

[286] Caravella JA, Lin J, Diebold RB, Campbell AM, Ericsson A, Gustafson G, Wang Z, Castro J, Clarke A, Gotur D (2020). Structure-based design and identification of FT-2102 (Olutasidenib), a potent mutant-selective IDH1 inhibitor. J Med Chem.

[287] Konteatis Z, Artin E, Nicolay B, Straley K, Padyana AK, Jin L, Chen Y, Narayaraswamy R, Tong S, Wang F (2020). Vorasidenib (AG-881): a first-in-class, brain-penetrant dual inhibitor of mutant IDH1 and 2 for treatment of glioma. ACS Med Chem Lett.

[288] Lee WJ, Chen WK, Wang CJ, Lin WL, Tseng TH (2008). Apigenin inhibits HGF-promoted invasive growth and metastasis involving blocking PI3K/Akt pathway and beta 4 integrin function in MDA-MB-231 breast cancer cells. Toxicol Appl Pharmacol.

[289] Shukla S, Gupta S (2007). Apigenin-induced cell cycle arrest is mediated by modulation of MAPK, PI3K-Akt, and loss of cyclin D1 associated retinoblastoma dephosphorylation in human prostate cancer cells. Cell Cycle.

[290] Fang J, Zhou Q, Liu LZ, Xia C, Hu X, Shi X, Jiang BH (2007). Apigenin inhibits tumor angiogenesis through decreasing HIF-1alpha and VEGF expression. Carcinogenesis.

[291] Fang J, Xia C, Cao Z, Zheng JZ, Reed E, Jiang BH (2005). Apigenin inhibits VEGF and HIF-1 expression via PI3K/AKT/p70S6K1 and HDM2/p53 pathways. FASEB J.

[292] Tang W, Zhao G (2020). Small molecules targeting HIF-1α pathway for cancer therapy in recent years. Bioorg Med Chem.

[293] Mita MM, Rowinsky EK, Forero L, Eckhart SG, Izbicka E, Weiss GR, Beeram M, Mita AC, de Bono JS, Tolcher AW (2007). A phase II, pharmacokinetic, and biologic study of semaxanib and thalidomide in patients with metastatic melanoma. Cancer Chemother Pharmacol.

[294] Ricker JL, Chen Z, Yang XP, Pribluda VS, Swartz GM, Van Waes C (2004). 2-methoxyestradiol inhibits hypoxia-inducible factor 1alpha, tumor growth, and angiogenesis and augments paclitaxel efficacy in head and neck squamous cell carcinoma. Clin Cancer Res.

[295] Mooberry SL (2003). Mechanism of action of 2-methoxyestradiol: new developments. Drug Resist Updat.

[296] Sibonga JD, Lotinun S, Evans GL, Pribluda VS, Green SJ, Turner RT (2003). Dose-response effects of 2-methoxyestradiol on estrogen target tissues in the ovariectomized rat. Endocrinology.

[297] Zhu Y, Zang Y, Zhao F, Li Z, Zhang J, Fang L, Li M, Xing L, Xu Z, Yu J (2017). Inhibition of HIF-1α by PX-478 suppresses tumor growth of esophageal squamous cell cancer in vitro and in vivo. Am J Cancer Res.

[298] Koh MY, Spivak-Kroizman T, Venturini S, Welsh S, Williams RR, Kirkpatrick DL, Powis G (2008). Molecular mechanisms for the activity of PX-478, an antitumor inhibitor of the hypoxia-inducible factor-1alpha. Mol Cancer Ther.

[299] Lee K, Kim HM (2011). A novel approach to cancer therapy using PX-478 as a HIF-1α inhibitor. Arch Pharm Res.

[300] Li YL, Rao MJ, Zhang NY, Wu LW, Lin NM, Zhang C (2019). BAY 87–2243 sensitizes hepatocellular carcinoma Hep3B cells to histone deacetylase inhibitors treatment via GSK-3β activation. Exp Ther Med.

[301] Schöckel L, Glasauer A, Basit F, Bitschar K, Truong H, Erdmann G, Algire C, Hägebarth A, Willems PH, Kopitz C (2015). Targeting mitochondrial complex I using BAY 87–2243 reduces melanoma tumor growth. Cancer Metab.

[302] Ellinghaus P, Heisler I, Unterschemmann K, Haerter M, Beck H, Greschat S, Ehrmann A, Summer H, Flamme I, Oehme F (2013). BAY 87–2243, a highly potent and selective inhibitor of hypoxia-induced gene activation has antitumor activities by inhibition of mitochondrial complex I. Cancer Med.

[303] Oudard S, Carpentier A, Banu E, Fauchon F, Celerier D, Poupon MF, Dutrillaux B, Andrieu JM, Delattre JY (2003). Phase II study of lonidamine and diazepam in the treatment of recurrent glioblastoma multiforme. J Neurooncol.

[304] Nath K, Guo L, Nancolas B, Nelson DS, Shestov AA, Lee SC, Roman J, Zhou R, Leeper DB, Halestrap AP (2016). Mechanism of antineoplastic activity of lonidamine. Biochem Biophys Acta.

[305] Rutkowski K, Sowa P, Rutkowska-Talipska J, Kuryliszyn-Moskal A, Rutkowski R (2014). Dehydroepiandrosterone (DHEA): hypes and hopes. Drugs.

[306] Mele L, la Noce M, Paino F, Regad T, Wagner S, Liccardo D, Papaccio G, Lombardi A, Caraglia M, Tirino V (2019). Glucose-6-phosphate dehydrogenase blockade potentiates tyrosine kinase inhibitor effect on breast cancer cells through autophagy perturbation. J Exp Clin Cancer Res: CR.

[307] O'Donovan TR, Rajendran S, O'Reilly S, O'Sullivan GC, McKenna SL (2015). Lithium modulates autophagy in esophageal and colorectal cancer cells and enhances the efficacy of therapeutic agents in vitro and in vivo. PLoS ONE.

[308] Huang K, Liang Q, Zhou Y, Jiang LL, Gu WM, Luo MY, Tang YB, Wang Y, Lu W, Huang M (2019). A novel allosteric inhibitor of phosphoglycerate mutase 1 suppresses growth and metastasis of non-small-cell lung cancer. Cell Metab.

[309] Hitosugi T, Zhou L, Elf S, Fan J, Kang HB, Seo JH, Shan C, Dai Q, Zhang L, Xie J, et al. Phosphoglycerate mutase 1 coordinates glycolysis and biosynthesis to promote tumor growth. Cancer Cell. 2012;22(5):585–600. 10.1016/j.ccr.2012.09.020 PMC3500524 23153533

[310] Cotte AK, Aires V, Fredon M, Limagne E, Derangère V, Thibaudin M, Humblin E, Scagliarini A, de Barros JP, Hillon P (2018). Lysophosphatidylcholine acyltransferase 2-mediated lipid droplet production supports colorectal cancer chemoresistance. Nat Commun.

[311] Yen MC, Kan JY, Hsieh CJ, Kuo PL, Hou MF, Hsu YL (2017). Association of long-chain acyl-coenzyme a synthetase 5 expression in human breast cancer by estrogen receptor status and its clinical significance. Oncol Rep.

[312] Ayyagari VN, Wang X, Diaz-Sylvester PL, Groesch K, Brard L (2020). Assessment of acyl-CoA cholesterol acyltransferase (ACAT-1) role in ovarian cancer progression-an in vitro study. PLoS ONE.

[313] Ali A, Levantini E, Teo JT, Goggi J, Clohessy JG, Wu CS, Chen L, Yang H, Krishnan I, Kocher O (2018). Fatty acid synthase mediates EGFR palmitoylation in EGFR mutated non-small cell lung cancer. EMBO Mol Med.

[314] Chang L, Fang S, Chen Y, Yang Z, Yuan Y, Zhang J, Ye L, Gu W (2019). Inhibition of FASN suppresses the malignant biological behavior of non-small cell lung cancer cells via deregulating glucose metabolism and AKT/ERK pathway. Lipids Health Dis.

[315] Falchook G, Infante J, Arkenau HT, Patel MR, Dean E, Borazanci E, Brenner A, Cook N, Lopez J, Pant S (2021). First-in-human study of the safety, pharmacokinetics, and pharmacodynamics of first-in-class fatty acid synthase inhibitor TVB-2640 alone and with a taxane in advanced tumors. EClinicalMedicine.

[316] Gouw AM, Eberlin LS, Margulis K, Sullivan DK, Toal GG, Tong L, Zare RN, Felsher DW (2017). Oncogene KRAS activates fatty acid synthase, resulting in specific ERK and lipid signatures associated with lung adenocarcinoma. Proc Natl Acad Sci USA.

[317] Zhang T, Bai R, Wang Q, Wang K, Li X, Liu K, Ryu J, Wang T, Chang X, Ma W (2019). Fluvastatin inhibits HMG-CoA reductase and prevents non-small cell lung carcinogenesis. Cancer Prev Res.

[318] Varghese S, Pramanik S, Williams LJ, Hodges HR, Hudgens CW, Fischer GM, Luo CK, Knighton B, Tan L, Lorenzi PL (2021). The glutaminase inhibitor CB-839 (Telaglenastat) enhances the antimelanoma activity of T-cell-mediated immunotherapies. Mol Cancer Ther.

[319] Zhou WX, Chen C, Liu XQ, Li Y, Lin YL, Wu XT, Kong LY, Luo JG (2021). Discovery and optimization of withangulatin a derivatives as novel glutaminase 1 inhibitors for the treatment of triple-negative breast cancer. Eur J Med Chem.

